# Genome-wide association study of rice (*Oryza sativa* L.) leaf traits with a high-throughput leaf scorer

**DOI:** 10.1093/jxb/erv100

**Published:** 2015-03-20

**Authors:** Wanneng Yang, Zilong Guo, Chenglong Huang, Ke Wang, Ni Jiang, Hui Feng, Guoxing Chen, Qian Liu, Lizhong Xiong

**Affiliations:** ^1^National Key Laboratory of Crop Genetic Improvement and National Center of Plant Gene Research (Wuhan), Huazhong Agricultural University, Wuhan 430070, PR China; ^2^College of Engineering, Huazhong Agricultural University, Wuhan 430070, PR China; ^3^Agricultural Bioinformatics Key Laboratory of Hubei Province, Huazhong Agricultural University, Wuhan 430070, PR China; ^4^Britton Chance Center for Biomedical Photonics, Wuhan National Laboratory for Optoelectronics, Huazhong University of Science and Technology, 1037 Luoyu Road, Wuhan 430074, PR China; ^5^MOA Key Laboratory of Crop Ecophysiology and Farming System in the Middle Reaches of the Yangtze River, College of Plant Science and Technology, Huazhong Agricultural University, Wuhan, Hubei 430070, PR China

**Keywords:** GWAS, high-throughput leaf scorer, leaf colour, leaf shape, leaf size, plant phenomics.

## Abstract

Leaves are the plant’s solar panel and food factory, and leaf traits are always key issues to investigate in plant research. Traditional methods for leaf trait measurement are time-consuming. In this work, an engineering prototype has been established for high-throughput leaf scoring (HLS) of a large number of *Oryza sativa* accessions. The mean absolute per cent of errors in traditional measurements versus HLS were below 5% for leaf number, area, shape, and colour. Moreover, HLS can measure up to 30 leaves per minute. To demonstrate the usefulness of HLS in dissecting the genetic bases of leaf traits, a genome-wide association study (GWAS) was performed for 29 leaf traits related to leaf size, shape, and colour at three growth stages using HLS on a panel of 533 rice accessions. Nine associated loci contained known leaf-related genes, such as *Nal1* for controlling the leaf width. In addition, a total of 73, 123, and 177 new loci were detected for traits associated with leaf size, colour, and shape, respectively. In summary, after evaluating the performance with a large number of rice accessions, the combination of GWAS and high-throughput leaf phenotyping (HLS) has proven a valuable strategy to identify the genetic loci controlling rice leaf traits.

## Highlight

A combination of high-throughput leaf phenotyping and genome-wide association analysis provides valuable insights into the genetic basis of rice leaf traits.

## Introduction

Leaves are primarily involved in photosynthesis and transpiration, influencing yield performance in crops (P Wang *et al.*, 2011). The size, shape, and number of leaves determine a plant’s photosynthetic potential and play important roles in determining plant yield, disease resistance, and stress responses (Pérez-Pérez *et al.*, 2010). In addition to these traits, the colour (or degree of leaf greenness) is also an important trait related to the leaf nitrogen status (Singh *et al.*, 2002). Because rice (*Oryza sativa*) is a staple food for billions of people throughout the world, particularly in Asia, Africa, and Latin America (Zhang, 2007), it is necessary to systematically study the genetic basis of leaf traits in rice.

Many previous studies have uncovered how the leaf size and shape is controlled from the perspective of leaf development (Moon and Hakes, 2011; Tsukaya, 2003). Leaf development starts with the recruitment of founder cells from the shoot apical meristem, which is characterized by the down-regulation of *knotted1*-like homeobox genes (Byrne, 2005). The over-expression of five of these genes (*OsH1*, *OsH6*, *OsH15*, *OsH71*, and *OsH43*) in rice profoundly affects leaf formation and results in severely malformed leaves (Sentoku *et al.*, 2000). WUSCHEL-related homeobox genes also play an important role in recruiting founder cells as a meristem organizer (Kessler and Sinha, 2004). In rice, two WUSCHEL-related homeobox genes, namely *NAL2* and *NAL3* (*NAL2/3*), affect leaf lateral-axis outgrowth and leaf width (LW) (Cho *et al.*, 2013). After recruitment, several genes act to establish polarity in developing leaves, of which the class III homeodomain leucine zipper (HD-Zip III) genes specify leaf adaxial identity (Kessler and Sinha, 2004). OsHB genes are members of the rice HD-ZIP III gene family, and the ectopic expression of *OsH1*, *OsH3*, and *OsH5* results in rolled and filamentous leaves (Itoh *et al.*, 2008). In addition, some genes associated with leaf size and shape have been identified via map-based cloning in rice. For example, the mutant *nal1* exhibits reduced LW, a decreased number of longitudinal veins, and a defective vascular system. The mutant *nal1* encodes a plant-specific protein that affects polar auxin transport and vascular patterns (Qi *et al.*, 2008). *NRL1* and *NAL7*, which are associated with leaf size and shape, were cloned and characterized using the same approach (Fujino *et al.*, 2008; Hu *et al.*, 2010).

In comparison with leaf size and shape, there is less research on leaf colour in rice. The mutant *yellow green leaf 1* (*ygl1*), a spontaneous mutant isolated from indica rice ‘Zhenhui 249’, exhibits a yellow-green leaf phenotype. This phenotype is caused by a mutation in the *YGL1* gene encoding the chlorophyll synthase as demonstrated via map-based cloning and genetic complementation analyses (Wu *et al.*, 2007). Another mutant *yellow green leaf 7* (*ygl7*) is also characterized by a yellow-green leaf phenotype during its whole lifespan and results from a mutation in *YGL7*, which encodes a magnesium-chelatase ChlD protein (Deng *et al.*, 2014). As an alternative approach, some quantitative trait loci (QTL) have been mapped for leaf size, shape, and colour (Farooq, *et al.*, 2010; P Wang *et al.*, 2011; Yue *et al.*, 2006). However, the genetic basis of leaf traits is largely unclear because only a limited number of leaf traits such as leaf length (LL) and LW have been studied. To our knowledge, a genome-wide association study (GWAS) of leaf angle, LL, and LW to dissect the genetic architecture has only been performed in maize (Tian *et al.*, 2011); no GWAS of these leaf traits have been conducted on the abundant natural variation in plants with multiple tillers such as rice. It is necessary to develop a reliable, automatic, and high-throughput scorer for leaf size, shape, and colour evaluations of rice and other crops.

Current estimation methods for the leaf area or leaf area index (LAI, which is defined as the ratio of the leaf area to a given unit of land area) are either non-destructive or destructive. The non-destructive methods include a canopy analyser (e.g. LAI-2000, LI-COR, Lincoln, NE, USA) (Sone *et al.*, 2009; Wijk and Williams, 2005); digital camera (Shibayama *et al.*, 2011a); near-infrared digital camera (Shibayama *et al.*, 2011b); and hyperspectral bands (F Wang *et al.*, 2011), which have been adopted to estimate or predict the LAI in fields. However, when measuring all the leaves on each individual plant, these methods are not suitable because of their inefficiency and inaccuracy. If using a desktop scanner (DS), the leaf area per plant can be extracted using a subsequent image analysis (Bakr, 2005; Bylesjö *et al.*, 2008; O’Neal *et al.*, 2002); however, this method is inefficient because each clipped leaf must be scanned individually. Even if the leaves are pasted on white paper for scanning, the efficiency is not satisfactory because the leaf pasting is also time-consuming and labour-intensive, particularly when dealing with curly leaves resulting from dehydration. Besides measuring leaf size and shape, the traditional evaluation of leaf colour using the leaf colour chart (developed by the International Rice Research Institute, or IRRI) is inefficient, especially when processing a large set of samples.

A high-throughput rice phenotyping facility as recently established that can simultaneously measure multiple morphological traits in rice, including the leaf area per plant, in a non-destructive manner (Yang *et al.*, 2014). However, there is also a need for a high-throughput and high-accuracy tool to extract almost all the leaf traits we can define, which is a very important complementary tool to the high-throughput rice phenotyping facility in the rice phenotyping platform. Here, a reliable, automatic high-throughput leaf scorer (HLS) is presented for leaf trait evaluations of a large number of rice accessions by using photonics vision-based techniques. The performance of the HLS for leaf number, area, colour, and shape, was evaluated. Moreover, 29 leaf traits at three different growth stages were extracted with the HLS system, and GWASs of these traits were performed. The results demonstrate that a combination of GWAS and HLS can provide valuable insight into the genetic architecture of leaf traits.

## Materials and methods

### Plant material

Detailed information on the rice accessions and genotyping were described in a previous study (Chen *et al.*, 2014). To test the HLS’s performance in measuring green leaf area (GLA), total leaf area (TLA), and leaf number (LN), 163 rice accessions were randomly selected. To evaluate the measuring accuracy of LL, LW, and green leaf colour discrimination of the HLS system, three batches of rice leaves (50 leaves for each batch,) were selected using the leaf colour chart developed by the IRRI: green-2 (light green), green-3 (moderate green), and green-4 (dark green). Each batch was then measured with the HLS. Simultaneously, the LL and LW of 150 leaves were measured manually. Moreover, 455 accessions at late tillering stage, 469 at late booting stage, and 389 at milk grain stage were phenotyped with HLS. The experimental arrangement of the rice material is shown in Supplementary Fig. S1.

### Constructing the HLS system

**Fig. 1.**
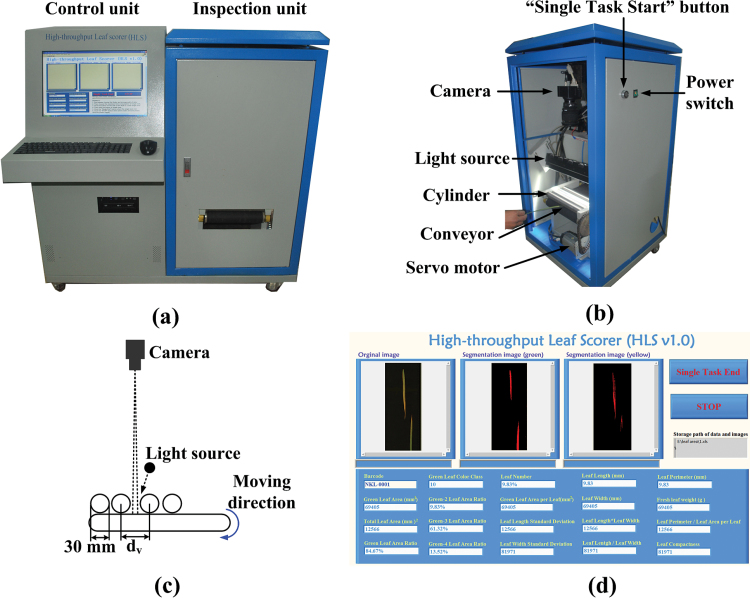
**The design of the HLS system. (a**) The developed prototype of the HLS; **(b)** details of the inspection unit; **(c)** the configuration drawing of the inspection unit; and **(d)** the software interface.

The HLS system contains two primary elements: a control unit and an inspection unit (Fig. 1a). The control unit is designed for user interaction, system control, image analysis, and data management. The inspection unit is designed for leaf feeding, image scanning, and image transmission. The control unit includes the following three primary devices: a workstation (HP xw6400, Hewlett-Packard Development Company, USA), a camera-link image acquisition device (IMAQ PCI-1428, National Instruments Corporation, USA) that digitizes the images into 3-colour 8-bit files, and a programmable logic controller (PLC, CP1H-Y20DT-D, Omron Corporation, Japan). The details of the operation procedure, data management, and control unit are introduced in Supplementary Video S1, Supplementary Fig. S2, Supplementary Fig. S3, and Supplementary Notes.

#### The inspection unit

The inspection unit contains a camera, a light source, and a leaf feeding device; the details are shown in Fig. 1b. A colour line-scan camera (TAPIX LCD 2048@9000, Tattile International Ltd., Italy), which is equipped with a 28mm lens (F1.8 EX DG Aspherical Macro, SIGMA, Japan), is used to acquire a colour image with 1280×3000 (horizontal × vertical) pixels. A line-array light source (BL-494-40-W, Cogstek Automation Technology Co. Ltd., China; set to 26.4V and 0.9 A) is fixed to provide stable illumination for the imaging system. A feeding device (shown in Fig. 1b), which includes four stainless steel cylinders and a conveyor, transports the leaves automatically (even if the leaves are curly). The conveyor is driven by a servo motor (MHMD042P1U, Panasonic Corporation, Japan). Finally, all the scanned leaves are transferred and gathered in a collecting box.

The configuration of the inspection unit is shown in Fig. 1c. The pixel size of the camera is 14×14 μm, and the spatial resolution of the inspection system is 0.22×0.22mm (0.048mm^2^/pixel), which is a magnification of ~15.71. The efficient image size of the line-scan camera is 17.94mm × 0.24mm; therefore, the field of view (FOV) of the inspection unit is 281.84mm × 3.77mm. To ensure that there is a sufficient image FOV with the diameter of the cylinder (30mm), the centre-line space between the second cylinder and the third cylinder (*d*_*v*_) must be designed to be more than 33.77mm. In the inspection unit, the *d*_*v*_ is designed to measure 45mm.

The software interface (designed by LabVIEW, National Instruments Corporation, USA) is shown in Fig. 1d. The original image and green/yellow segmentation image, along with the barcode and 29 leaf traits (defined in Supplementary Table S1), are displayed on the interface in real time.

#### Image analysis

**Fig. 2.**
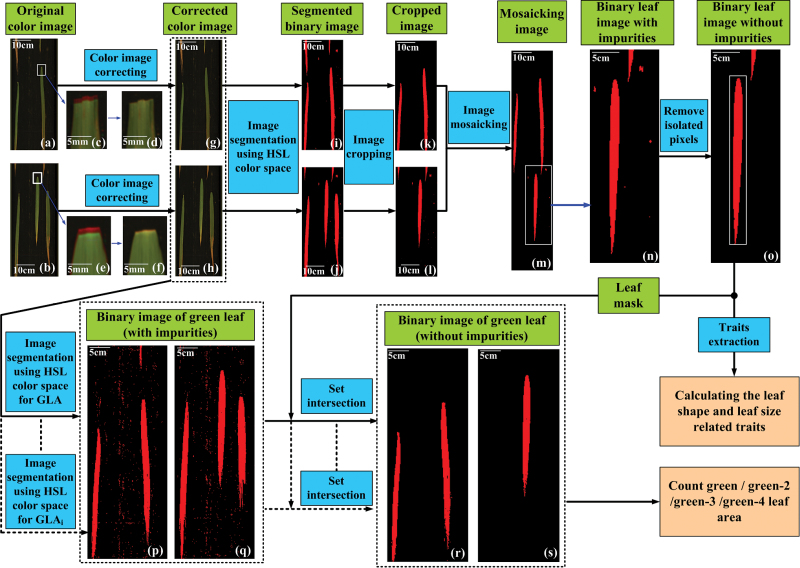
**The primary procedure for the image analysis. (a, b**) The distorted images in RGB colour space of two adjoining images. **(c, e)** The distorted partially enlarged images. **(d, f)** The corrected partially enlarged images. **(g, h)** The corrected images related to a and b. **(i, j)** The segmented images of the two adjoining images. **(k, l)** After image cropping, the cut partial image in the previous image and remaining leaf portion in the next image. **(m)** The mosaicking image. **(n)** The partially enlarged image with impurities. **(o)** The binary leaf image without impurities. **(p, q)** The binary image of a green (or green-2, green-3, green-4) leaf with impurities. **(r, s)** After setting the intersection, the binary image of a green (or green-2, green-3, green-4) leaf without impurities.

The colour line-scan camera consists of three monochrome chips, which are fixed in parallel to trap the red, green, and blue (RGB) spectra, respectively. Thus, the original colour image is distorted in RGB colour space (Fig. 2c, e). After the three greyscale images (RGB components) are extracted using a predefined value, the greyscale images of the green and blue components are shifted up a level with the position of the red greyscale image. Finally, the three greyscale images are merged back together to form a corrected RGB colour image (Fig. 2d, f). The source code for image correction is introduced in Supplementary Notes.

After the RGB colour image is corrected, the image analysis procedures for the two adjoining images (Fig. 2g, h) include the following steps: (i) With the predefined segmentation values in hue, saturation, and illumination (HSL) colour space, the adjoining colour images are transferred to the binary images (Fig. 2i, j); (ii) the leaves part in the previous image, and they are connected to the lower boundary (Fig. 2k), cut, and reconnected to the remaining leaf portion in the next image (Fig. 2l); (iii) some dust may appear on the mosaic image (Fig. 2m), so after removing the isolated pixels, the binary leaf image without impurities can be extracted (Fig. 2o); (iv) the morphological traits of each leaf, including the LL, LW, leaf perimeter, leaf area, and leaf compactness can be extracted; (v) after the images (Fig. 2g, h) are segmented with predefined values in HSL colour space for the GLA (Fig. 2p and Fig. 2q), the total leaf image without impurities (Fig. 2o) is also obtained as a mask for the subsequent extraction of the GLA, and the binary images of green leaves without impurities are shown in Fig. 2r, s; and (vi) with a similar procedure and predefined segmentation values, green-2 leaf area, green-3 leaf area, and green-4 leaf area are also extracted by the set intersection of the corresponding segmented regions with impurities and the mask. When the single task is finished (such that the total leaves of an individual plant have all been measured with the HLS), the leaf area is determined by the sum of the pixel numbers for the current task and the spatial resolution of the inspection system (0.048mm^2^/pixel). The source code for the primary image analysis procedure is introduced in Supplementary Notes.

### Desktop scanner method

After the leaves of an individual rice plant were measured with the HLS, a DS (BenQ 8800, BenQ Corporation, China) was used to obtain the actual GLA and TLA (Caldas *et al.*, 1992). The clipped leaves were pasted on white sheets of paper, and then the papers were placed in the scanner. The scanner was set at 300 dpi to obtain 8-bit colour images. Using a fixed HSL colour threshold for green leaves (H: 39–131; S: 34–255; L: 30–233) and yellow leaves (H: 0–38; S: 34–255; L: 30–233), green leaves and yellow leaves were extracted from the background. Based on the foreground pixel counts of all the leaves that belong to the same plant, the GLA and TLA for individual rice plants were obtained.

### Genome-wide association study

A total of 4,358,600, 2,863,169, and 1,959,460 single nucleotide polymorphisms (SNPs; minor allele frequency ≥0.05; the number of accessions with minor alleles ≥6) were used in the GWAS for 29 traits at three different growth stages in the whole population and in indica and japonica sub-populations, respectively. The detailed method of GWAS is described in a previous study (Yang *et al.*, 2014). Suggestive (1/N) and significant (0.05/N) *P*-value thresholds were set to control the genome-wide type 1 error rate (Duggal *et al.*, 2008; Li *et al.*, 2013), and N represented the effective number of independent markers calculated using the GEC software tool (Li *et al.*, 2012). The software TASSEL version 3 (Bradbury *et al.*, 2007) was used to calculate phenotypic variation explained by individual SNPs after fitting other model terms in a mixed linear model, effect size of individual SNPs, and heritability [defined as the proportion of variance of the random effect (polygene) over total in mixed linear model]. Epistasis tests of pairwise SNPs were conducted with the function–epistasis in PLINK (http://pngu.mgh.harvard.edu/purcell/plink/) (Purcell *et al.*, 2007), and only tests with *P* < 10^−4^ were shown. Phenotypic variation explained by multiple significant SNPs was calculated by stepwise regression after coding one allele as 1 and the other as 0 (Li *et al.*, 2012). Candidate association analysis was also performed with TASSEL; the genotype data of the *Nal1* genomic region including *Nal1* itself and the upstream 2kb was extracted from RiceVarMap (http://ricevarmap.ncpgr.cn) (Zhao *et al.*, 2014).

## Results

### Performance evaluation of the HLS system

#### Performance evaluation of TLA and LN measurements

**Fig. 3.**
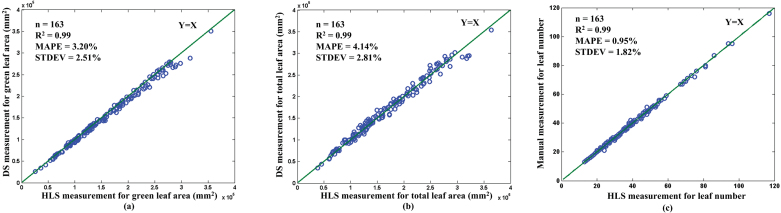
**The performance evaluation of the LN and leaf area extraction.** The scatter plots of the HLS measurement versus the DS measurement for the **(a)** GLA, **(b)** TLA, and **(c)** LN.

To test the HLS performance, 163 rice accessions from germplasm resources were randomly selected to measure the GLA, TLA, and LN. After the leaves of an individual rice plant were measured with the HLS, a DS (BenQ 8800) was used to obtain the actual GLA and TLA (Caldas *et al.*, 1992), and the LNs were counted manually. A comparison between the DS measurements and the HLS measurements for GLA are illustrated in Fig. 3a and TLA in Fig. 3b. The manual measurement versus the HLS measurement for LN is shown in Fig. 3c. The mean absolute per cent errors (MAPE, defined by Eq. 1) were 3.20% for GLA, 4.14% for TLA, and 0.95% for LN. The standard deviation of the absolute per cent error values for GLA was 2.51%, for TLA was 2.81%, and for LN was 1.82%. The coefficients of determination (R^2^) of the DS (or manual) measurements versus HLS measurements for GLA, TLA, and LN were all 0.99.

$$\mathrm{MA}PE=\frac{1}{n}\sum_{i=1}^{n}\frac{\left|x_{i.DS}-x_{i.HLS}\right|}{x_{i.DS}}$$ (1)

where *n* is the number of samples, *x*_*i.DS*_ is the *i*_*th*_ measured value using the DS method or manual method, and *x*_*i.HLS*_ is the *i*_*th*_ measured value using the HLS system.

### Performance evaluation of leaf colour discrimination

**Fig. 4.**
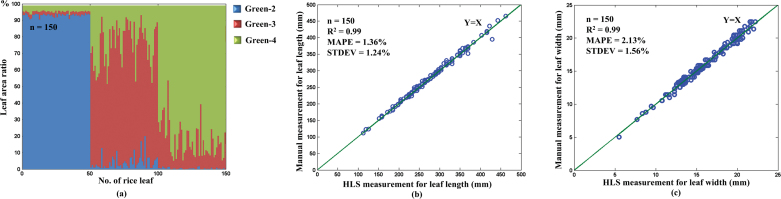
**The performance evaluation of the leaf colour and leaf shape extraction. (a**) The green colour ratio distribution of each batch (50 rice leaves) belonging to group 2 (light green), group 3 (green), and group 4 (dark green). The scatter plots of the HLS measurement versus the DS measurement for the **(b)** LL and **(c)** LW.

To evaluate the green leaf colour discrimination of the HLS, three batches of rice leaves (50 leaves for each batch) that belong to green-2 (light green), green-3 (green), and green-4 (dark green) were manually selected using the leaf colour chart developed by IRRI. Afterwards, each batch was measured using the HLS, and the results are shown in Fig. 4a and Supplementary Fig. S4. The discrimination errors for green-2, green-3, and green-4 were 0%, 0%, and 2%, respectively, demonstrating that the HLS is highly accurate in discriminating green leaf colours in rice.

### Performance evaluation for LL and LW

To evaluate the measuring accuracy of the HLS for LL and LW, the same three batches of rice leaves used for leaf greenness discrimination were measured using HLS and a Vernier caliper (manual method). The results are shown in Fig. 4b, c. The MAPE values were 1.36% for LL and 2.13% for LW, and the standard deviation of the absolute per cent error values were 1.24% for LL and 1.56% for LW.

### Change of the GLA at different growth stages

**Fig. 5.**
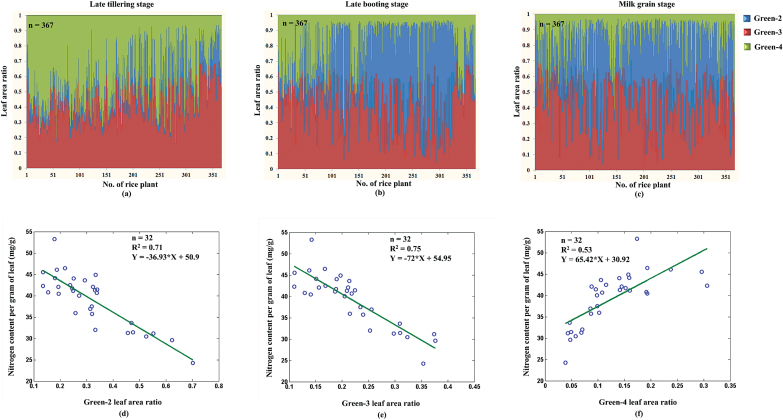
**The change in green leaf colour during the three growth stages.** The distribution of the GLA ratio at three growth stages as follows: **(a)** late tillering stage, **(b)** late booting stage, and **(c)** milk grain stage. The relationship between the nitrogen content and the GLA ratio: **(d)** green-2 leaf area ratio, **(e)** green-3 leaf area ratio, and **(f)** green-4 leaf area ratio.

The GLA shows visible change during the plant life cycle and in response to environmental cues, but the change pattern has seldom been systematically investigated because of measurement difficulties in traditional methods. By taking advantage of HLS, 367 rice accessions at three growth stages (late tillering stage, late booting stage, and milk grain stage) were chosen to analyse the change in the GLA (Fig. 5a–c). The leaf area ratio of the green-3 leaves changed little among the three growth stages, whereas the green-2 leaf area ratio became larger and the green-4 leaf area ratio became smaller with the growth and development of the rice plant, particularly from the late tillering stage to the late booting stage. The ratios of the three classes of green leaves are known to be related to the nitrogen content of the leaves. To investigate the relationship between the leaf nitrogen content and different green leaf colours, 32 rice accessions were randomly chosen and tested with HLS and with auto discrete analyzers (Smartchem200; Alliance Corporation, France). It was found that the green-2 and green-3 leaf area ratio were negatively correlated with the nitrogen content in leaves, and the green-4 leaf area ratio was positively correlated with the nitrogen content in leaves (Fig. 5d–f).

### GWAS of leaf traits

**Fig. 6.**
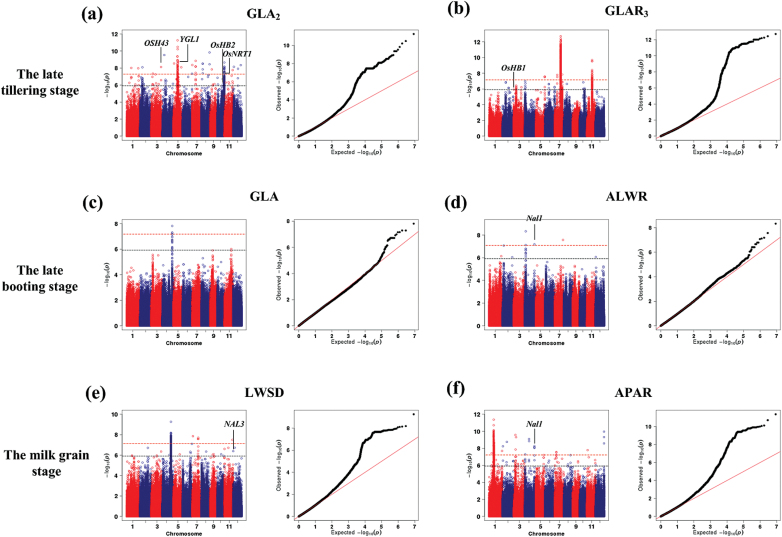
**Genome-wide association studies of six leaf traits.** Manhattan plots (left) and quantile-quantile plots (right) for **(a)** GLA_2_ and **(b)** GLAR_3_ at late tillering stage; **(c)** GLA and **(d)** ALWR at late booting stage; **(e)** LWSD and **(f)** APAR at milk grain stage. For Manhattan plots, -log10 *P*-values from a genome-wide scan are plotted against the position of the SNPs on each of 12 chromosomes and the horizontal grey dashed line indicates the genome-wide suggestive threshold (*P* = 1.21×10^–6^). The red dashed line indicates the genome-wide significant threshold (*P* = 6.03×10^–8^). For quantile-quantile plots, the horizontal axis shows the -log10-transformed expected *P*-values, and the vertical axis indicates -log10-transformed observed *P*-values. The names of known related genes near the association signals are shown.

**Fig. 7.**
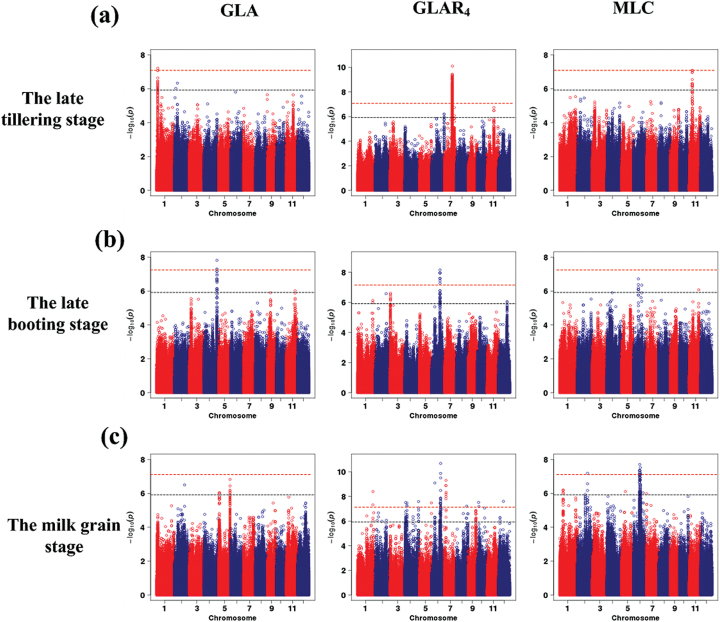
**Comparison of GWAS results for GLA, GLAR**_**4**_ **, and maximum leaf compactness at three different growth stages.** Manhattan plots of GLA related to the leaf area (left), GLAR_4_ related to the leaf colour (middle), and maximum leaf compactness (MLC) related to the leaf shape (right) at **(a)** late tillering stage, **(b)** late booting stage, and **(c)** milk grain stage. The horizontal grey dashed line and red dashed line indicates the genome-wide suggestive threshold (*P* = 1.21×10^–6^) and significant threshold (*P* = 6.03×10^–8^), respectively.

Using the HLS system, a total of 29 leaf traits were extracted from 533 rice accessions that have been re-sequenced for GWAS (Chen *et al.*, 2014). These leaf traits can be classified into three groups as follows: 6 leaf size–related traits, 7 leaf colour–related traits, and 16 leaf shape–related traits (Supplementary Table S1). GWAS was performed on these traits at three growth stages (late tillering stage, late booting stage, and milk grain stage). Using a Bonferroni correction based on the effective numbers of independent markers (Li *et al.*, 2012), the *P*-value thresholds were set at 1.21 × 10^−6^ (suggestive) and 6.03 × 10^−8^ (significant) (Supplementary Table S2). In this study, a total of 542 associations (462 lead SNPs) exceeding the suggestive threshold were identified (suggestive SNPs), of which 104 associations (96 significant SNPs) exceeded the significant threshold. For the convenience of presenting of the GWAS results, a chromosomal region in which the distance of adjacent pairs of associated SNPs was less than 300kb was defined as a locus (Chen *et al.*, 2014). As a result, 291 loci containing 462 suggestive SNPs (suggestive loci), and 78 loci containing 96 significant SNPs (significant loci) were detected (Fig. 6 and Supplementary Tables S3 and S4). There were some loci with multiple associated SNPs, and loci were repeatedly detected for different traits or repeatedly detected by the same trait at different growth stages. For example, locus 158 was detected with green-4 leaf area ratio (GLAR_4_) at late booting stage and milk grain stage, green-2 leaf area (GLA_2_) at late booting stage, and maximum leaf area at milk grain stage, simultaneously. Furthermore, two independently associated SNPs (LD statistics *r* ^*2*^ = 0.09) with GLAR_4_ at late booting stage, sf0620856382 and sf0620860929, were located at this locus at only a 4-kb distance. Among the 78 significant loci, 16 loci were repeatedly detected through different leaf traits; one locus was detected through the same trait at different growth stages; and two loci with multiple associated SNPs were detected (Supplementary Table S4). Manhattan plots for all the traits mentioned above are shown in Supplementary Fig. S5–S7. In total, 131, 62, and 173 suggestive loci and 37, 7, and 38 significant loci were detected at late tillering stage, late booting stage, and milk grain stage, respectively. Only five suggestive loci but no significant loci were detected at the three growth stages simultaneously. These results suggest that the genetic basis of these leaf traits at different growth stages may not be the same in rice (Fig. 7).

After adopting the suggestive *P*-value threshold, a total of nine loci associated with leaf traits contained leaf-related genes that were previously identified (Supplementary Table S3, Supplementary Note). For leaf colour-related traits, the lead SNPs at loci 81, 130, 238, and 289 that were associated with GLA_2_ at late tillering stage are close to *OsH43* (a member of the rice *kn1*-type class 1 homeobox gene family), *YGL1* (a leaf-colour related gene), *OsHB2* (a member of the rice Class III homeodomain leucine zipper gene family related to the establishment of polarity in developing leaves), and *Oshox1* (a member of the rice homeodomain leucine zipper gene family affecting the leaf size and shape), respectively (Itoh *et al.*, 2008; Meijer *et al.*, 1997; Sentoku *et al.*, 2000; Wu *et al.*, 2007).

For leaf shape-related traits, the lead SNPs at loci 81 and 266 that were associated with leaf width standard deviation (LWSD) per plant at milk grain stage are close to *OsH43* and *NAL3* (a rice WUSCHEL-related homeobox gene affecting rice LW), respectively (Cho *et al.*, 2013; Sentoku *et al.*, 2000). Lead SNPs at locus 109 were associated with LWSD, average leaf length to leaf width ratio (ALWR), average leaf perimeter to leaf area ratio (APAR) at late booting stage, and APAR, maximum LL, and maximum leaf perimeter to leaf area ratio (MPAR) at milk grain stage are close to *NAL1*, which affects the rice leaf blade width (Qi *et al.*, 2008). Lead SNPs at locus 45 (associated with average LL at late booting stage) and locus 49 (associated with average leaf perimeter at late booting stage) are close to *OsIAA3* in controlling the rice LL (Nakamura *et al.*, 2006).

Moreover, rice nitrate transporter gene *OsNRT1* (Lin *et al.*, 2000) was detected at locus 240, which was associated with GLA_2_ (a leaf colour related trait) at late tillering stage, which in turn partially supports the correlation between leaf colour and leaf nitrogen content mentioned above. In addition, a large number of new loci were identified. After applying the suggestive *P*-value threshold, a total of 17, 11, and 47 new loci were detected for six leaf size–related traits at late tillering stage, late booting stage, and milk grain stage; a total of 74, 13, and 48 new loci were identified for seven leaf colour–related traits; and a total of 48, 45, and 105 new loci were associated with sixteen leaf shape–related traits at the three stages. For example, new locus 180 (the *P*-value of a lead SNP sf0719639267 at this locus was 1.94 × 10^−13^) was repeatedly detected for GLAR_3_, GLAR_4_, green leaf colour classification, LWSD, and MPAR at late tillering stage, average leaf compactness at the late booting stage, and LWSD at the milk grain stage. All the potential candidate genes within 100-kb upstream and downstream of this lead SNP are shown in Supplementary Table S5. These results suggest that the combination of the HLS and GWAS can provide a powerful tool to identify genetic loci associated with leaf traits in rice.

The lead SNP sf0431040266 was associated with ALWR at the late booting stage (*P =* 6.59 × 10^−8^). In this locus, *Nal1*, a gene encoding an unknown protein with pleiotropic function including LW and photosynthesis rate (Qi *et al.*, 2008; Takai *et al.*, 2013), is only 11kb away from the lead SNP. The information supported that *Nal1* was at least partially responsible for the association signal. Candidate association analysis was conducted to demonstrate the association between *Nal1* and ALWR and to detect the causal variant. The genotypes of 64 SNPs were extracted and 23 SNPs remained for the following analysis after excluding SNPs with minor allele frequency <0.05 (Supplementary Table S6). Only sf0431027691 (*P* = 3.77 × 10^−4^), located in the third exon with non-synonymous mutation (transition from A to G), surpassed the Bonferroni-adjusted *P*-value (0.05/total markers = 2.17 × 10^−3^) in a mixed linear model, while the smallest *P*-value of other SNPs was 3.86 × 10^−2^. This indicates that the SNP was the possible causal variant for this trait. The results indicate that combining GWAS and HLS would be a promising approach to uncover the genetic basis of leaf traits and could provide sufficient resolution to detect causal variants with candidate association analysis.

## Discussion

### Measurement efficiency of the HLS

The HLS was designed for the high-throughput measurement of leaf traits, especially for addressing a large population with hundreds and even thousands of plants in a short time. In this study, with two workers feeding the rice leaves into the machine, the HLS requires approximately 5 minutes to process each rice plant (with approximately 100 leaves). With two similar workers, the DS measurement required a total of approximately 21 minutes per rice plant, including 10 minutes for leaf pasting, 10 minutes for leaf paper scanning, and 1 minute for image analysis. Thus, the efficiency of HLS is quadruple the efficiency of the DS method. Additionally, the pasted leaves from the DS method cannot be used for further measurements, such as measuring the fresh or dry weight. When continuously running, the HLS can measure up to 30 leaves per minute.

The pot-grown rice plants were cut up and then directly fed into the HLS. The time-consumption of leaf harvest was about 1–2 minutes depending on the proficiency of the two workers. Thus, taking the time-cost of leaf-harvesting into account, the total screen time for 100 leaves is approximately 6–7 minutes. The throughput of the HLS will be higher with more workers feeding in leaves. When provided with enough labour, increasing the FOV of the camera and the transverse feeding range can further improve the efficiency of HLS; however, increasing the FOV can also decrease the resolution of the inspection system. Thus, there is a trade-off between the measurement efficiency and measurement accuracy of the HLS. When measuring other species with fewer leaves (such as maize), the efficiency of the HLS will be significantly higher. Moreover, to make the HLS fit the different thicknesses of other plant leaves, the feeding interval between the cylinder and conveyor can be easily altered by adjusting a screw (Fig. 1c). Therefore, the system could be applied to other plants, such as maize, barley, and wheat.

### Relationships between leaf traits

By using the HLS, it was possible to extract multiple traits related to the leaf area, shape, and size simultaneously from a large population. The resulting large datasets allowed an analysis of the potential relationships between different types of leaf traits, which have seldom been addressed previously, primarily because of limitations in traditional leaf-phenotyping methods. From the leaf traits of 367 rice accessions, the relationship between the average leaf length × average leaf width × leaf number (ALWN) and the GLA per plant at three growth stages was compared (Supplementary Fig. S8a–c). The coefficients of determination (R^2^) were 0.93, 0.98, and 0.96, indirectly supporting the reliability of the four trait extractions with the HLS. We also compared the relationship between the maximum leaf length × maximum leaf width × leaf number (MLWN) and the GLA per plant at three growth stages (Supplementary Fig. S8d–f). The R^2^ at the three different growth stages (0.83, 0.82, 0.71) showed that the correlation between MLWNs and the GLA were lower than the correlation between ALWN and the GLA. A comparison of the scatter plots demonstrated that the rice leaves of one plant varied in shape. An advantage, therefore, of the HLS is that the GLA can be predicted with sampling, and that the average LW, LL, and LN can be calculated rather than only calculating these traits in the maximum leaf.

With the resulting leaf traits of 367 rice accessions, the APAR was found to be inversely related to the average leaf width during the three growth stages. Because the rice leaf shape is close to a rectangle, APAR could be calculated as indicated by equation 2.

$$APAR=\frac{ALP}{ALA}=\frac{2\times ALL+2\times ALW}{ALL\times ALW}=\frac{2}{ALW}+\frac{2}{ALL}$$ (2)

ALP, ALA, ALL, and ALW are the average leaf perimeter, average leaf area, average leaf length, and average leaf width, respectively (as listed in Supplementary Table S1). Because the value of ALL is much greater than that of ALW, equation 2 can be reduced to equation 3, which is consistent with the relationship in Supplementary Fig. S9a–c, also demonstrating the reliability of the leaf shape trait extraction by the HLS system.

$$APAR=\frac{2}{ALW}$$ (3)

### Genetic heterogeneity across different sub-populations

Genetic heterogeneity across different sub-populations is a common phenomenon in rice (Huang *et al.*, 2011; Zhao *et al.*, 2011), and thus a GWAS of the 29 leaf traits in three growth stages of the indica and japonica sub-populations were also performed. In comparison with the GWAS results in the whole population, new association signals were identified in the indica or japonica sub-population for MPAR at late booting stage and ALWR and MPAR at milk grain stage (Supplementary Fig. S10). For MPAR at milk grain stage, no clear associations were detected in the whole population, but three strong peak-like association signals appeared in the indica sub-population. These results suggest genetic heterogeneity could reduce the power to detect associations in the whole population, and thus it is necessary to perform a GWAS in different sub-populations separately to identify loci associated with leaf traits.

### Genetic architecture of different leaf traits

A difference in genetic architecture was observed for different leaf-related traits. For example, genetic variance explained by a total of 24, 28, and 13 suggestive lead SNPs was 64%, 59%, and 41% respectively for GLAR_4_ (leaf-colour related), ALWR (leaf-shape related), and ALA (leaf-size related) at milk grain stage (Supplementary Tables S7 and S8). The remaining genetic variance may be explained by the SNPs with small effect that failed to surpass genome-wide *P*-value thresholds. A higher genotype–environment interaction could contribute to low heritability of GLAR_4_ (*h* ^*2*^= 0.54). For GLA_2_ at late tillering stage, 55 suggestive lead SNPs explained up to 80% of phenotypic variance, suggesting the leading role of additive effect in GLA_2_. In addition to additive effect detected by GWAS, significant epistasis interactions of pairwise lead SNPs (*P* value < 10^−4^) were also discovered in this study (Supplementary Table S9). For each trait, different associated variants had different effect size with either positive or negative effect. Also for GLA_2_, 7 out of 22 significant lead SNPs had negative effects and the others had positive effects relative to the reference allele (Supplementary Table S4).

To promote the development of leaf phenomics and the dissection of leaf genetic architecture (Micol, 2009), this paper describes an engineering prototype HLS for high-throughput leaf trait phenotyping that can extract 29 leaf traits from rice. In addition to the traditionally defined leaf traits (such as LL, LW, and leaf area), many newly defined leaf traits were extracted by the HLS. By combining a GWAS with HLS, a large number of genetically associated loci that could not be detected by the previous leaf phenotyping method and/or mapping approaches were identified for both the traditional and newly defined leaf traits in this study. The HLS system was originally designed for use in the greenhouse. If portable power is provided, an HLS system with wheels can be used in the field. In summary, by using multidisciplinary technologies, particularly a photonics-based technique (Yang *et al.*, 2013), a combination of the HLS and GWAS has provided valuable insights into the genetic basis of rice leaf traits.

## Supplemental Data

Supplementary Fig. S1. The experimental arrangement of the rice material.

Supplementary Fig. S2. The operational procedure of the HLS system.

Supplementary Fig. S3. The data management of the HLS system.

Supplementary Fig. S4. The HLS measurements for the rice leaf colour of each batch (50 leaves) belonging to (a) group 2, (b) group 3, and (c) group 4.

Supplementary Fig. S5. Genome-wide association studies of six (leaf size–related) traits at three different growth stages.

Supplementary Fig. S6. Genome-wide association studies of seven (leaf colour–related) traits at three different growth stages.

Supplementary Fig. S7. Genome-wide association studies of 16 (leaf shape–related) traits at three different growth stages.

Supplementary Fig. S8. Scatter plots of leaf shape measurements versus GLA measurements at three growth stages.

Supplementary Fig. S9. Scatter plots of average leaf width measurements versus average leaf perimeter area ratio measurements at three growth stages.

Supplementary Fig. S10. GWAS results for three leaf traits in three different (sub)populations.

Supplementary Table S1. Leaf traits calculations as performed with the HLS.

Supplementary Table S2. The effective number of SNPs across the rice genome and linkage disequilibrium–adjusted Bonferroni-corrected *P*-value thresholds.

Supplementary Table S3. GWAS results with the suggestive *P*-value threshold.

Supplementary Table S4. GWAS results with the significant *P*-value threshold.

Supplementary Table S5. All the potential candidate genes within 100-kb upstream and downstream of the lead SNP sf0719639267.

Supplementary Table S6. Association analysis of *Nal1* with ALWR at the late booting stage.

Supplementary Table S7. Variance explained by suggestive lead SNPs.

Supplementary Table S8. The heritability (*h* ^*2*^) of 29 leaf traits at three growth stages.

Supplementary Table S9. Epistasis analysis of significant lead SNPs.

Supplementary Video S1. The procedure of the HLS system.

Supplementary Notes. (1) The source code and annotation of image acquisition; (2) The source code and annotation of image correction; (3) The source code and annotation of image processing; (4) the predefined segmentation values in HSL colour space; (5) the hardware control protocol and the source code of PLC control; (6) Associated lead SNPs linked to known genes.

## Supplementary Material

Supplementary Data

## Abbreviations:

ALWN: average leaf length × average leaf width × leaf number
ALWR: average leaf length to leaf width ratio
APAR: average leaf perimeter to leaf area ratio
DS: , desktop scanner
FOV: field of view
GLA: green leaf area
GLAR: green leaf area ratio
GWAS: genome-wide association study
HLS: high-throughput leaf scoring
HSL: hue, saturation, and illumination
IRRI: International Rice Research Institute
LAI: leaf area index
LL: leaf length
LN: leaf number
LW: leaf width
MAPE: mean absolute per cent errors
MPAR: maximum leaf perimeter to leaf area ratio
QTL: quantitative trait loci
RGB: red, green, blue
SNP: single nucleotide polymorphism
TLA: total leaf area.

## References

[CIT0001] Bakr EM 2005 A new software for measuring leaf area, and area damaged by *Tetranychus urticae* Koch. Journal of Applied Entomology 129, 173–175.

[CIT0002] Bradbury PJ Zhang Z Kroon DE Casstevens TM Ramdoss Y Buckler ES 2007 TASSEL: software for association mapping of complex traits in diverse samples. Bioinformatics 23, 2633–2635. 17586829 10.1093/bioinformatics/btm308

[CIT0003] Bylesjö M Segura V Soolanayakanahally RY Rae AM Trygg J Gustafsson P Jansson S Street NR 2008 LAMINA: a tool for rapid quantification of leaf size and shape parameters. BMC Plant Biology 8, 82. 18647399 10.1186/1471-2229-8-82 PMC2500018

[CIT0004] Byrne ME 2005 Networks in leaf development. Current Opinion in Plant Biology 8, 59–66. 15653401 10.1016/j.pbi.2004.11.009

[CIT0005] Caldas LS Bravo C Piccilo H Faria CRSM 1992 Measurement of leaf area with a hand-scanner linked to a microcomputer. Revista Brasileira de Fisiologia Vegetal 4, 17–20.

[CIT0006] Chen W Gao Y Xie W Gong L Lu K Wang W Li Y Liu X Zhang H Dong H 2014 Genome-wide association analyses provide genetic and biochemical insights into natural variation in rice metabolism. Nature Genetics 46, 714–721. 24908251 10.1038/ng.3007

[CIT0007] Cho SH Yoo SC Zhang H Pandeya D Koh HJ Hwang JY Kim GT Paek NC 2013 The rice narrow leaf 2 and narrow leaf 3 loci encode WUSCHEL-related homeobox 3A (OsWOX3A) and function in leaf, spikelet, tiller and lateral root development. New Phytologist 198, 1071–1084. 23551229 10.1111/nph.12231

[CIT0008] Deng X Zhang H Wang Y He F Liu J Xiao X Shu Z Li W Wang G Wang G 2014 Mapped clone and functional analysis of leaf-color gene *Ygl7* in a rice hybrid (*Oryza sativa* L. ssp. indica). PLOS One 9, e99564. 24932524 10.1371/journal.pone.0099564 PMC4059691

[CIT0009] Duggal P Gillanders EM Holmes TN Bailey-Wilson JE 2008 Establishing an adjusted p-value threshold to control the family-wide type 1 error in genome wide association studies. BMC Genomics 9, 516. 18976480 10.1186/1471-2164-9-516 PMC2621212

[CIT0010] Farooq M Tagle AG Santos RE Ebron LA Fujita D Kobayashi N 2010 Quantitative trait loci mapping for leaf length and leaf width in rice cv. IR64 derived lines. Journal of Integrative Plant Biology 52, 578–584. 20590988 10.1111/j.1744-7909.2010.00955.x

[CIT0011] Fujino K Matsuda Y Ozawa K Nishimura T Koshiba T Fraaije MW Sekiguchi H 2008 *NARROW LEAF 7* controls leaf shape mediated by auxin in rice. Molecular Genetics and Genomics 279, 499–507. 18293011 10.1007/s00438-008-0328-3

[CIT0012] Hu J Zhu L Zeng D Gao Z Guo L Fang Y Zhang G Dong G Yan M Liu J 2010 Identification and characterization of *NARROW AND ROLLED LEAF 1*, a novel gene regulating leaf morphology and plant architecture in rice. Plant Molecular Biology 73, 283–292. 20155303 10.1007/s11103-010-9614-7

[CIT0013] Huang X Zhao Y Wei X Li C Wang A Zhao Q Li W Guo Y Deng L Zhu C 2011 Genome-wide association study of flowering time and grain yield traits in a worldwide collection of rice germplasm. Nature Genetics 44, 32–39. 22138690 10.1038/ng.1018

[CIT0014] Itoh J Hibara K Sato Y Nagato Y 2008 Developmental role and auxin responsiveness of Class III homeodomain leucine zipper gene family members in rice. Plant Physiology 147, 1960–1975. 18567825 10.1104/pp.108.118679 PMC2492597

[CIT0015] Kessler S Sinha N 2004 Shaping up: the genetic control of leaf shape. Current Opinion in Plant Biology 7, 65–72. 14732443 10.1016/j.pbi.2003.11.002

[CIT0016] Li H Peng Z Yang X Wang W Fu J Wang J Han Y Chai Y Guo T Yang N 2013 Genome-wide association study dissects the genetic architecture of oil biosynthesis in maize kernels. Nature Genetics 45, 43–50. 23242369 10.1038/ng.2484

[CIT0017] Li M Yeung JM Cherny SS Sham PC 2012 Evaluating the effective numbers of independent tests and signiﬁcant p-value thresholds in commercial genotyping arrays and public imputation reference datasets. Human Genetics 131, 747–756. 22143225 10.1007/s00439-011-1118-2 PMC3325408

[CIT0018] Lin CM Koh S Stacey G Yu SM Lin TY Tsay YF 2000 Cloning and functional characterization of a constitutively expressed nitrate transporter gene, *OsNRT1*, from rice. Plant Physiology 122, 379–388. 10677431 10.1104/pp.122.2.379 PMC58875

[CIT0019] Meijer AH Scarpella E van Dijk EL Qin L Taal AJ Rueb S Harrington SE McCouch SR Schilperoort RA Hoge JH 1997 Transcriptional repression by Oshox1, a novel homeodomain leucine zipper protein from rice. The Plant Journal 11, 263–276. 9076993 10.1046/j.1365-313x.1997.11020263.x

[CIT0020] Micol JL 2009 Leaf development: time to turn over a new leaf? Current Opinion in Plant Biology 12, 9–16. 19109050 10.1016/j.pbi.2008.11.001

[CIT0021] Moon J Hake S 2011 How a leaf gets its shape. Current Opinion in Plant Biology 14, 24–30. 20870452 10.1016/j.pbi.2010.08.012

[CIT0022] Nakamura A Umemura I Gomi K Hasegawa Y Kitano H Sazuka T Matsuoka M 2006 Production and characterization of auxin-insensitive rice by overexpression of a mutagenized rice IAA protein. The Plant Journal 46, 297–306. 16623891 10.1111/j.1365-313X.2006.02693.x

[CIT0023] O’Neal ME Landis DA Isaacs R 2002 An inexpensive, accurate method for measuring leaf area and defoliation through digital image analysis. Journal of Economic Entomology 95, 1190–1194. 12539831 10.1603/0022-0493-95.6.1190

[CIT0024] Pérez-Pérez JM Esteve-Bruna D Micol JL 2010 QTL analysis of leaf architecture. Journal of Plant Research 123, 15–23. 19885640 10.1007/s10265-009-0267-z

[CIT0025] Purcell S Neale B Todd-Brown K Thomas L Ferreira MAR Bender D Maller J Sklar P Bakker PIW Daly MJ 2007 PLINK: a tool set for whole-genome association and population-based linkage analyses. American Journal of Human Genetics 81, 559–575. 17701901 10.1086/519795 PMC1950838

[CIT0026] Qi J Qian Q Bu Q Li S Chen Q Sun J Liang W Zhou Y Chu C Li X 2008 Mutation of the rice *Narrow leaf* 1 gene, which encodes a novel protein, affects vein patterning and polar auxin transport. Plant Physiology 147, 1947–1959. 18562767 10.1104/pp.108.118778 PMC2492643

[CIT0027] Sentoku N Sato Y Matsuoka M 2000 Overexpression of rice *OSH* genes induces ectopic shoots on leaf sheaths of transgenic rice plants. Developmental Biology 220, 358–364. 10753522 10.1006/dbio.2000.9624

[CIT0028] Shibayama M Sakamoto T Takada E Inoue A Morita K Takahashi W Kimura A 2011a Estimating paddy rice leaf area index with fixed point continuous observation of near infrared reflectance using a calibrated digital camera. Plant Production Science 14, 30–46.

[CIT0029] Shibayama M Sakamoto T Takada E Inoue A Morita K Yamaguchi T Takahashi W Kimura A 2011b Regression-based models to predict rice leaf area index using biennial fixed point continuous observations of near infrared digital images. Plant Production Science 14, 365–376.

[CIT0030] Singh B Singh Y Ladha JK Bronson KF Balasubramanian V Singh J Khind CS 2002 Chlorophyll meter– and leaf color chart–based nitrogen management for rice and wheat in northwestern India. Agronomy Journal 94, 821–829.

[CIT0031] Sone C Saito K Futakuchi K 2009 Comparison of three methods for estimating leaf area index of upland rice cultivars. Crop Science 49, 1438–1443.

[CIT0032] Takai T Adachi S Taguchi-Shiobara F Sanoh-Arai Y Iwasawa N Yoshinaga S Hirose S Taniguchi Y Yamanouchi U Wu J 2013 A natural variant of *NAL1*, selected in high-yield rice breeding programs, pleiotropically increases photosynthesis rate. Scientific Reports **3**, 2149. 10.1038/srep02149 PMC3756344 23985993

[CIT0033] Tian F Bradbury PJ Brown PJ Hung H Sun Q Flint-Garcia S Rocheford TR McMullen MD Holland JB Buckler ES 2011 Genome-wide association study of leaf architecture in the maize nested association mapping population. Nature Genetics 43, 159–164. 21217756 10.1038/ng.746

[CIT0034] Tsukaya H 2003 Organ shape and size: a lesson from studies of leaf morphogenesis. Current Opinion in Plant Biology 6, 57–62. 12495752 10.1016/s1369526602000055

[CIT0035] Wang F Huang J Lou Z 2011 A comparison of three methods for estimating leaf area index of paddy rice from optimal hyperspectral bands. Precision Agriculture 12, 439–447.

[CIT0036] Wang P Zhou G Yu H Yu S 2011 Fine mapping a major QTL for flag leaf size and yield–related traits in rice. Theoretical and Applied Genetics 123, 1319–1330. 21830109 10.1007/s00122-011-1669-6

[CIT0037] Wijk MTV Williams M 2005 Optical instruments for measuring leaf area index in low vegetation: application in arctic ecosystems. Ecological Applications 15, 1462–1470.

[CIT0038] Wu Z Zhang X He B Diao L Sheng S Wang J Guo X Su N Wang L Jiang L 2007 A chlorophyll-deficient rice mutant with impaired chlorophyllide esterification in chlorophyll biosynthesis. Plant Physiology 145, 29–40. 17535821 10.1104/pp.107.100321 PMC1976586

[CIT0039] Yang W Duan L Chen G Xiong L Liu Q 2013 Plant phenomics and high-throughput phenotyping: accelerating rice functional genomics using multidisciplinary technologies. Current Opinion in Plant Biology 16, 180–187. 23578473 10.1016/j.pbi.2013.03.005

[CIT0040] Yang W Guo Z Huang C Duan L Chen G Jiang N Fang W Feng H Xie W Lian X 2014 Combining high-throughput phenotyping and genome-wide association studies to reveal natural genetic variation in rice. Nature Communications 5, 5087. 10.1038/ncomms6087 PMC4214417 25295980

[CIT0041] Yue B Xue W Luo L Xing Y 2006 QTL analysis for flag leaf characteristics and their relationships with yield and yield traits in rice. Yi Chuan Xue Bao 33, 824–832. 16980129 10.1016/S0379-4172(06)60116-9

[CIT0042] Zhang Q 2007 Strategies for developing green super rice. Proceedings of the National Academy of Sciences U S A 104, 16402–16409. 10.1073/pnas.0708013104 PMC2034246 17923667

[CIT0043] Zhao H Yao W Ouyang Y Yang W Wang G Lian X Xing Y Chen L Xie W 2014 RiceVarMap: a comprehensive database of rice genomic variations. Nucleic Acids Research, 43, D1018–D1022. 25274737 10.1093/nar/gku894 PMC4384008

[CIT0044] Zhao K Tung CW Eizenga GC Wright MH Ali ML Price AH Norton GJ Islam MR Reynolds A Mezey J 2011 Genome-wide association mapping reveals a rich genetic architecture of complex traits in *Oryza sativa* . Nature Communications, 2, 467. 10.1038/ncomms1467 PMC3195253 21915109

