# Supplementary material for: Genome-wide association study of rice (Oryza sativa L.) leaf traits with a high-throughput leaf scorer
Source: J Exp Bot. 2015 Mar 20;66(18):5605–15. doi: 10.1093/jxb/erv100 (PMC4585412; doi:10.1093/jxb/erv100)
Supplement: Supplementary Data [file supp_66_18_5605__index.html]

Genome-wide association study of rice (Oryza sativa L.) leaf traits with a high-throughput leaf scorer — Genome-wide association study of rice (Oryza sativa L.) leaf traits with a high-throughput leaf scorer — Supplementary Data 

# Genome-wide association study of rice (*Oryza sativa* L.) leaf traits with a high-throughput leaf scorer

## Supplementary Data

Data files

**Files in this Data Supplement:**

- Supplementary Data - Supplementary Data
- Supplementary Data - Supplementary Data
