# Supplementary material for: Genome-wide association study of rice (Oryza sativa L.) leaf traits with a high-throughput leaf scorer
Source: J Exp Bot. 2015 Mar 20;66(18):5605–15. doi: 10.1093/jxb/erv100 (PMC4585412; doi:10.1093/jxb/erv100)
Supplement: Supplementary Data [file supp_erv100_jexbot136952_file001.pdf]

Supplementary figures list:

Supplementary Figure S1. The experimental arrangement of the rice material.

Supplementary Figure S2. The operational procedure of the HLS system.

Supplementary Figure S3. The data management of the HLS system.

Supplementary Figure S4. The HLS measurements for the rice leaf color of each batch (50 leaves) belonging to (a) group 2, (b) group 3, and (c) group 4.

Supplementary Figure S5. Genome-wide association studies of 6 (leaf size related) traits at three different growth stages.

Supplementary Figure S6. Genome-wide association studies of 7 (leaf color related) traits at three different growth stages.

Supplementary Figure S7. Genome-wide association studies of 16 (leaf shape-related) traits at three different growth stages.

Supplementary Figure S8. Scatter plots of leaf shape measurements versus green leaf area measurements at three growth stages.

Supplementary Figure S9. Scatter plots of average leaf width measurements versus average leaf perimeter area ratio measurements at three growth stages.

Supplementary Figure S10. GWAS results for three leaf traits in three different (sub)populations.

Supplementary Notes list:

1. Image acquisition

2. Image correcting

3. Image processing

4. The predefined segmentation values in HSL color space.

5. The hardware control protocol and the source code of PLC.

6. Associated lead SNPs of loci linked to known genes.

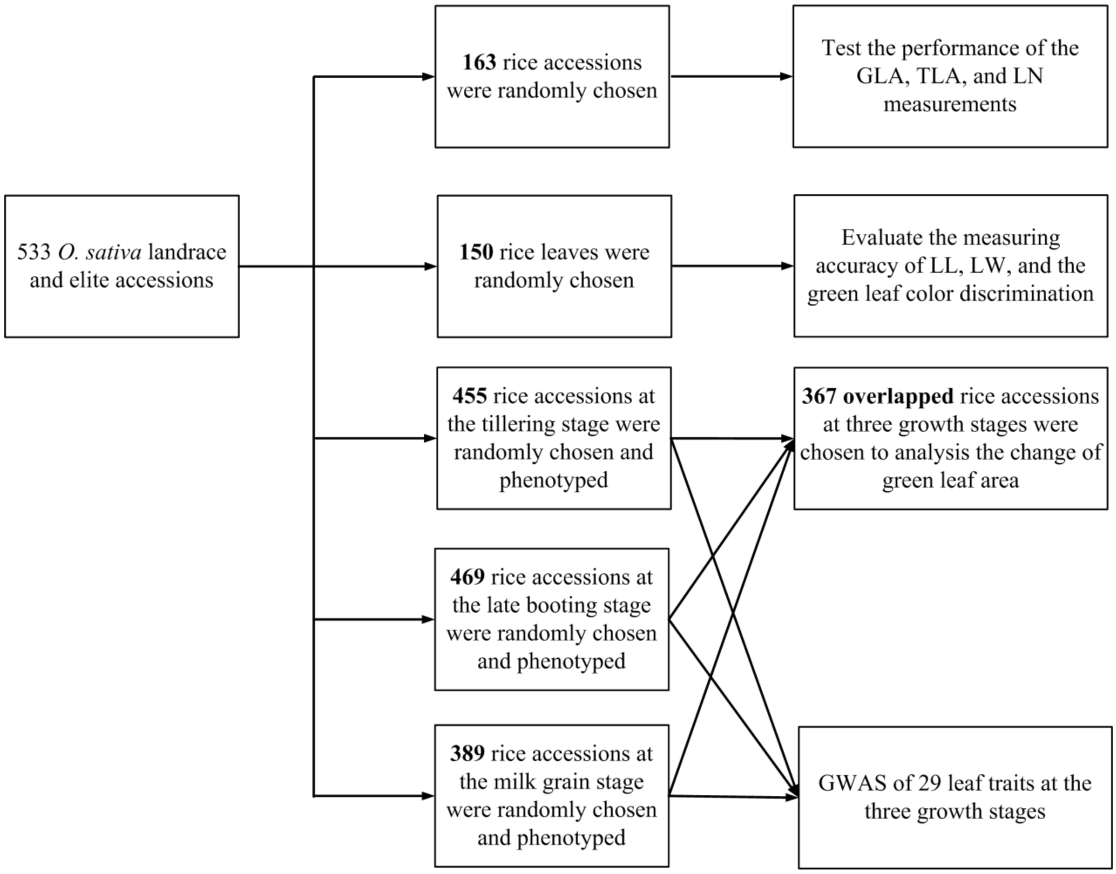

Supplementary Figure S1. The experimental arrangement of the rice material. The growth stages of the 533 accessions were not synchronous, and the inspection task was finished within one month at each growth stage, thus not all rice accessions were phenotyped with the HLS system. A total of 455, 469, and 389 rice accessions at late tillering stage, late booting stage, and milk grain stage, respectively, were phenotyped.

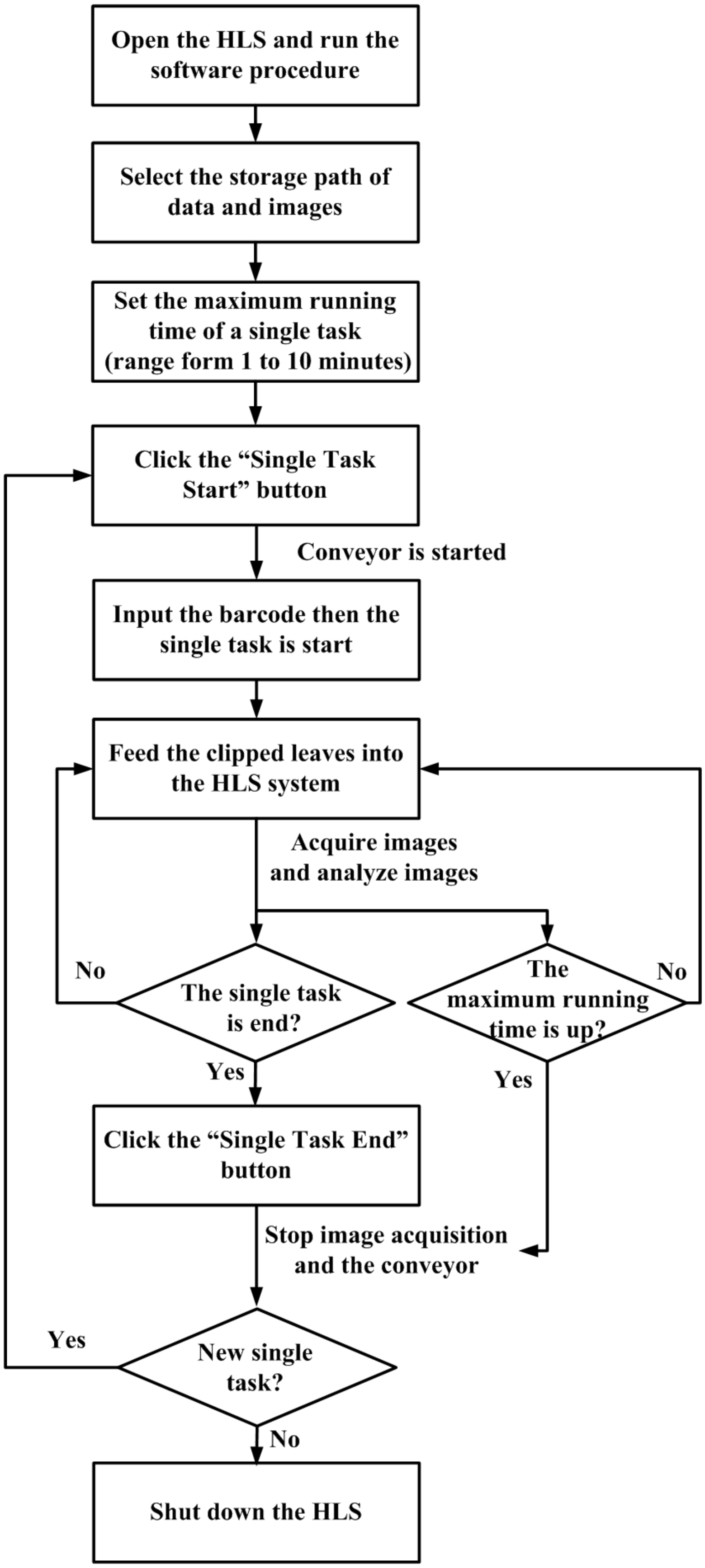

Supplementary Figure S2. The operational procedure of the HLS system includes the following

steps: (1) open the HLS and run the software procedure; (2) select the storage path for data and images; (3) set the maximum running time of a single task (the optional range is from 1 to 10 minutes; typically 5 minutes is suitable for an individual rice plant); (4) click the "Single Task Start" button on the right side of the HLS (Fig. 1b) to start the conveyor; (5) enter the barcode to begin the camera scan and transmit the images to the workstation; (6) feed in all the leaves for a single task using the feeding device; (7) after all the leaves are fed and scanned, click the “Single Task End” button on the software interface; and (8) when all the tasks are finished, click the "STOP" button on the software interface.

High-throughput Leaf Scorer

Barcode	Green Leaf Area (mm^2)	Yellow Leaf Area (mm^2)	Total Leaf Area (mm^2)	Green Leaf Area Ratio	Green-2 Leaf Area (mm^2)	Green-2 Leaf Area ratio	Green-3 Leaf Area (mm^2)	Green-3 Leaf Area ratio	Green-4 Leaf Area (mm^2)	Green-4 Leaf Area ratio
1	178194	33786	211980	84.06%	47209	26.49%	117667	66.03%	13318	7.47%
2	165333	664	165997	99.60%	219	0.13%	11833	7.16%	153281	92.71%
3	126872	32912	159783	79.40%	66477	52.40%	58779	46.33%	1615	1.27%
4	104874	6781	111656	93.93%	16601	15.83%	56141	53.53%	32133	30.64%
5	117940	15238	133178	88.56%	506	0.43%	63151	53.55%	54282	46.03%
6	184897	7039	191936	96.33%	2490	1.35%	92939	50.27%	89467	48.39%
7	83698	7637	91335	91.64%	22360	26.72%	55561	66.38%	5776	6.90%
8	66120	7039	73159	90.38%	602	0.91%	30700	46.43%	34818	52.66%
9	103554	1766	105319	98.32%	10711	10.34%	88810	85.76%	4032	3.89%
10	106295	955	107250	99.11%	494	0.46%	20420	19.21%	85381	80.32%
11	62174	8771	70944	87.64%	381	0.61%	33940	54.59%	27852	44.80%
12	178199	3258	181457	98.20%	5104	2.86%	45562	25.57%	127533	71.57%
13	232737	5471	238208	97.70%	9134	3.92%	148835	63.95%	74769	32.13%
14	101511	684	102195	99.33%	2249	2.22%	56958	56.11%	42304	41.67%
15	132435	419	132854	99.68%	210	0.16%	5909	4.46%	126316	95.38%
16	132017	5020	137037	96.34%	4059	3.07%	69465	52.62%	58493	44.31%
17	116566	4931	121497	95.94%	118	0.10%	16335	14.01%	100112	85.88%
18	149420	9523	158943	94.01%	24249	16.23%	97238	65.08%	27933	18.69%
19	92967	11161	104128	89.28%	7983	8.59%	62285	67.00%	22699	24.42%
20	119440	2200	121640	98.19%	727	0.61%	32234	26.99%	86479	72.40%
21	62388	2786	65174	95.73%	969	1.55%	36933	59.20%	24487	39.25%
22	233315	3221	236535	98.64%	16173	6.93%	136494	58.50%	80648	34.57%
23	277070	2163	279232	99.23%	564	0.20%	19765	7.13%	256741	92.66%
24	177821	5202	183023	97.16%	4010	2.26%	118963	66.90%	54847	30.84%
25	160731	7925	168656	95.30%	4818	3.00%	115361	71.77%	40551	25.23%

(a)

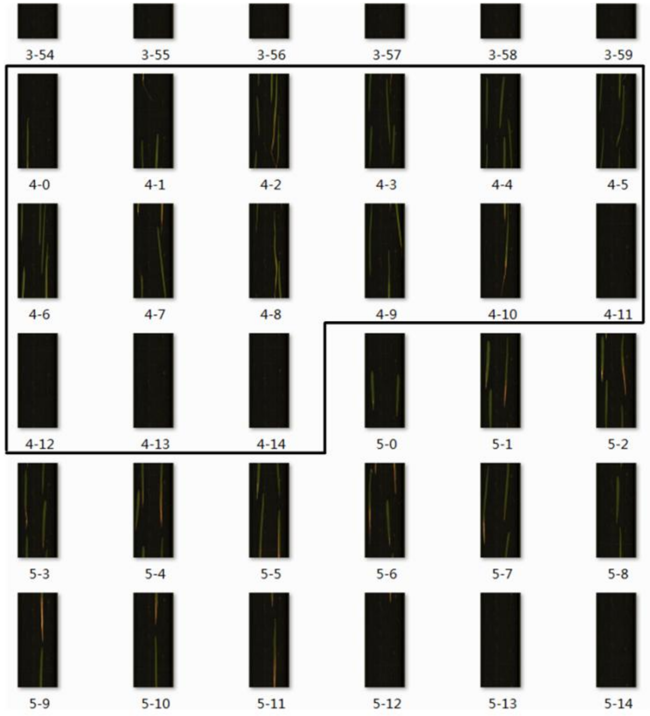

(b)

Supplementary Figure S3. The data management of the HLS system. (a) After the "Single Task End" button is clicked, the leaf traits (including barcode and whole leaf traits) are automatically stored in an Excel file. (b) Along with barcode indexing, the scanned images from each task are automatically stored in the user-predefined folder.

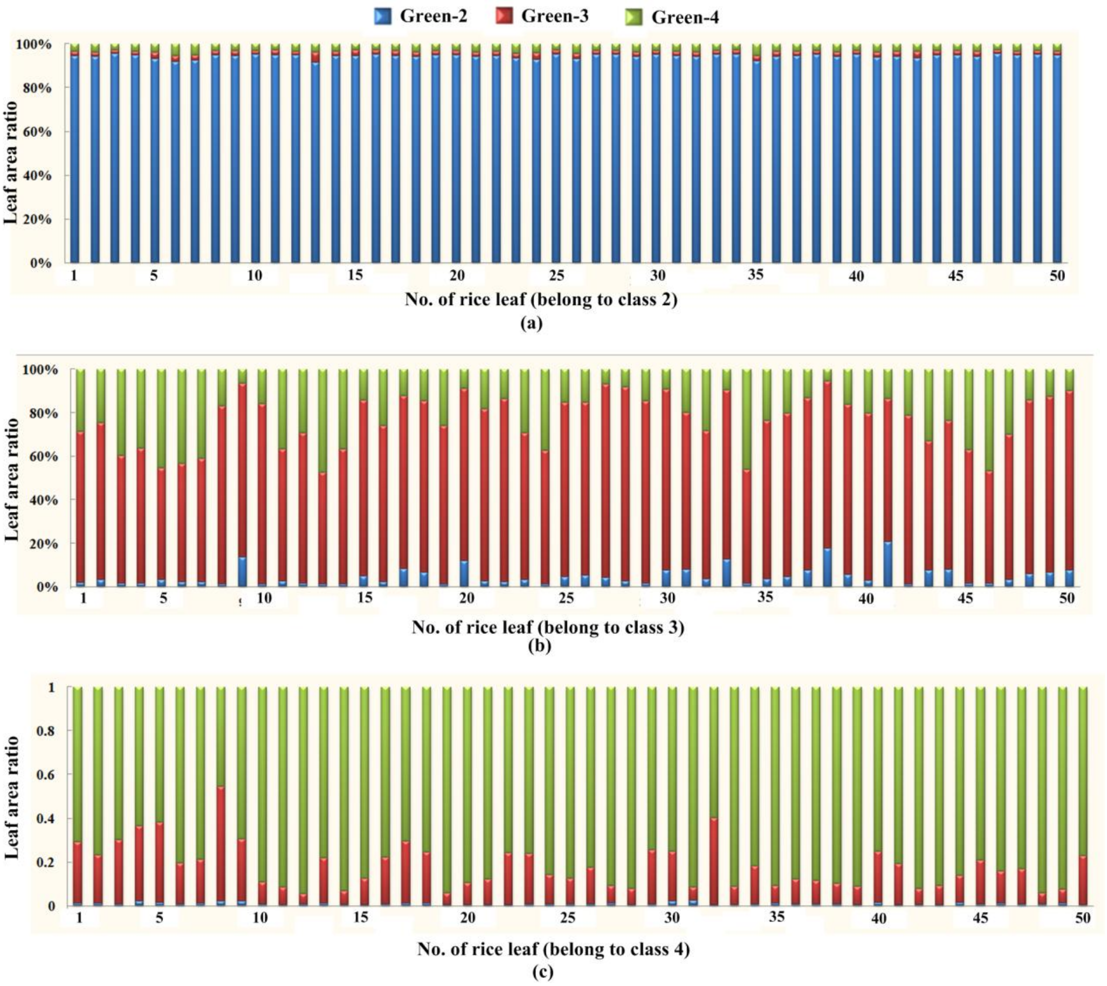

Supplementary Figure S4. The HLS measurements for the rice leaf color of each batch (50 leaves) belonging to (a) group 2, (b) group 3, and (c) group 4.

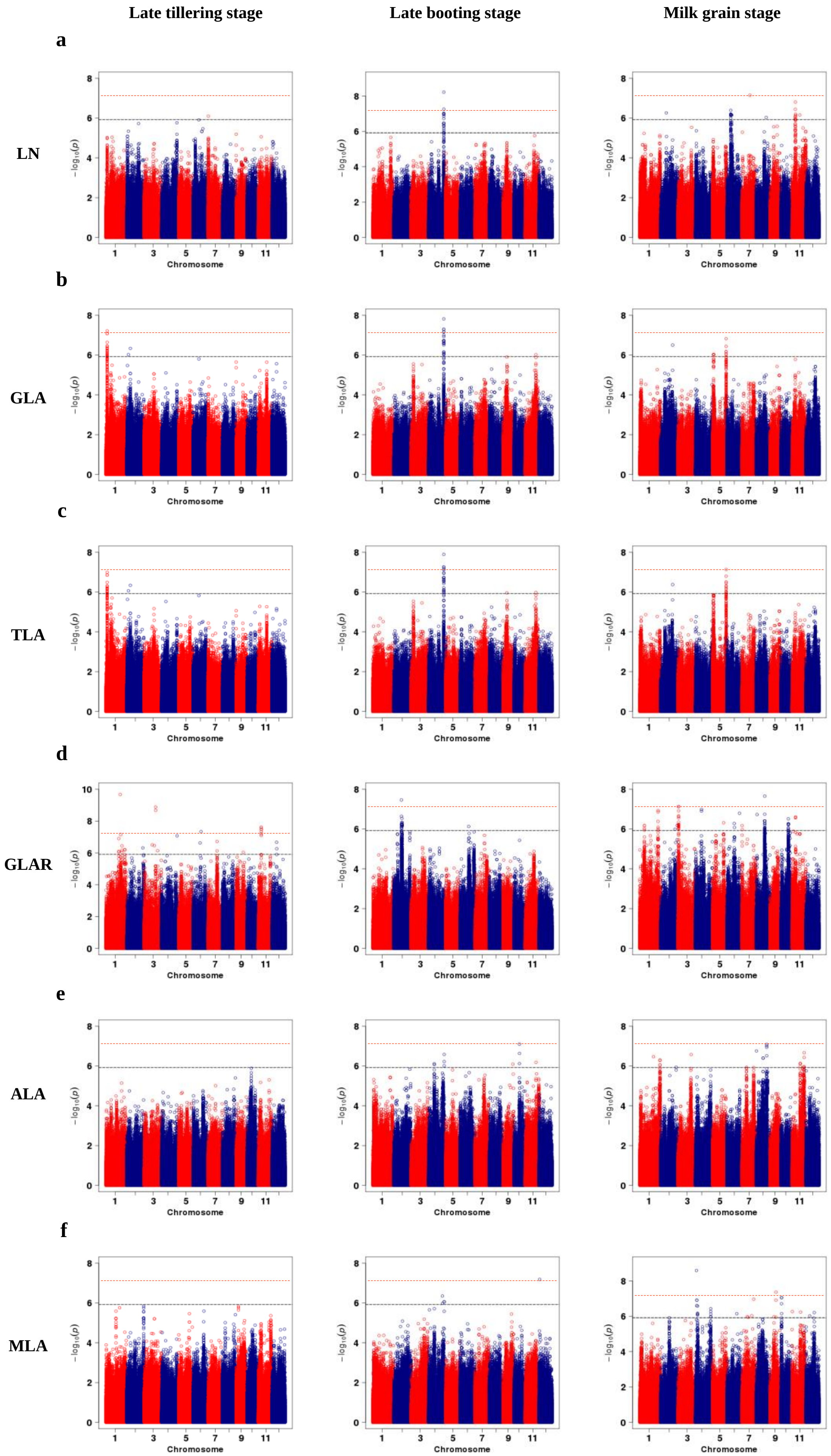

Late tillering stage
Late booting stage
Milk grain stage
a
LN
b
GLA
c
TLA
d
GLAR
e
ALA
f
MLA
Chromosome

Supplementary Figure S5. Genome-wide association studies of 6 (leaf size related) traits at three different growth stages. Manhattan plots of LN (a), GLA (b), TLA (c), GLAR (d), ALA (e) and MLA (f) at late tillering stage (left), late booting stage (middle), and milk grain stage (right), respectively. The horizontal gray dashed line and red dashed line indicates the genome-wide suggestive threshold ($P = 1.21\times10^{-6}$) and significant threshold ($P = 6.03\times10^{-8}$), respectively.

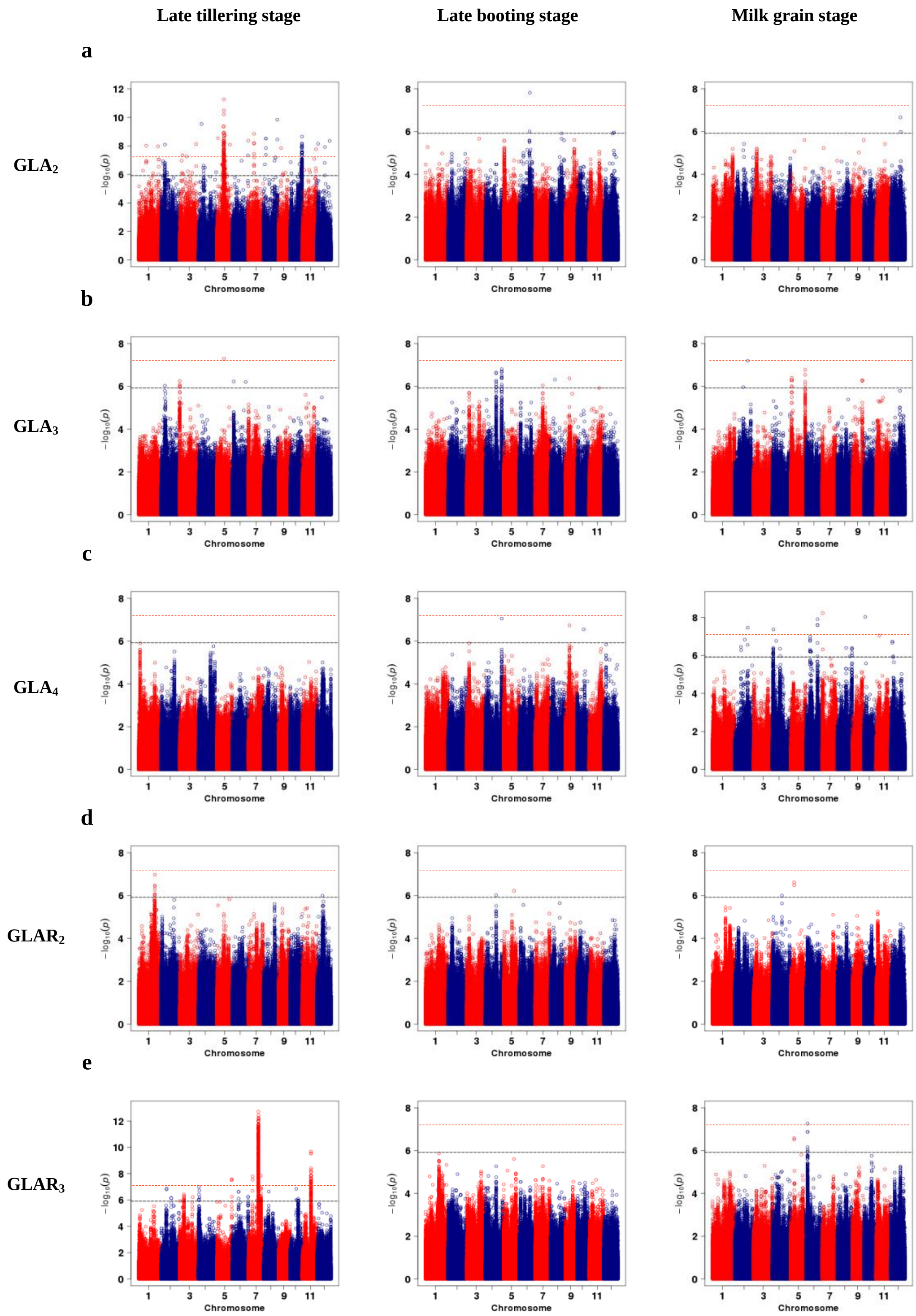

Late tillering stage
Late booting stage
Milk grain stage
a
b
c
d
e
GLA2
GLA3
GLA4
GLAR2
GLAR3
Chromosome
−log10(p)

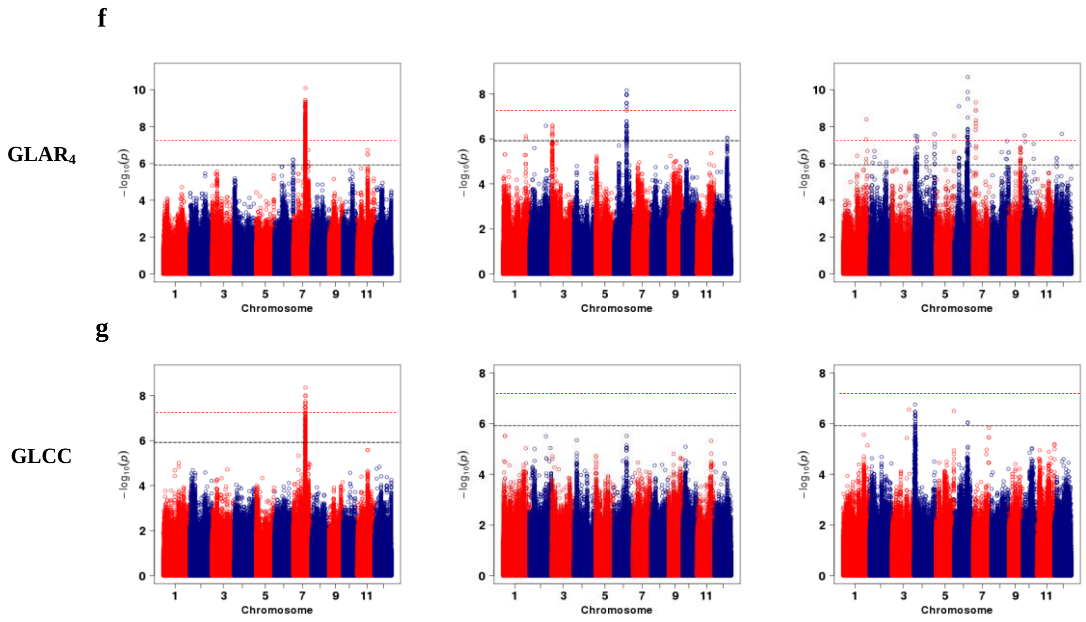

Supplementary Figure S6. Genome-wide association studies of 7 (leaf color related) traits at three different growth stages. Manhattan plots of GLA_2(a), GLA_3(b), GLA_4(c), $GLAR_2$(d), $GLAR_3$(e), $GLAR_4$(f), and GLCC(g) at late tillering stage (left), late booting stage (middle), and milk grain stage (right), respectively. The horizontal gray dashed line and red dashed line indicates the genome-wide suggestive threshold (P = 1.21×10^{-6}) and significant threshold (P = 6.03×10^{-8}), respectively.

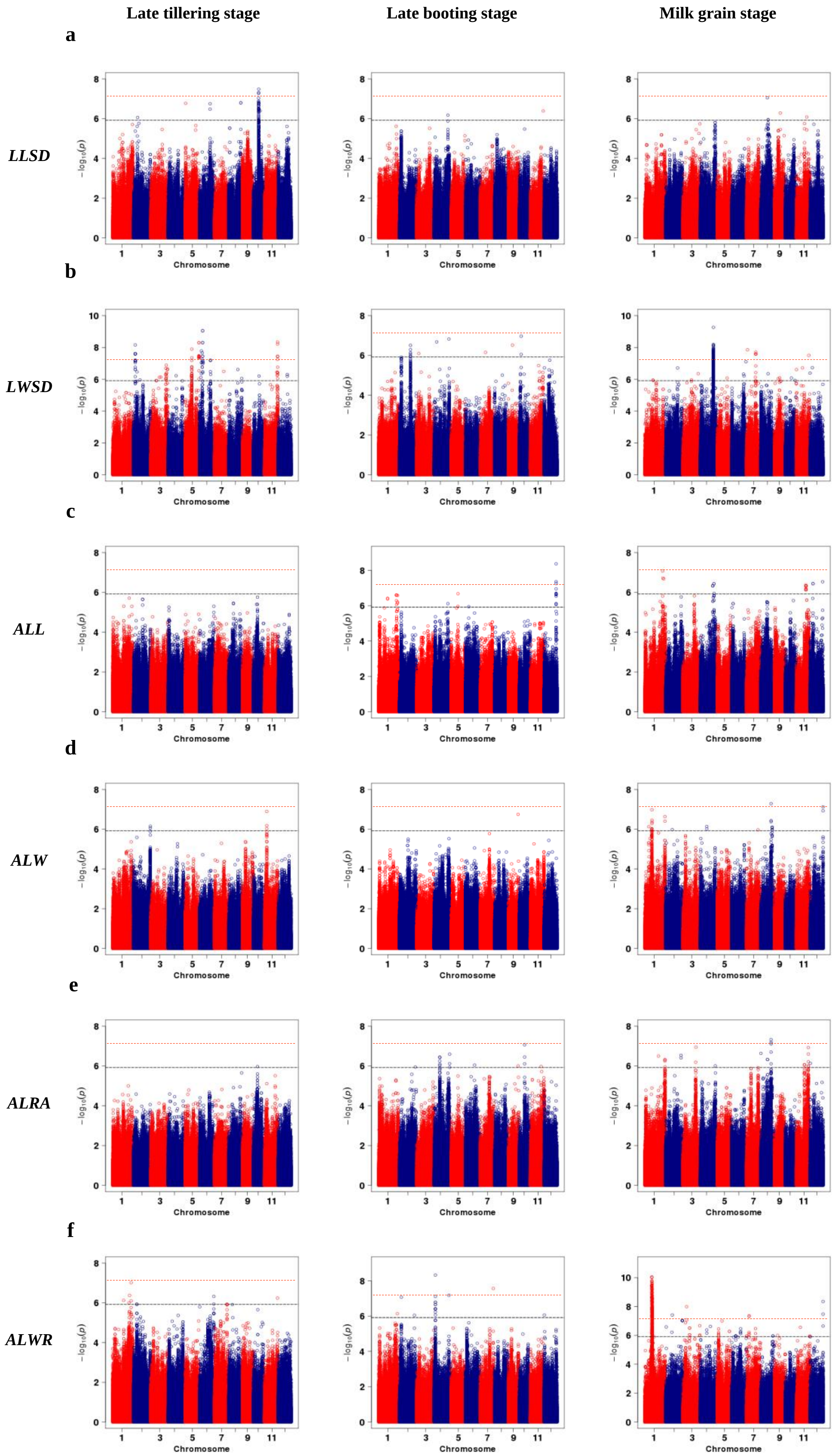
Late tillering stage
Late booting stage
Milk grain stage
a
LLSD
b
LWSD
c
ALL
d
ALW
e
ALRA
f
ALWR
$-\log_{10}(p)$
Chromosome

g

ALP

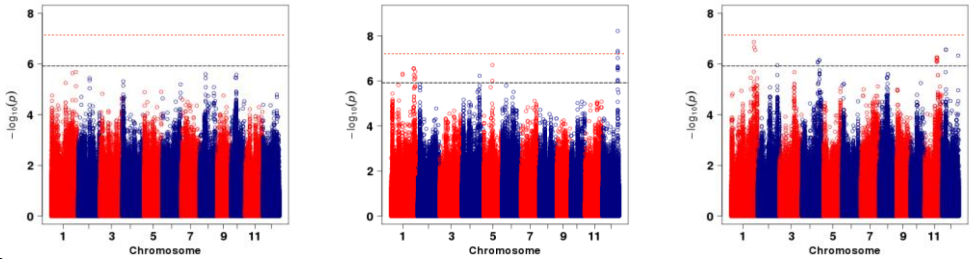

h

APAR

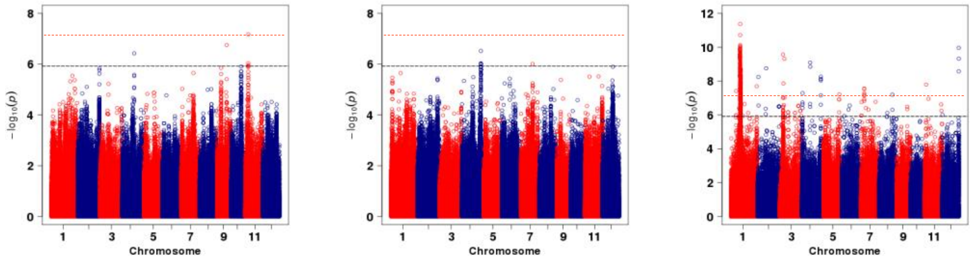

i

ALC

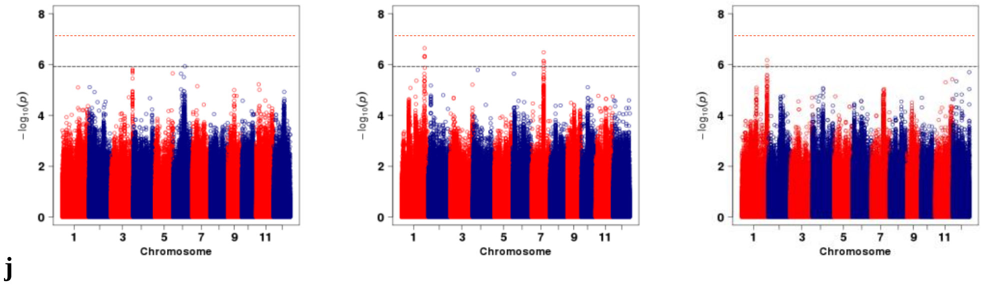

j

MLL

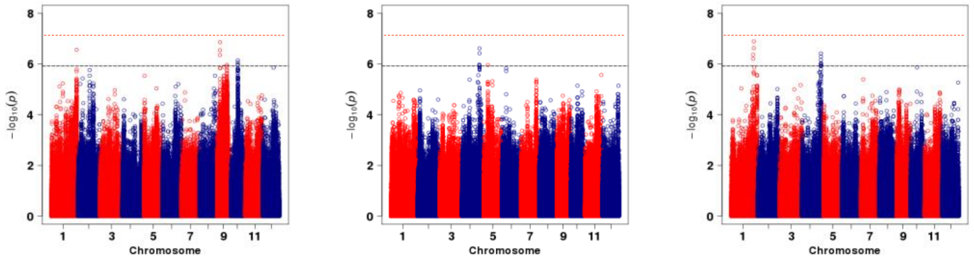

k

MLW

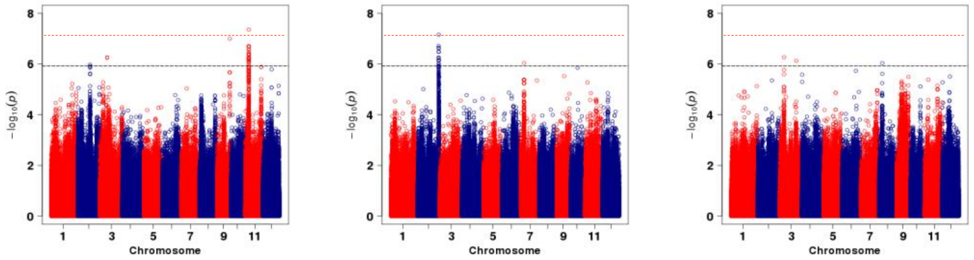

l

MLRA

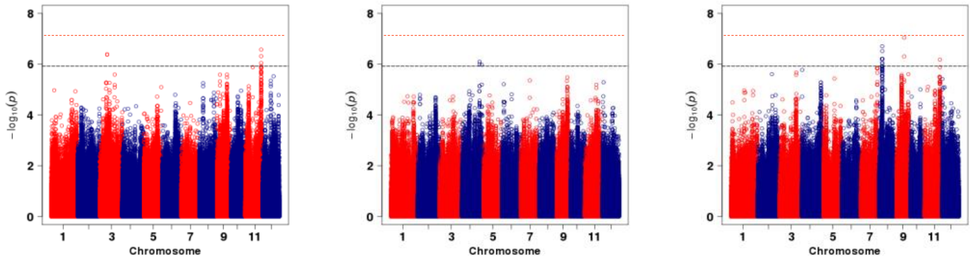

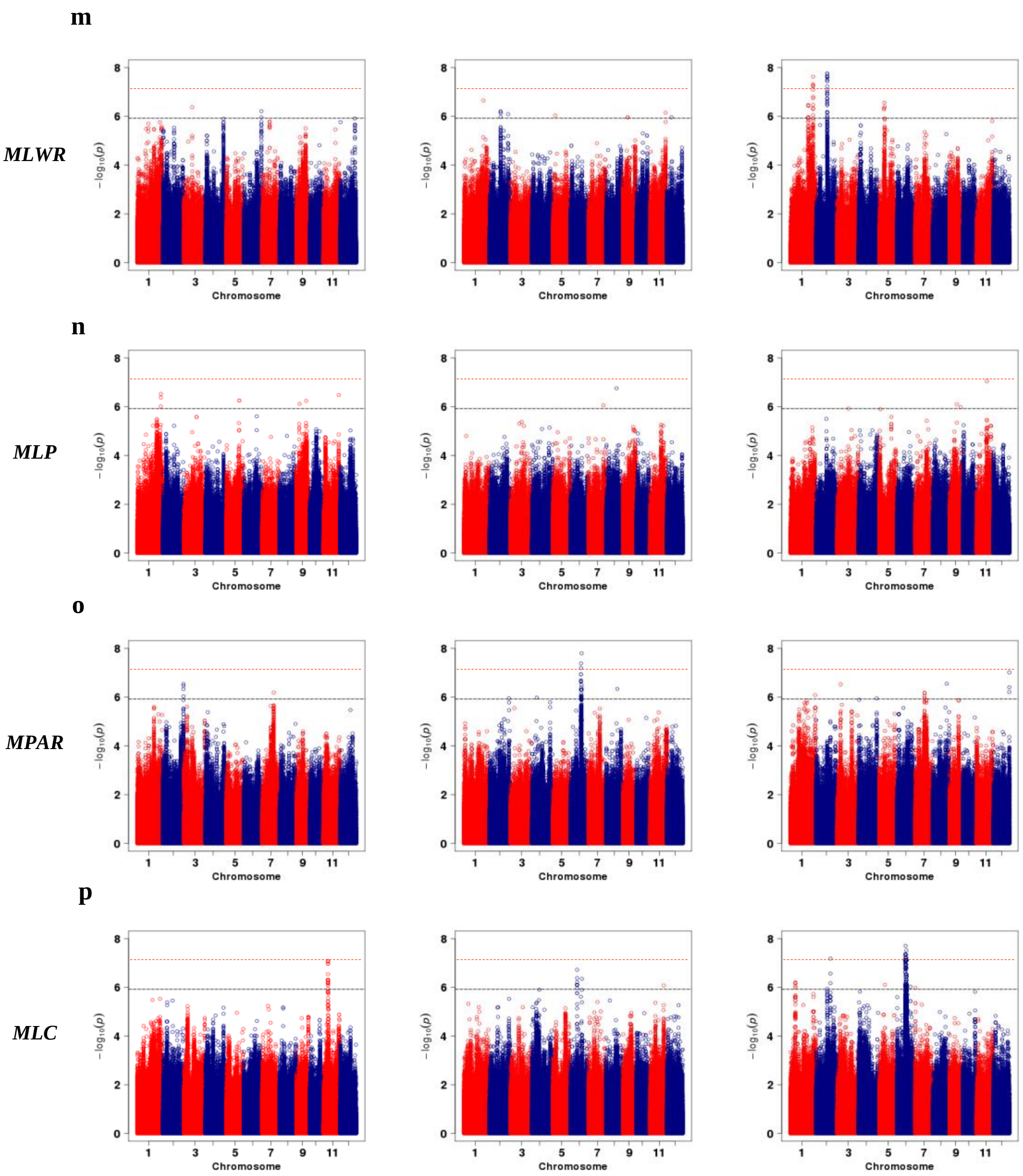

Supplementary Figure S7. Genome-wide association studies of 16 (leaf shape-related) traits at three different growth stages. Manhattan plots of LLSD(a), LWSD(b), ALL(c), ALW(d), ALRA(e), ALWR(f), ALP(g), APAR(h), ALC(i), MLL(j), MLW(k), MLRA(l), MLWR(m), MLP(n), MPAR(o), and MLC(p) at late tillering stage (left), late booting stage (middle), and milk grain stage (right), respectively. The horizontal gray dashed line and red dashed line indicates the genome-wide suggestive threshold (P = 1.21×10^{-6}) and significant threshold (P = 6.03×10^{-8}), respectively.

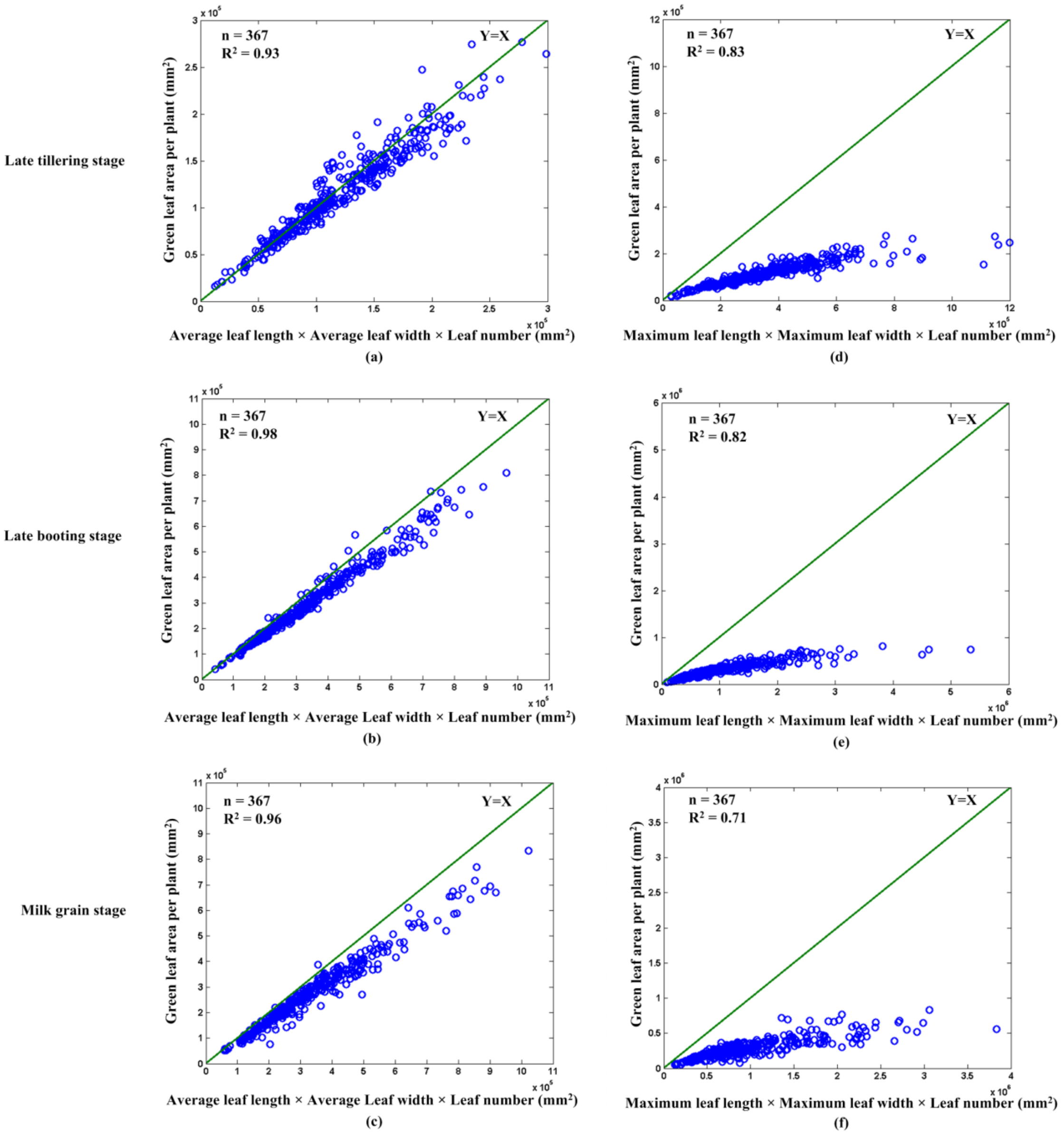

Supplementary Figure S8. Scatter plots of leaf shape measurements versus green leaf area measurements at three growth stages. The relation between the Average leaf length × Average leaf width × leaf number (ALWN) and the green leaf area per plant at (a) late tillering stage, (b) late booting stage, and (c) milk grain stage. The relation between the Maximum leaf length × Maximum leaf width × leaf number (MLWN) and the green leaf area per plant at (d) late tillering stage, (e) late booting stage, and (f) milk grain stage.

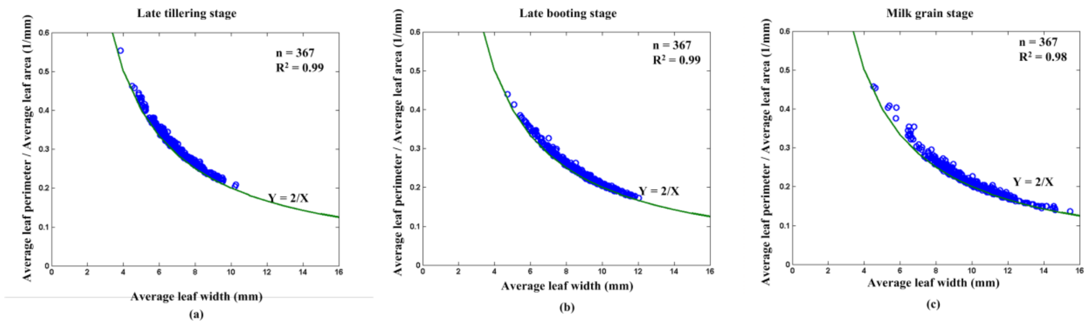

Supplementary Figure S9. Scatter plots of average leaf width measurements versus average leaf perimeter area ratio measurements at three growth stages. (a) late tillering stage, (b) late booting stage, and (c) milk grain stage.

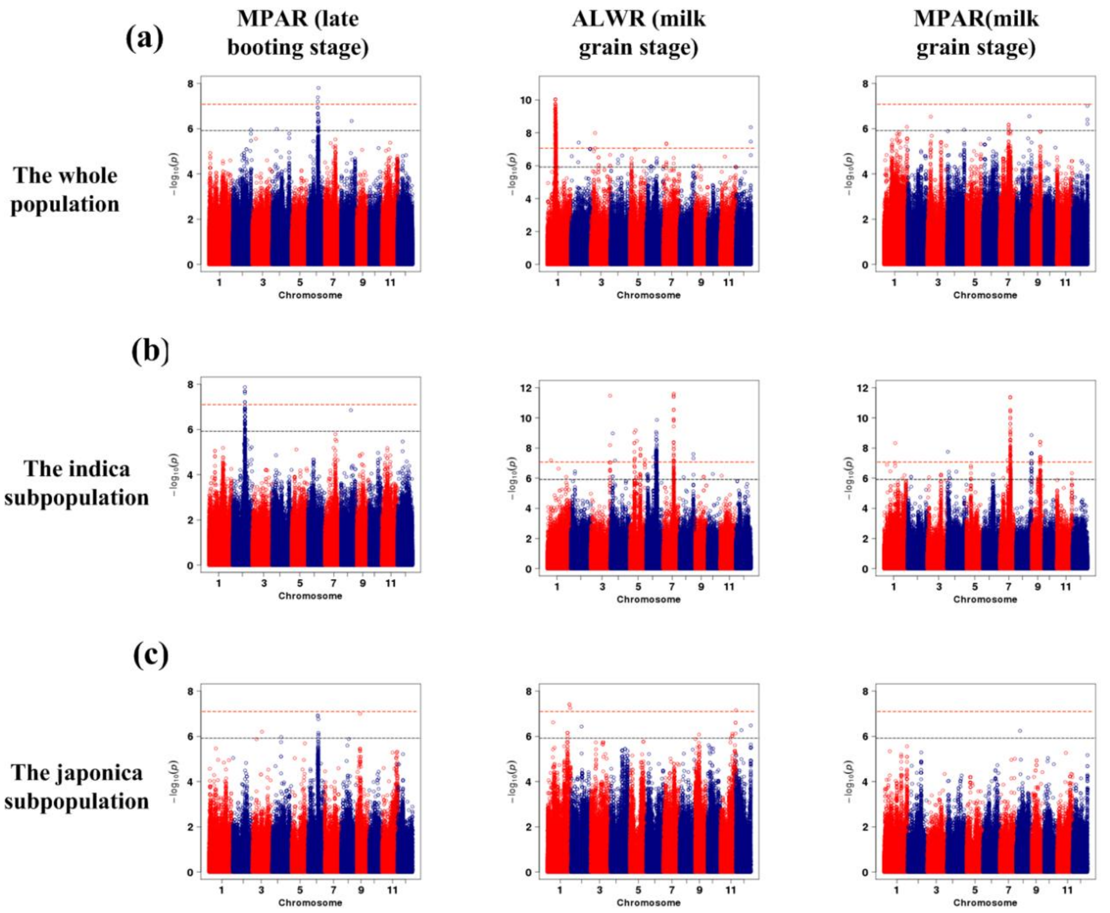

Supplementary Figure S10. GWAS results for three leaf traits in three different (sub)populations.

Manhattan plots of MPAR at late booting stage (left), ALWR at milk grain stage (middle), MPAR at milk grain stage (right) in the whole population (a), the *indica* subpopulation (b), and the *japonica* subpopulation (c). The horizontal gray dashed line and red dashed line indicates the genome-wide suggestive threshold (P = 1.21×10^{-6}) and significant threshold (P = 6.03×10^{-8}), respectively.

Supplementary Notes

1. The source code and annotation of image acquisition

All the software, including image acquisition, image analysis, host-computer control, and human-computer interaction, was designed using LabVIEW (National Instruments Corporation, USA). The system is equipped with a camera-link image acquisition device (IMAQ PCI-1428, National Instruments Corporation, USA) and IMAQ Virtual Instruments (VI) library for LabVIEW. Once the "Single Task Start" button is clicked and the barcode is entered, color images (1280 × 3000) are continuously acquired and analyzed until the "Single Task End" button is clicked. By using a queue to cache the acquired images, the image analysis and image storage procedures parallel the image acquisition procedure, thus optimizing the measurement efficiency.

The procedure for image acquisition contains four main VI elements: IMAQ Grab Setup VI, IMAQ Grab Acquire VI, IMAQ Stop VI, and Enqueue Element VI. IMAQ Grab Setup VI is used to configure the parameters of camera acquisition (such as image size of a single frame, acquisition speed, and so on). IMAQ Grab Acquire VI is used to acquire a single frame of color image. When the single task has ended and the "Single Task End" button is clicked, IMAQ Stop VI is used to end the image acquisition. Here, using a queue to cache the acquired images (using Enqueue Element VI), the procedure of image analysis and image storage parallels the procedure of image acquisition, thus optimizing the measurement efficiency. The source code for image acquisition is shown in Supplementary Note Fig. N1.

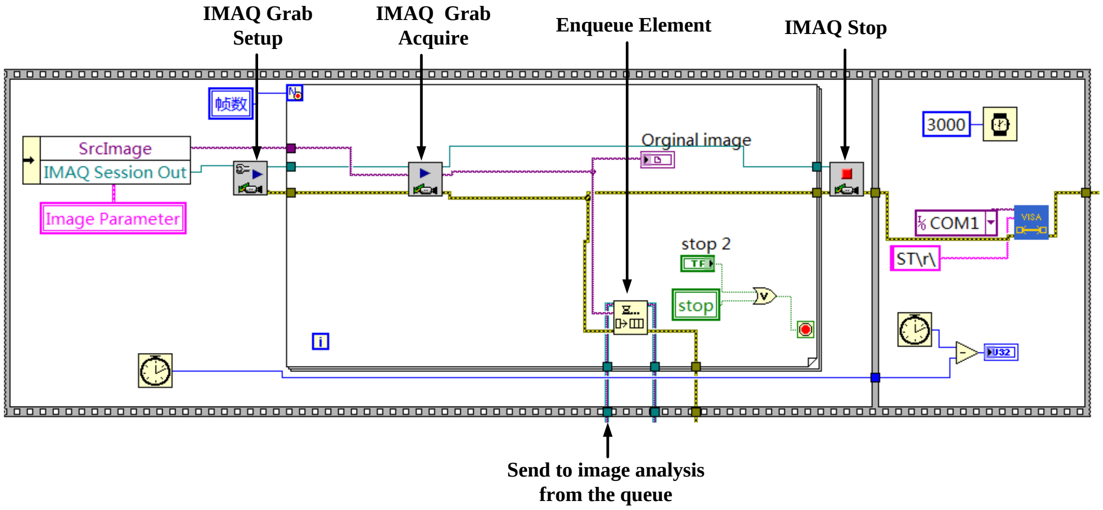

Fig. N1. The source code for image acquisition.

2. The source code and annotation of image correction

Three grayscale images (R, G, and B components) are extracted using IMAQ ExtractColorPlanes VI. With predefined values (7 and 14), the grayscale images of the G and B components are shifted up to be level with the position of the R grayscale image (IMAQ Shift VI). At last, the three grayscale images are merged back to a corrected RGB color image using IMAQ ReplaceColorPlane VI. The source code for image correction is shown in Supplementary Note Fig. N2 and Fig. N3.

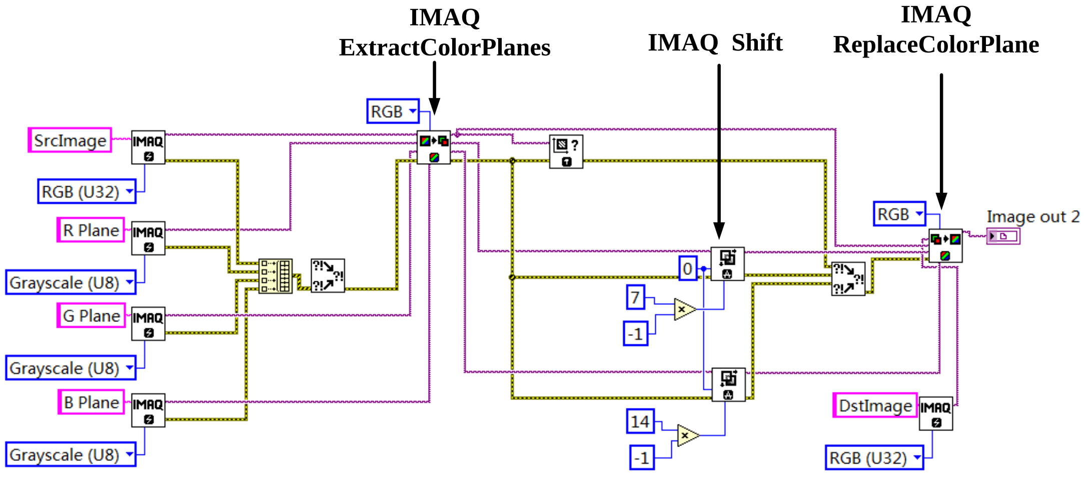

Fig. N2. The source code for image correction.

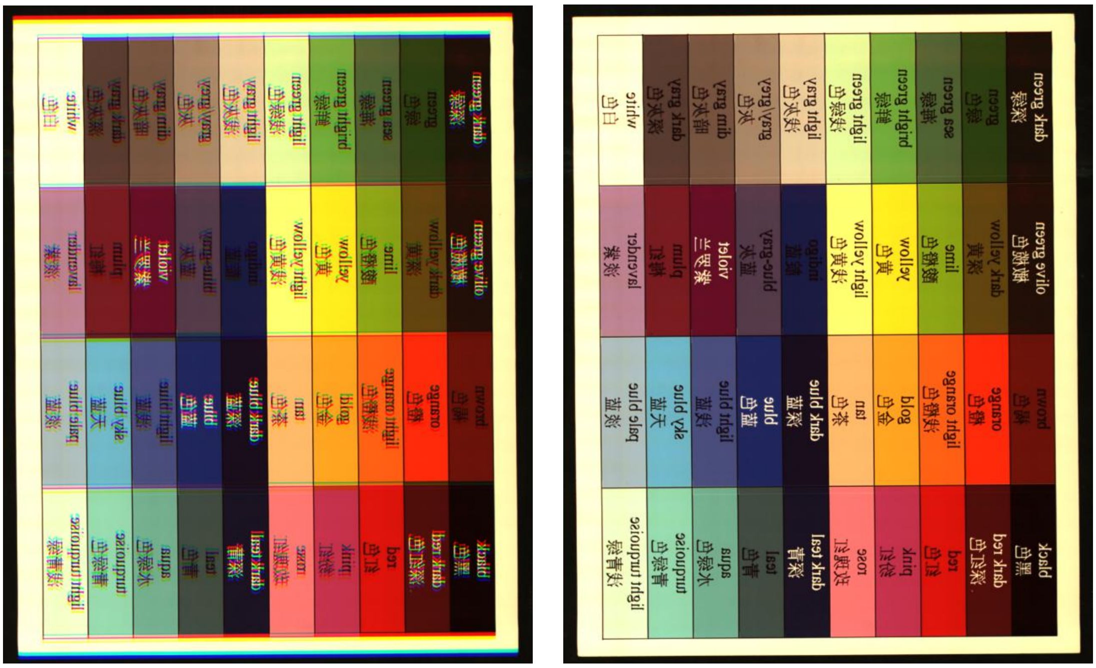

Fig. N3. Original color image acquired by color line-scan camera (left) and the corrected image (right) of a color checker.

3. The source code and annotation of image processing

After the RGB color image is corrected, the image reprocessing procedures include the following steps: (1) to eliminate the noise of the bilateral borders of the conveyor the left border (100 pixels, approximately 20 mm) and right border (100 pixels, approximately 20 mm) of the image are cut using a IMAQ Extract VI; (2) with the predefined segmentation values in HSL color space, the foreground regions of interest with impurities are extracted using IMAQ ColorThreshold VI; (3) a IMAQ RemoveParticle VI is used to remove the impurities; (4) then a IMAQ FillHole VI is used to fill the hole of the rice leaf; (5) after image cropping, the leaves part in the previous image, which are connected to the lower boundary, are cut and reconnected to the remaining leaves part of the current image. The source code of image preprocessing is demonstrated in Supplementary Note Fig. N4.

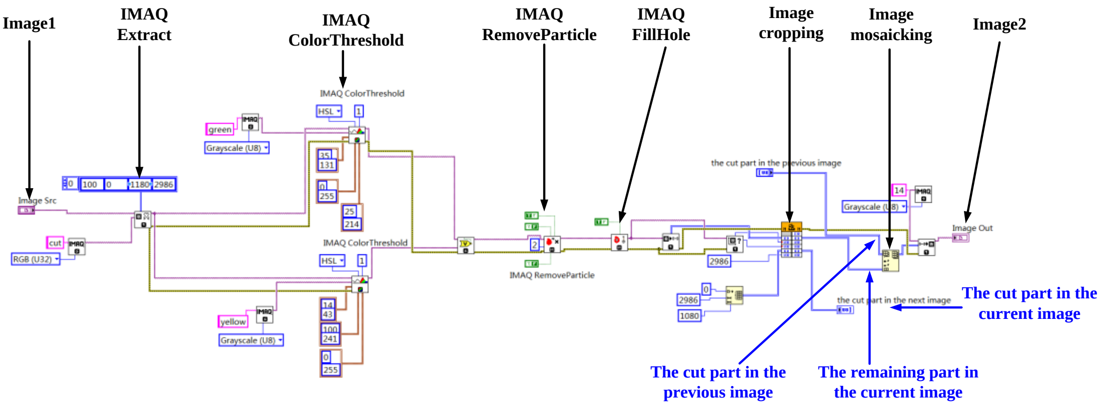

Fig. N4. The source code of image preprocessing with LabVIEW.

As some dust and dead leaf tip may appear on the mosaicking image, after removing the isolated pixels, the binary leaf image without impurities can be extracted. Then the leaf number and total leaf area can be extracted. The source code of removing impurities is demonstrated in Supplementary Note Fig. N5.

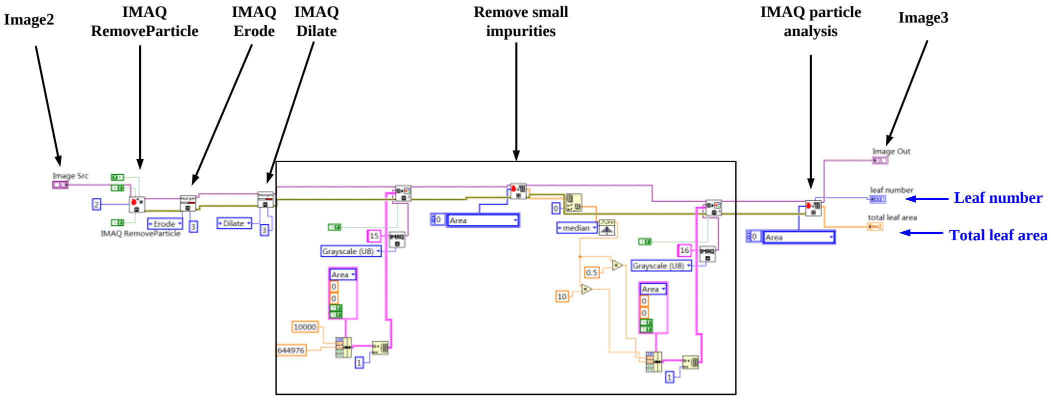

Fig. N5. The source code of removing impurities of mosaicking image with LabVIEW.

After the binary leaf image without impurities can be extracted, to ensure the accuracy of the leaf morphological traits extraction, some severe-curly leaves are rejected. Then the morphological traits of each leaf, including leaf length, leaf width, leaf perimeter, leaf area, and leaf compactness can be extracted. For example, using the modified hilditch thinning algorithm, the main path length can be obtained and count. And the real leaf length is determined by the path length and the spatial resolution of the inspection unit (0.22 *mm/pixel*). The source code of leaf morphological traits extraction is demonstrated in Supplementary Note Fig. N6.

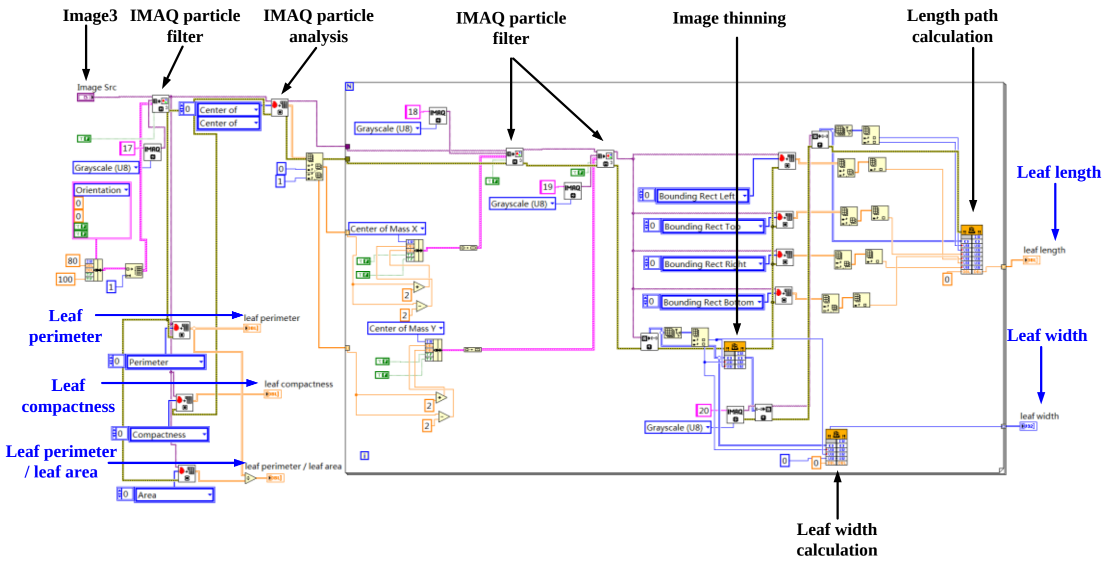

Fig. N6. The source code of leaf morphological traits extraction with LabVIEW.

After the images (Image1) are segmented with predefined values in HLS color space for green leaf area, the total leaf image without impurities (Image3) is also obtained as a mask for the subsequent extraction of GLA, GLA_2, GLA_3, and GLA_4. Here, we use the extraction of GLA and GLA_2 as an example. In a subsequent step, the pure green (or green-2) leaf is determined by the set intersection of the mask and the segmented region with impurities. At last, the foreground pixel numbers of green leaf (or green-2 leaf) are counted, respectively. When the single task has ended (such as the total leaves of an individual plant have all been measured with the HLS), the leaf area is determined by the sum of pixel numbers of the current task and the spatial resolution of the inspection system (0.048 mm^2*/pixel*).

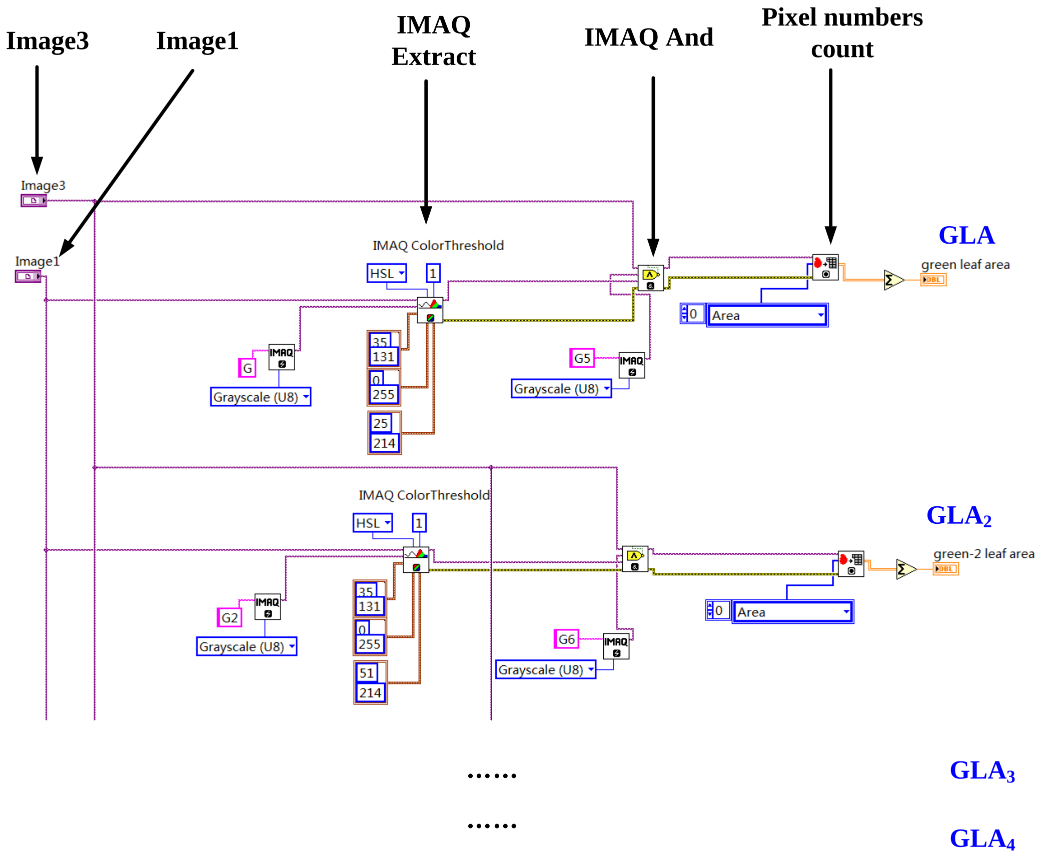

Fig. N7. The source code of leaf area extraction with LabVIEW.

4. The predefined segmentation values in HSL color space are listed in Table N1.

Table. N1. The hardware control protocol.

Traits	H threshold value	S threshold value	L threshold value
GLA	35-131	0-255	25-214
GLA_2	35-131	0-255	51-214
GLA_3	35-131	0-255	38-50
GLA_4	35-131	0-255	25-37
YLA	14-43	100-241	100-255

5. The hardware control protocol is listed in Table N2, and the source code of PLC is shown in Fig. N8 and Fig. N9. A programmable logic controller (PLC, CP1H-Y20DT-D, Omron Corporation, Japan) is adopted in the system to regulate the conveyor and communicate with the workstation. The conveyor is driven by a servo motor (MHMD042P1U, Panasonic Corporation, Japan) and a servo

driver (MBDDT2210003, Panasonic Corporation, Japan). The servo driver is controlled by the pulse output of the PLC, which controls the running speed, the acceleration speed, and the deceleration speed of the conveyor. To achieve host-computer control, the communication protocol between the workstation and the PLC should be predefined. The hardware control protocol and the source code of the PLC (as designed by CX-Programmer, Version 7.30, Omron Corporation, Japan) are described.

Table. N2. The hardware control protocol.

Instruction from workstation	Instruction from PLC	The operation of "Single Task Start" button on the right side of the HLS	The operation of the software interface	Function
"R?"	"OK"	-	-	Set the flag of system running
"ST"	-	-	-	Stop the conveyor
-	-	ON	-	Run the conveyor
-	-	OFF	-	Stop the conveyor
-	-	-	After the barcode is input	Start the continuous image acquisition and image analysis
-	-	-	After "Single Task End" is clicked	Stop the image acquisition and send the instruction "ST" to PLC

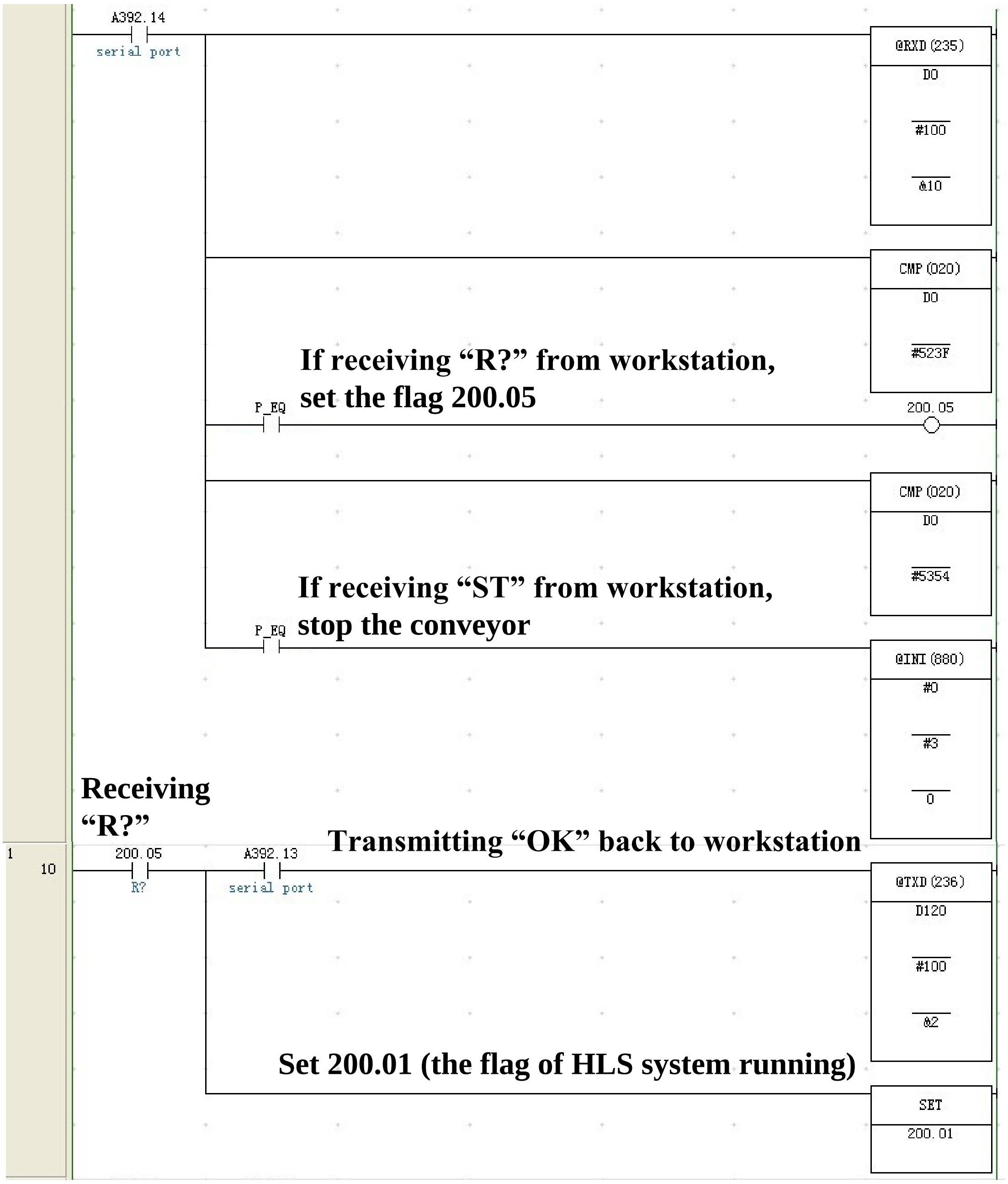

Fig. N8. The source code of the PLC (part 1).

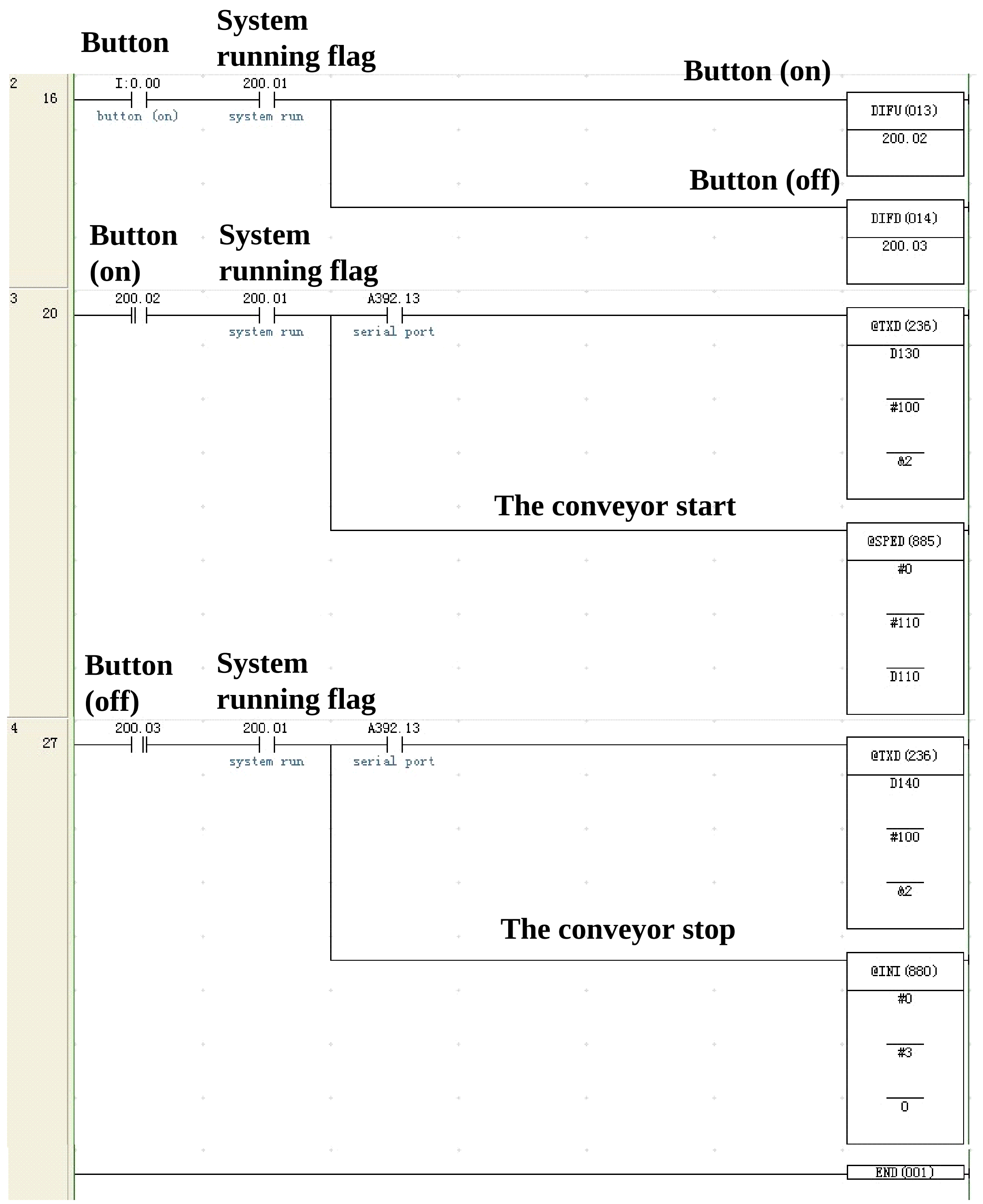

Fig. N9. The source code of the PLC (part 2).

6. Associated lead SNPs of loci linked to known genes

The distance of the lead SNP sf0331940902 of the locus 81 associated with GLA_2 at late tillering stage and a known leaf development-related gene *OSH43* is approximately 17 kb. The lead SNP sf0516433687 of locus 130 associated with GLA_2 at late tillering stage is approximately 21 kb away from a rice leaf color-related gene *YGL1*. The lead SNP sf 0300610972 of locus 58 associated with $GLAR_3$ at late tillering stage is approximately 54 kb away from a rice Class III homeodomain leucine

zipper (Class III HD-Zip) gene *OSHB1* related to establishment of polarity in developing leaves. The lead SNP sf1018007376 of locus 238 associated with GLA_2 at late tillering stage is approximately 7 kb away from another rice Class III homeodomain leucine zipper (Class III HD-Zip) gene, *OSHB2*. The lead SNPs sf0430972316 associated with *LWSD* at late booting stage, sf0431040266 associated with *ALWR* at late booting stage, sf0431078190 associated with *APAR* at late booting stage , sf0431039801 associated with *APAR* and *MPAR* at milk grain stage of locus 109 are approximately 46, 11, 49, 10 kb away from a known gene *Nal1* affecting rice leaf blade width. The lead SNP sf1225260606 and sf1225286279 of locus 285 associated with *ALL* and *ALP* at late booting stage are approximately 31 kb and 6kb away from *OsIAA3* gene controlling rice leaf length, respectively. The lead SNP sf1200141009 of locus 266 associated with *LWSD* at milk grain stage is approximately 77 kb away from *NAL3* gene encoding WUSCHEL-related homeobox 3A (OsWOX3A) affecting rice leaf width. The lead SNP sf1021751079 of locus 240 associated with GLA_2 at late tillering stage is approximately 60kb away from *OsNRT1*, a gene responsible for transporting nitrate.

Supplementary Table S1 Leaf traits calculations with the HLS.

Classifications	Abbreviation	Definition	Calculation
Leaf size related	LN	Leaf number per plant	Total leaf number per plant.
	GLA	Green leaf area per plant (mm^2)	Represent the area of total green leaves per plant.
	TLA	Total leaf area per plant (mm^2)	Represent the area of total leaves (including green leaves and yellow leaves) per plant.
	GLAR	Green leaf area ratio	$GLAR = \frac{GLA}{TLA}$, *GLA* is the green leaf area, and *TLA* is the total leaf area.
	ALA	Average leaf area (mm^2)	$ALA = \frac{1}{LN}\sum_{i=1}^{LN} x_{Ai}$, x_{Ai} is the leaf area of *ith* leaf, and *LN* is the leaf number per plant.
	MLA	Maximum leaf area(mm^2)	$MLA = MAX(x_{Ai}), \quad i = 1, 2, ..., LN$, x_{Ai} is the leaf area of *ith* leaf, and *LN* is the leaf number per plant.
Leaf color related	GLA_2	Green-2 leaf area per plant (mm^2)	Represent the leaves whose color is similar to the group 2 of the leaf color chart designed by the IRRI.
	GLA_3	Green-3 leaf area per plant (mm^2)	Represent the leaves whose color is similar to the group 3 of the leaf color chart designed by the IRRI.
	GLA_4	Green-4 leaf area per plant (mm^2)	Represent the leaves whose color is similar to the group 4 of the leaf color chart designed by the IRRI.
	$GLAR_2$	Green-2 leaf area ratio	$GLAR_2 = \frac{GLA_2}{GLA}$, GLA_2 is the green-2 leaf area per plant, and *GLA* is the green leaf area per plant.
	$GLAR_3$	Green-3 leaf area ratio	$GLAR_3 = \frac{GLA_3}{GLA}$, GLA_3 is the green-3 leaf area per plant, and *GLA* is the green leaf area per plant.

	$GLAR_4$	Green-4 leaf area ratio	$GLAR_4 = \frac{GLA_4}{GLA}$, GLA_4 is the green-4 leaf area per plant, and *GLA* is the green leaf area per plant.
	GLCC	Green leaf color classification	the rice leaves are classified into group *i*, such that $GLAR_i = \max(GLAR_2, GLAR_3, GLAR_4), \quad i = 2,3,4.$ $GLAR_2$ is the green-2 leaf area ratio, $GLAR_3$ is the green-3 leaf area ratio, and $GLAR_4$ is the green-4 leaf area ratio.
Leaf shape related	LLSD	Leaf length standard deviation per plant (mm)	$LLSD = \sqrt{\frac{1}{LN}\sum_{i=1}^{LN}(x_{li} - \mu_l)^2}$, x_{li} is the leaf length of *ith* leaf and μ_l is the average value of the leaf length per plant.
	LWSD	Leaf width standard deviation per plant (mm)	$LWSD = \sqrt{\frac{1}{LN}\sum_{i=1}^{LN}(x_{wi} - \mu_w)^2}$, x_{wi} is the leaf width of *ith* leaf and μ_w is the average value of the leaf width per plant.
	ALL	Average leaf length (mm)	$ALL = \frac{1}{LN}\sum_{i=1}^{LN} x_{li}$, x_{li} is the leaf length of *ith* leaf.
	ALW	Average leaf width (mm)	$ALW = \frac{1}{LN}\sum_{i=1}^{LN} x_{wi}$, x_{wi} is the leaf width of *ith* leaf.
	ALRA	Average leaf rectangle area (mm^2)	$ALRA = \frac{1}{LN}\sum_{i=1}^{LN} x_{li} \times x_{wi}$, x_{li} is the leaf length of *ith* leaf, x_{wi} is the leaf width of *ith* leaf.
	ALWR	Average leaf length/leaf width ratio	$ALWR = \frac{1}{LN}\sum_{i=1}^{LN} \frac{x_{li}}{x_{wi}}$, x_{li} is the leaf length of *ith* leaf, x_{wi} is the leaf width of *ith* leaf.
	ALP	Average leaf perimeter (mm)	$ALP = \frac{1}{LN}\sum_{i=1}^{LN} x_{pi}$, x_{pi} is the leaf perimeter of *ith* leaf.
	APAR	Average leaf perimeter/leaf area ratio (1/mm)	$APAR = \frac{1}{LN}\sum_{i=1}^{LN} \frac{x_{pi}}{x_{Ai}}$, x_{pi} is the leaf perimeter of *ith* leaf, x_{Ai} is the leaf area of *ith* leaf.
	ALC	Average leaf compactness	$ALC = \frac{1}{LN}\sum_{i=1}^{LN} \frac{x_{Ai}}{x_{li} \times x_{wi}}$, x_{Ai} is the leaf area of *ith* leaf, x_{li} is the leaf length of *ith* leaf, x_{wi} is the leaf width of *ith* leaf.
	MLL	Maximum leaf length (mm)	$MLL = MAX(x_{li}), \quad i = 1,2,...,LN$, x_{li} is the leaf length of *ith* leaf.

MLW	Maximum leaf width (mm)	$MLW = MAX(x_{wi}), \quad i = 1,2,...,LN$, x_{wi} is the leaf width of *ith* leaf.
MLRA	Maximum leaf rectangle area(mm^2)	$MLRA = MAX(x_{li} \times x_{wi}), \quad i = 1,2,...,LN$, x_{li} is the leaf length of *ith* leaf, x_{wi} is the leaf width of *ith* leaf.
MLWR	Maximum leaf length/leaf width ratio	$MLWR = MAX(\frac{x_{li}}{x_{wi}}), \quad i = 1,2,...,LN$, x_{li} is the leaf length of *ith* leaf, x_{wi} is the leaf width of *ith* leaf.
MLP	Maximum leaf perimeter (mm)	$MLP = MAX(x_{pi}), \quad i = 1,2,...,LN$, x_{pi} is the leaf perimeter of *ith* leaf.
MPAR	Maximum leaf perimeter/leaf area ratio (1/mm)	$MPAR = MAX(\frac{x_{pi}}{x_{Ai}}), \quad i = 1,2,...,LN$, x_{pi} is the leaf perimeter of *ith* leaf, x_{Ai} is the leaf area of *ith* leaf.
MLC	Maximum leaf compactness	$MLC = MAX(\frac{x_{Ai}}{x_{li} \times x_{wi}}), \quad i = 1,2,...,LN$, x_{Ai} is the leaf area of *ith* leaf, x_{li} is the leaf length of *ith* leaf, x_{wi} is the leaf width of *ith* leaf.

Supplementary Table S2 Effective number of SNPs across rice genome and LD-adjusted Bonferroni corrected p-value thresholds

Chromosome	Observed_Number	Effective_Number	Effective_Ratio	Suggestive_P_Value	Significant_P_Value
1	498272	87561	0.18	1.14E-05	5.71E-07
2	387222	67305	0.17	1.49E-05	7.43E-07
3	381693	60705	0.16	1.65E-05	8.24E-07
4	372273	76170	0.20	1.31E-05	6.56E-07
5	309780	50767	0.16	1.97E-05	9.85E-07
6	364052	65485	0.18	1.53E-05	7.64E-07
7	343840	64962	0.19	1.54E-05	7.70E-07
8	345123	70740	0.20	1.41E-05	7.07E-07
9	278739	55701	0.20	1.80E-05	8.98E-07
10	316925	61777	0.19	1.62E-05	8.09E-07
11	398281	89597	0.22	1.12E-05	5.58E-07
12	362400	78681	0.22	1.27E-05	6.35E-07
Genome	4358600	829451	0.19	1.21E-06	6.03E-08

Supplementary table S3 GWAS results with the suggestive *P* value threshold.

Stage	Trait	Lead SNP ID	Chr.	Position	Locus ID	*P* value	Known genes
	ALC	sf0619345108	6	19345108	**156**	1.16E-06	
	ALRA	sf1008833338	10	8833338	**232**	1.07E-06	
	ALW	sf0235130542	2	35130542	**56**	7.09E-07	
	ALW	sf1104981947	11	4981947	**245**	1.26E-07	
	ALWR	sf0122803148	1	22803148	**11**	7.48E-07	
	ALWR	sf0138403712	1	38403712	**23**	9.57E-08	
	ALWR	sf0207080365	2	7080365	**34**	1.18E-06	
	ALWR	sf0630075765	6	30075765	**163**	4.73E-07	
	ALWR	sf0726335488	7	26335488	**186**	1.17E-06	
	ALWR	sf1127368298	11	27368298	**264**	5.75E-07	
	APAR	sf0419609623	4	19609623	**103**	3.77E-07	
	APAR	sf0915958783	9	15958783	**219**	1.76E-07	
	APAR	sf1104981947	11	4981947	**245**	6.73E-08	
	GLA	sf0102578034	1	2578034	**1**	6.08E-08	
	GLA	sf0207450950	2	7450950	**35**	4.61E-07	
	GLA_2	sf0108312563	1	8312563	**2**	5.45E-07	
	GLA_2	sf0114076144	1	14076144	**4**	1.13E-07	
	GLA_2	sf0114653019	1	14653019	**5**	9.61E-09	
	GLA_2	sf0115987001	1	15987001	**6**	1.26E-07	
	GLA_2	sf0127794903	1	27794903	**12**	8.19E-07	
	GLA_2	sf0137212688	1	37212688	**20**	1.06E-08	
	GLA_2	sf0138573557	1	38573557	**23**	2.90E-07	
	GLA_2	sf0138779837	1	38779837	**24**	8.91E-08	
	GLA_2	sf0204315109	2	4315109	**32**	1.81E-07	
	GLA_2	sf0207082062	2	7082062	**34**	5.11E-07	
	GLA_2	sf0207484769	2	7484769	**35**	8.12E-09	
	GLA_2	sf0211452728	2	11452728	**39**	7.34E-07	
	GLA_2	sf0305695371	3	5695371	**62**	4.50E-08	
	GLA_2	sf0306531323	3	6531323	**64**	9.56E-07	
	GLA_2	sf0313954229	3	13954229	**69**	5.55E-08	
	GLA_2	sf0324314764	3	24314764	**75**	7.69E-07	
	GLA_2	sf0331940902	3	31940902	**81**	7.56E-09	***OSH43***
	GLA_2	sf0406027943	4	6027943	**93**	2.94E-10	
	GLA_2	sf0409991261	4	9991261	**96**	2.38E-07	
	GLA_2	sf0412471343	4	12471343	**98**	3.35E-07	
	GLA_2	sf0434222733	4	34222733	**115**	7.05E-07	
	GLA_2	sf0502421097	5	2421097	**117**	2.89E-09	
	GLA_2	sf0513728565	5	13728565	**126**	5.48E-12	
	GLA_2	sf0514135932	5	14135932	**127**	7.82E-08	
	GLA_2	sf0514281006	5	14281006	**127**	1.93E-07	
	GLA_2	sf0514489026	5	14489026	**128**	3.18E-07	
	GLA_2	sf0514804131	5	14804131	**129**	6.07E-07	
	GLA_2	sf0516433687	5	16433687	**130**	1.76E-09	***YGL1***
	GLA_2	sf0518905497	5	18905497	**131**	9.70E-07	
	GLA_2	sf0525748215	5	25748215	**136**	8.10E-09	
	GLA_2	sf0618243370	6	18243370	**155**	1.11E-07	
	GLA_2	sf0630583038	6	30583038	**164**	4.66E-08	
	GLA_2	sf0700219702	7	219702	**166**	4.36E-09	
	GLA_2	sf0711200104	7	11200104	**174**	1.43E-09	
	GLA_2	sf0804368745	8	4368745	**190**	3.05E-09	
	GLA_2	sf0822950418	8	22950418	**202**	6.53E-08	
	GLA_2	sf0826476003	8	26476003	**206**	1.47E-10	
	GLA_2	sf0905443985	9	5443985	**210**	1.44E-08	
	GLA_2	sf0908834656	9	8834656	**214**	8.61E-07	
	GLA_2	sf0914005048	9	14005048	**217**	7.55E-07	
	GLA_2	sf1000985386	10	985386	**227**	1.40E-07	
	GLA_2	sf1002442905	10	2442905	**229**	8.39E-07	

	GLA_2	sf1017768320	10	17768320	**237**	1.94E-07	
	GLA_2	sf1018007376	10	18007376	**238**	8.90E-07	***OSHB2***
	GLA_2	sf1021751079	10	21751079	**240**	1.40E-07	***OsNRT1***
	GLA_2	sf1022775806	10	22775806	**241**	2.23E-09	
	GLA_2	sf1104320861	11	4320861	**244**	5.03E-07	
	GLA_2	sf1113756716	11	13756716	**253**	4.72E-07	
	GLA_2	sf1126753044	11	26753044	**262**	1.73E-08	
	GLA_2	sf1202270243	12	2270243	**269**	7.19E-09	
	GLA_2	sf1215302938	12	15302938	**278**	1.57E-07	
	GLA_2	sf1215471534	12	15471534	**278**	1.17E-06	
	GLA_2	sf1215668235	12	15668235	**279**	1.21E-08	
	GLA_2	sf1224620960	12	24620960	**284**	4.50E-09	
	GLA_2	sf1022040541	10	22040541	**289**	7.88E-07	***Oshox1***
	GLA_3	sf0207447476	2	7447476	**35**	9.31E-07	
	GLA_3	sf0300228477	3	228477	**57**	5.60E-07	
	GLA_3	sf0514281006	5	14281006	**127**	5.20E-08	
	GLA_3	sf0603027903	6	3027903	**142**	5.93E-07	
	GLA_3	sf0626148781	6	26148781	**162**	6.25E-07	
	GLAR	sf0129629431	1	29629431	**14**	2.14E-10	
	GLAR	sf0130806251	1	30806251	**16**	7.01E-08	
	GLAR	sf0133369195	1	33369195	**19**	3.60E-07	
	GLAR	sf0139225674	1	39225674	**25**	6.17E-07	
	GLAR	sf0233343719	2	33343719	**54**	4.85E-07	
	GLAR	sf0317027007	3	17027007	**70**	3.11E-07	
	GLAR	sf0321299161	3	21299161	**73**	3.48E-07	
**Late tilling stage**	*GLAR*	sf0323995809	3	23995809	**74**	1.31E-09	
	GLAR	sf0432141352	4	32141352	**112**	8.42E-08	
	GLAR	sf0616715458	6	16715458	**154**	4.60E-08	
	GLAR	sf0718737228	7	18737228	**178**	1.91E-07	
	GLAR	sf0915023106	9	15023106	**218**	9.17E-07	
	GLAR	sf1106112328	11	6112328	**246**	2.48E-08	
	GLAR	sf1209698383	12	9698383	**275**	2.15E-07	
	$GLAR_2$	sf0131154419	1	31154419	**17**	1.05E-07	
	$GLAR_2$	sf1210907185	12	10907185	**276**	1.01E-06	
	$GLAR_3$	sf0210670093	2	10670093	**38**	1.34E-07	
	$GLAR_3$	sf0219792797	2	19792797	**45**	7.23E-07	
	$GLAR_3$	sf0300610972	3	610972	**58**	1.18E-06	***OSHB1***
	$GLAR_3$	sf0308357750	3	8357750	**67**	3.70E-07	
	$GLAR_3$	sf0325211812	3	25211812	**76**	5.97E-07	
	$GLAR_3$	sf0400994591	4	994591	**85**	1.02E-07	
	$GLAR_3$	sf0401713624	4	1713624	**86**	1.89E-07	
	$GLAR_3$	sf0402215064	4	2215064	**87**	6.30E-07	
	$GLAR_3$	sf0529205395	5	29205395	**140**	2.65E-08	
	$GLAR_3$	sf0612438676	6	12438676	**150**	2.38E-07	
	$GLAR_3$	sf0630973534	6	30973534	**165**	5.76E-07	
	$GLAR_3$	sf0708241028	7	8241028	**173**	1.62E-08	
	$GLAR_3$	sf0715989750	7	15989750	**176**	1.10E-06	
	$GLAR_3$	sf0719639267	7	19639267	**180**	1.94E-13	
	$GLAR_3$	sf0719728350	7	19728350	**180**	3.62E-08	
	$GLAR_3$	sf0719817763	7	19817763	**180**	6.19E-11	
	$GLAR_3$	sf0719861668	7	19861668	**181**	7.43E-08	
	$GLAR_3$	sf0719933345	7	19933345	**181**	5.54E-08	
	$GLAR_3$	sf0810028277	8	10028277	**193**	2.26E-07	
	$GLAR_3$	sf1011748677	10	11748677	**234**	1.41E-07	
	$GLAR_3$	sf1015597665	10	15597665	**236**	7.94E-07	
	$GLAR_3$	sf1117150282	11	17150282	**254**	2.07E-10	
	$GLAR_4$	sf0630973534	6	30973534	**165**	6.39E-07	
	$GLAR_4$	sf0719732764	7	19732764	**180**	1.02E-08	
	$GLAR_4$	sf0719859898	7	19859898	**181**	8.10E-11	
	$GLAR_4$	sf0724505838	7	24505838	**184**	1.86E-07	
	$GLAR_4$	sf1117150282	11	17150282	**254**	1.85E-07	

GLCC	sf0719639464	7	19639464	**180**	4.43E-09
LLSD	sf0208795000	2	8795000	**37**	8.71E-07
LLSD	sf0501147707	5	1147707	**116**	1.69E-07
LLSD	sf0622226761	6	22226761	**159**	1.76E-07
LLSD	sf0825379754	8	25379754	**204**	1.59E-07
LLSD	sf1011328985	10	11328985	**233**	3.25E-08
LN	sf0700720606	7	720606	**167**	8.01E-07
LWSD	sf0203791234	2	3791234	**31**	6.91E-09
LWSD	sf0207348150	2	7348150	**35**	3.40E-07
LWSD	sf0213745376	2	13745376	**41**	1.10E-06
LWSD	sf0311817405	3	11817405	**68**	1.21E-06
LWSD	sf0318258416	3	18258416	**71**	7.50E-07
LWSD	sf0321302599	3	21302599	**73**	7.33E-07
LWSD	sf0332469691	3	32469691	**82**	1.30E-07
LWSD	sf0335091878	3	35091878	**83**	2.37E-07
LWSD	sf0416720716	4	16720716	**101**	5.98E-07
LWSD	sf0514011442	5	14011442	**127**	1.27E-08
LWSD	sf0528903428	5	28903428	**139**	4.99E-09
LWSD	sf0603166566	6	3166566	**142**	7.59E-07
LWSD	sf0606820810	6	6820810	**144**	8.94E-10
LWSD	sf0623068763	6	23068763	**160**	6.48E-08
LWSD	sf0719536933	7	19536933	**180**	3.20E-07
LWSD	sf0819105776	8	19105776	**199**	2.44E-07
LWSD	sf0827450584	8	27450584	**207**	8.84E-07
LWSD	sf1011992245	10	11992245	**234**	6.23E-07
LWSD	sf1126452660	11	26452660	**262**	5.56E-07
LWSD	sf1127580568	11	27580568	**264**	4.61E-09
LWSD	sf1219252080	12	19252080	**282**	4.96E-07
MLC	sf1107968499	11	7968499	**250**	8.31E-08
MLL	sf0141126624	1	41126624	**28**	2.75E-07
MLL	sf0905048259	9	5048259	**209**	1.39E-07
MLL	sf0916436027	9	16436027	**220**	1.08E-06
MLL	sf1011136498	10	11136498	**233**	7.17E-07
MLP	sf0140053170	1	40053170	**27**	2.96E-07
MLP	sf0522529850	5	22529850	**134**	5.55E-07
MLP	sf0905622775	9	5622775	**210**	7.59E-07
MLP	sf0916881637	9	16881637	**221**	5.74E-07
MLP	sf1126256678	11	26256678	**261**	3.30E-07
MLRA	sf0311764061	3	11764061	**68**	4.16E-07
MLRA	sf1126135725	11	26135725	**261**	2.67E-07
MLW	sf0219923827	2	19923827	**45**	1.06E-06
MLW	sf0311764061	3	11764061	**68**	5.59E-07
MLW	sf0920969473	9	20969473	**226**	1.01E-07
MLW	sf1105896366	11	5896366	**246**	4.41E-08
MLWR	sf0630075765	6	30075765	**163**	6.15E-07
MPAR	sf0235130542	2	35130542	**56**	2.90E-07
MPAR	sf0719817702	7	19817702	**180**	6.46E-07
TLA	sf0102578034	1	2578034	**1**	1.02E-07
TLA	sf0207450950	2	7450950	**35**	4.56E-07
ALA	sf0411825119	4	11825119	**97**	7.67E-07
ALA	sf0432485240	4	32485240	**113**	2.62E-07
ALA	sf0920565771	9	20565771	**225**	8.09E-07
ALA	sf1011291409	10	11291409	**233**	7.93E-08
ALA	sf1122466326	11	22466326	**258**	6.51E-07
ALC	sf0137140881	1	37140881	**20**	2.25E-07
ALC	sf0719519032	7	19519032	**180**	3.27E-07
ALC	sf0719820524	7	19820524	**181**	8.68E-07
ALL	sf0118752867	1	18752867	**8**	3.83E-07
ALL	sf0118876546	1	18876546	**8**	4.18E-07
ALL	sf0137119263	1	37119263	**20**	2.53E-07
ALL	sf0428806135	4	28806135	**108**	7.71E-07
ALL	sf0514620874	5	14620874	**128**	2.11E-07

	ALL	sf0607194039	6	7194039	**145**	1.15E-06	
	ALL	sf1225260606	12	25260606	**285**	4.31E-09	***OsIAA3***
	ALL	sf1225286279	12	25286279	**285**	5.54E-08	***OsIAA3***
	ALP	sf0118752867	1	18752867	**8**	5.38E-07	
	ALP	sf0118876546	1	18876546	**8**	4.80E-07	
	ALP	sf0137119263	1	37119263	**20**	2.82E-07	
	ALP	sf0428806135	4	28806135	**108**	5.91E-07	
	ALP	sf0514620874	5	14620874	**128**	1.97E-07	
	ALP	sf1225260606	12	25260606	**285**	6.09E-09	***OsIAA3***
	ALP	sf1225286279	12	25286279	**285**	4.68E-08	***OsIAA3***
	ALRA	sf0233324844	2	33324844	**54**	1.13E-06	
	ALRA	sf0411825119	4	11825119	**97**	3.59E-07	
	ALRA	sf0412108993	4	12108993	**97**	8.24E-07	
	ALRA	sf0432485240	4	32485240	**113**	2.54E-07	
	ALRA	sf0619348857	6	19348857	**156**	9.03E-07	
	ALRA	sf0920565771	9	20565771	**225**	9.98E-07	
	ALRA	sf1011291409	10	11291409	**233**	8.65E-08	
	ALRA	sf1122466326	11	22466326	**258**	1.06E-06	
	ALW	sf0920565771	9	20565771	**225**	1.77E-07	
	ALWR	sf0139077542	1	39077542	**25**	7.29E-07	
	ALWR	sf0204078069	2	4078069	**31**	8.53E-08	
	ALWR	sf0231513174	2	31513174	**51**	8.99E-07	
	ALWR	sf0402755134	4	2755134	**89**	7.67E-08	
	ALWR	sf0402803386	4	2803386	**89**	4.71E-09	
	ALWR	sf0431040266	4	31040266	**109**	6.59E-08	***Nal1***
	ALWR	sf0727101845	7	27101845	**187**	2.72E-08	
	ALWR	sf1200688120	12	688120	**267**	8.69E-07	
	APAR	sf0431078190	4	31078190	**109**	3.03E-07	***Nal1***
	APAR	sf0719034927	7	19034927	**179**	9.91E-07	
	GLA	sf0431688161	4	31688161	**111**	1.52E-08	
	GLA	sf1122295601	11	22295601	**258**	9.59E-07	
	GLA_2	sf0620927842	6	20927842	**158**	1.52E-08	
	GLA_2	sf1219472764	12	19472764	**282**	1.10E-06	
	GLA_3	sf0420901284	4	20901284	**105**	2.25E-07	
	GLA_3	sf0431689032	4	31689032	**111**	1.56E-07	
	GLA_3	sf0714716256	7	14716256	**175**	9.00E-07	
	GLA_3	sf0808543315	8	8543315	**192**	4.78E-07	
	GLA_3	sf0908162862	9	8162862	**212**	4.23E-07	
**Late booting stage**	GLA_3	sf1119689449	11	19689449	**256**	1.19E-06	
	GLA_4	sf0431688161	4	31688161	**111**	8.87E-08	
	GLA_4	sf0908170004	9	8170004	**212**	1.83E-07	
	GLA_4	sf1012749918	10	12749918	**235**	2.82E-07	
	GLAR	sf0216062250	2	16062250	**43**	3.46E-08	
	GLAR	sf0618257099	6	18257099	**155**	7.44E-07	
	$GLAR_2$	sf0420901284	4	20901284	**105**	9.57E-07	
	$GLAR_2$	sf0519997210	5	19997210	**132**	5.96E-07	
	$GLAR_4$	sf0137324152	1	37324152	**20**	7.47E-07	
	$GLAR_4$	sf0226671495	2	26671495	**49**	2.64E-07	
	$GLAR_4$	sf0301308003	3	1308003	**59**	2.48E-07	
	$GLAR_4$	sf0620856382	6	20856382	**158**	6.95E-09	
	$GLAR_4$	sf0620860929	6	20860929	**158**	1.07E-06	
	$GLAR_4$	sf1221018570	12	21018570	**283**	8.83E-07	
	LLSD	sf0428785545	4	28785545	**108**	6.62E-07	
	LLSD	sf1126973639	11	26973639	**263**	4.05E-07	
	LN	sf0431688161	4	31688161	**111**	6.07E-09	
	LWSD	sf0223187963	2	23187963	**46**	3.13E-07	
	LWSD	sf0304524956	3	4524956	**61**	8.00E-07	
	LWSD	sf0405537421	4	5537421	**92**	2.09E-07	
	LWSD	sf0430972316	4	30972316	**109**	1.50E-07	***Nal1***
	LWSD	sf0710904892	7	10904892	**174**	7.05E-07	
	LWSD	sf0908837854	9	8837854	**214**	3.05E-07	
	LWSD	sf1004092248	10	4092248	**231**	1.08E-07	
	MLA	sf0428761252	4	28761252	**108**	4.44E-07	

MLA	sf0432486190	4	32486190	**113**	8.59E-07
MLA	sf1201620959	12	1620959	**268**	6.46E-08
MLC	sf0611654447	6	11654447	**149**	1.89E-07
MLC	sf0619842359	6	19842359	**157**	4.47E-07
MLC	sf1123693654	11	23693654	**259**	8.41E-07
MLL	sf0428761252	4	28761252	**108**	3.79E-07
MLL	sf0428785545	4	28785545	**108**	2.42E-07
MLL	sf0507207568	5	7207568	**120**	1.09E-06
MLP	sf0725046926	7	25046926	**185**	8.81E-07
MLP	sf0817786871	8	17786871	**198**	1.74E-07
MLRA	sf0428761252	4	28761252	**108**	8.01E-07
MLRA	sf0432485428	4	32485428	**113**	1.06E-06
MLW	sf0234524591	2	34524591	**55**	7.02E-08
MLW	sf0705098417	7	5098417	**171**	9.06E-07
MLWR	sf0133036641	1	33036641	**18**	2.26E-07
MLWR	sf0219182078	2	19182078	**44**	6.15E-07
MLWR	sf0231852071	2	31852071	**52**	8.16E-07
MLWR	sf0504419821	5	4419821	**119**	9.28E-07
MLWR	sf0908384958	9	8384958	**212**	1.09E-06
MLWR	sf1126653142	11	26653142	**262**	7.14E-07
MLWR	sf1208137510	12	8137510	**274**	1.09E-06
MPAR	sf0233399868	2	33399868	**54**	1.11E-06
MPAR	sf0408184910	4	8184910	**95**	1.04E-06
MPAR	sf0619381533	6	19381533	**156**	1.59E-08
MPAR	sf0819065928	8	19065928	**199**	4.56E-07
TLA	sf0431688161	4	31688161	**111**	1.27E-08
TLA	sf0908170004	9	8170004	**212**	1.09E-06
TLA	sf1122295601	11	22295601	**258**	1.04E-06
ALA	sf0128560533	1	28560533	**13**	3.40E-07
ALA	sf0141828134	1	41828134	**29**	8.87E-07
ALA	sf0141922044	1	41922044	**29**	4.96E-07
ALA	sf0232313257	2	32313257	**53**	1.16E-06
ALA	sf0327446419	3	27446419	**78**	2.66E-07
ALA	sf0709411773	7	9411773	**174**	1.17E-06
ALA	sf0724555302	7	24555302	**184**	1.20E-06
ALA	sf0800666984	8	666984	**188**	1.74E-07
ALA	sf0814391407	8	14391407	**195**	4.00E-07
ALA	sf0821777242	8	21777242	**201**	8.17E-08
ALA	sf1116966287	11	16966287	**254**	7.53E-07
ALA	sf1123946800	11	23946800	**259**	1.14E-06
ALA	sf1125058496	11	25058496	**260**	2.15E-07
ALC	sf0141345886	1	41345886	**28**	6.71E-07
ALL	sf0137055366	1	37055366	**20**	8.42E-08
ALL	sf0139583671	1	39583671	**26**	2.06E-07
ALL	sf0428557370	4	28557370	**108**	3.71E-07
ALL	sf1120033906	11	20033906	**257**	4.31E-07
ALL	sf1206312802	12	6312802	**273**	3.64E-07
ALL	sf1226297825	12	26297825	**286**	2.91E-07
ALP	sf0137055366	1	37055366	**20**	1.37E-07
ALP	sf0139583671	1	39583671	**26**	2.84E-07
ALP	sf0232479524	2	32479524	**53**	1.11E-06
ALP	sf0428557370	4	28557370	**108**	7.12E-07
ALP	sf1120033906	11	20033906	**257**	5.50E-07
ALP	sf1206312802	12	6312802	**273**	2.72E-07
ALP	sf1226297825	12	26297825	**286**	4.62E-07
ALRA	sf0128560533	1	28560533	**13**	3.17E-07
ALRA	sf0141828134	1	41828134	**29**	5.56E-07
ALRA	sf0141922044	1	41922044	**29**	4.76E-07
ALRA	sf0232313257	2	32313257	**53**	2.93E-07
ALRA	sf0326927102	3	26927102	**77**	6.44E-07
ALRA	sf0327446419	3	27446419	**78**	1.12E-07
ALRA	sf0431428481	4	31428481	**110**	9.89E-07

ALRA	sf0800666984	8	666984	**188**	2.32E-07
ALRA	sf0814391407	8	14391407	**195**	4.72E-07
ALRA	sf0821777242	8	21777242	**201**	4.74E-08
ALRA	sf1116966287	11	16966287	**254**	8.68E-07
ALRA	sf1125058496	11	25058496	**260**	1.19E-07
ALRA	sf1200777105	12	777105	**267**	7.39E-07
ALW	sf0108185933	1	8185933	**2**	7.45E-07
ALW	sf0114683953	1	14683953	**5**	1.04E-07
ALW	sf0141826241	1	41826241	**29**	2.30E-07
ALW	sf0141995597	1	41995597	**29**	3.93E-07
ALW	sf0214044855	2	14044855	**42**	1.04E-06
ALW	sf0413658525	4	13658525	**99**	7.30E-07
ALW	sf0724264482	7	24264482	**183**	1.07E-06
ALW	sf0821739186	8	21739186	**201**	5.17E-08
ALW	sf0824083286	8	24083286	**203**	7.74E-07
ALW	sf1227298521	12	27298521	**287**	7.52E-08
ALWR	sf0114214825	1	14214825	**4**	6.31E-07
ALWR	sf0114674675	1	14674675	**5**	9.39E-11
ALWR	sf0114715211	1	14715211	**5**	3.36E-07
ALWR	sf0114765895	1	14765895	**5**	1.52E-07
ALWR	sf0201313239	2	1313239	**30**	2.64E-07
ALWR	sf0214044855	2	14044855	**42**	3.88E-08
ALWR	sf0234971267	2	34971267	**56**	9.55E-08
ALWR	sf0305756733	3	5756733	**62**	1.33E-07
ALWR	sf0307967747	3	7967747	**66**	1.03E-08
ALWR	sf0314218985	3	14218985	**69**	3.12E-07
ALWR	sf0314311418	3	14311418	**69**	9.08E-07
ALWR	sf0335427520	3	35427520	**84**	2.15E-07
ALWR	sf0413658525	4	13658525	**99**	4.23E-07
ALWR	sf0421128220	4	21128220	**105**	2.42E-07
ALWR	sf0503041512	5	3041512	**118**	3.60E-07
ALWR	sf0510154318	5	10154318	**125**	1.00E-07
ALWR	sf0525702444	5	25702444	**136**	5.33E-07
ALWR	sf0609280147	6	9280147	**147**	1.13E-06
ALWR	sf0619264960	6	19264960	**156**	3.48E-07
ALWR	sf0700383598	7	383598	**166**	1.13E-06
ALWR	sf0705652347	7	5652347	**172**	4.41E-08
ALWR	sf0719010486	7	19010486	**179**	3.39E-07
ALWR	sf0825631964	8	25631964	**205**	1.10E-06
ALWR	sf0907139771	9	7139771	**211**	1.01E-06
ALWR	sf1108434098	11	8434098	**251**	2.78E-07
ALWR	sf1127347416	11	27347416	**264**	1.19E-06
ALWR	sf1128403654	11	28403654	**265**	1.12E-06
ALWR	sf1227298521	12	27298521	**287**	4.56E-09
APAR	sf0108185933	1	8185933	**2**	3.84E-08
APAR	sf0114765895	1	14765895	**5**	4.21E-07
APAR	sf0114790584	1	14790584	**5**	4.27E-12
APAR	sf0119419292	1	19419292	**9**	1.15E-06
APAR	sf0121978536	1	21978536	**10**	2.32E-07
APAR	sf0127810782	1	27810782	**12**	7.66E-07
APAR	sf0141826241	1	41826241	**29**	2.03E-08
APAR	sf0201313239	2	1313239	**30**	5.76E-09
APAR	sf0214044855	2	14044855	**42**	1.77E-09
APAR	sf0234971267	2	34971267	**56**	3.19E-07
APAR	sf0305756733	3	5756733	**62**	2.71E-10
APAR	sf0307967747	3	7967747	**66**	4.93E-10
APAR	sf0314041177	3	14041177	**69**	6.97E-07
APAR	sf0325170818	3	25170818	**76**	7.66E-07
APAR	sf0330767955	3	30767955	**80**	2.92E-07
APAR	sf0335429346	3	35429346	**84**	1.15E-06
APAR	sf0401073573	4	1073573	**85**	1.86E-07
APAR	sf0401199801	4	1199801	**85**	5.17E-08
APAR	sf0413658525	4	13658525	**99**	8.11E-10

	APAR	sf0431039801	4	31039801	**109**	5.54E-09	***Nal1***
	APAR	sf0526059024	5	26059024	**137**	6.11E-08	
	APAR	sf0604222011	6	4222011	**143**	1.16E-06	
	APAR	sf0611183488	6	11183488	**148**	4.82E-07	
	APAR	sf0613003646	6	13003646	**151**	2.79E-07	
	APAR	sf0705652347	7	5652347	**172**	2.77E-08	
	APAR	sf0708103740	7	8103740	**173**	3.96E-07	
	APAR	sf0724264482	7	24264482	**183**	1.66E-07	
	APAR	sf0813480582	8	13480582	**194**	7.64E-07	
	APAR	sf0821739186	8	21739186	**201**	6.33E-08	
	APAR	sf0917719915	9	17719915	**222**	3.27E-07	
	APAR	sf1102574411	11	2574411	**243**	1.62E-08	
	APAR	sf1106377393	11	6377393	**247**	1.12E-07	
	APAR	sf1127106642	11	27106642	**263**	2.41E-07	
	APAR	sf1200794496	12	794496	**267**	1.00E-06	
	APAR	sf1227298521	12	27298521	**287**	1.09E-10	
	GLA	sf0225036945	2	25036945	**48**	3.15E-07	
	GLA	sf0502443333	5	2443333	**117**	9.30E-07	
	GLA	sf0528506264	5	28506264	**138**	1.50E-07	
	GLA$_2$	sf1218880005	12	18880005	**281**	2.16E-07	
	GLA$_2$	sf1219454172	12	19454172	**282**	1.02E-06	
	GLA$_3$	sf0216114686	2	16114686	**43**	1.08E-06	
	GLA$_3$	sf0225036945	2	25036945	**48**	6.39E-08	
	GLA$_3$	sf0502443822	5	2443822	**117**	3.97E-07	
	GLA$_3$	sf0528506264	5	28506264	**138**	1.69E-07	
	GLA$_3$	sf0919753569	9	19753569	**224**	5.37E-07	
	GLA$_4$	sf0211842901	2	11842901	**40**	3.54E-07	
	GLA$_4$	sf0219198685	2	19198685	**44**	1.49E-07	
	GLA$_4$	sf0224586397	2	24586397	**47**	2.80E-07	
	GLA$_4$	sf0224824076	2	24824076	**47**	3.49E-08	
	GLA$_4$	sf0401986876	4	1986876	**86**	4.31E-08	
	GLA$_4$	sf0407787724	4	7787724	**94**	1.73E-07	
	GLA$_4$	sf0414776838	4	14776838	**100**	4.47E-07	
	GLA$_4$	sf0608186828	6	8186828	**146**	1.04E-07	
	GLA$_4$	sf0622511858	6	22511858	**159**	1.25E-08	
	GLA$_4$	sf0701026438	7	1026438	**168**	5.89E-09	
	GLA$_4$	sf0701170356	7	1170356	**168**	4.79E-07	
	GLA$_4$	sf0816934373	8	16934373	**197**	4.04E-07	
**Milk Grain stage**	*GLA*$_4$	sf0828043934	8	28043934	**208**	4.03E-07	
	GLA$_4$	sf1002331172	10	2331172	**229**	9.35E-09	
	GLA$_4$	sf1106820100	11	6820100	**248**	9.02E-08	
	GLA$_4$	sf1203270291	12	3270291	**270**	1.85E-07	
	GLA$_4$	sf1204827025	12	4827025	**272**	1.12E-06	
	GLAR	sf0109768408	1	9768408	**3**	6.75E-07	
	GLAR	sf0138139266	1	38139266	**22**	1.18E-07	
	GLAR	sf0234629350	2	34629350	**55**	6.63E-07	
	GLAR	sf0301144799	3	1144799	**59**	7.61E-08	
	GLAR	sf0301511588	3	1511588	**60**	7.31E-08	
	GLAR	sf0412542410	4	12542410	**98**	1.03E-07	
	GLAR	sf0523183599	5	23183599	**135**	4.82E-07	
	GLAR	sf0615328125	6	15328125	**153**	5.24E-07	
	GLAR	sf0626208873	6	26208873	**162**	1.61E-07	
	GLAR	sf0700598680	7	598680	**167**	6.72E-07	
	GLAR	sf0700843455	7	843455	**167**	1.07E-06	
	GLAR	sf0801713963	8	1713963	**189**	1.86E-07	
	GLAR	sf0817690033	8	17690033	**198**	1.80E-07	
	GLAR	sf0817823673	8	17823673	**198**	2.22E-08	
	GLAR	sf1015417171	10	15417171	**236**	3.10E-07	
	GLAR	sf1107334672	11	7334672	**249**	2.52E-07	
	GLAR$_2$	sf0418833729	4	18833729	**102**	1.00E-06	
	GLAR$_2$	sf0507107863	5	7107863	**120**	2.39E-07	
	GLAR$_3$	sf0507107863	5	7107863	**120**	2.60E-07	

$GLAR_3$	sf0603243009	6	3243009	**142**	5.40E-08	
$GLAR_4$	sf0122612616	1	22612616	**11**	3.78E-07	
$GLAR_4$	sf0137777974	1	37777974	**21**	4.04E-09	
$GLAR_4$	sf0204800916	2	4800916	**33**	9.14E-07	
$GLAR_4$	sf0207792232	2	7792232	**36**	2.17E-07	
$GLAR_4$	sf0216141744	2	16141744	**43**	1.04E-06	
$GLAR_4$	sf0227136932	2	27136932	**50**	8.56E-07	
$GLAR_4$	sf0401930451	4	1930451	**86**	3.15E-08	
$GLAR_4$	sf0405015647	4	5015647	**91**	3.34E-08	
$GLAR_4$	sf0420154357	4	20154357	**104**	4.39E-07	
$GLAR_4$	sf0433609350	4	33609350	**114**	2.59E-08	
$GLAR_4$	sf0529691765	5	29691765	**141**	3.33E-08	
$GLAR_4$	sf0608270810	6	8270810	**146**	7.97E-10	
$GLAR_4$	sf0620800068	6	20800068	**158**	3.52E-08	
$GLAR_4$	sf0622496123	6	22496123	**159**	1.21E-06	
$GLAR_4$	sf0622511851	6	22511851	**159**	2.09E-11	
$GLAR_4$	sf0625540899	6	25540899	**161**	1.06E-07	
$GLAR_4$	sf0704244483	7	4244483	**170**	4.92E-10	
$GLAR_4$	sf0816934373	8	16934373	**197**	2.45E-07	
$GLAR_4$	sf0825307574	8	25307574	**204**	6.13E-08	
$GLAR_4$	sf0919250139	9	19250139	**223**	1.33E-07	
$GLAR_4$	sf1003001590	10	3001590	**230**	3.05E-08	
$GLAR_4$	sf1017644195	10	17644195	**237**	8.83E-08	
$GLAR_4$	sf1203637332	12	3637332	**271**	5.32E-07	
$GLAR_4$	sf1212008851	12	12008851	**277**	2.49E-08	
GLCC	sf0327935961	3	27935961	**79**	2.75E-07	
GLCC	sf0401731355	4	1731355	**86**	1.75E-07	
GLCC	sf0402415033	4	2415033	**88**	4.79E-07	
GLCC	sf0529691765	5	29691765	**141**	3.15E-07	
GLCC	sf0622511851	6	22511851	**159**	8.93E-07	
LLSD	sf0814027825	8	14027825	**290**	8.87E-08	
LLSD	sf0912562314	9	12562314	**216**	5.19E-07	
LLSD	sf1121880654	11	21880654	**291**	8.31E-07	
LN	sf0211842901	2	11842901	**40**	5.46E-07	
LN	sf0608228082	6	8228082	**146**	4.05E-07	
LN	sf0608233257	6	8233257	**146**	1.01E-06	
LN	sf0716469200	7	16469200	**177**	7.09E-08	
LN	sf0820734571	8	20734571	**200**	9.29E-07	
LWSD	sf0117769123	1	17769123	**7**	1.21E-06	
LWSD	sf0225081784	2	25081784	**48**	1.95E-07	
LWSD	sf0318071699	3	18071699	**71**	1.09E-06	
LWSD	sf0332115908	3	32115908	**81**	4.43E-07	***OSH43***
LWSD	sf0422737268	4	22737268	**106**	9.41E-07	
LWSD	sf0427563863	4	27563863	**107**	5.43E-10	
LWSD	sf0520774778	5	20774778	**133**	8.42E-07	
LWSD	sf0625269478	6	25269478	**161**	4.73E-07	
LWSD	sf0626208873	6	26208873	**162**	6.12E-07	
LWSD	sf0630219773	6	30219773	**163**	7.92E-08	
LWSD	sf0702345742	7	2345742	**169**	1.40E-08	
LWSD	sf0718598941	7	18598941	**178**	7.39E-07	
LWSD	sf0719554750	7	19554750	**180**	1.98E-08	
LWSD	sf0815540826	8	15540826	**196**	4.47E-07	
LWSD	sf0908334292	9	8334292	**212**	9.20E-07	
LWSD	sf0911267191	9	11267191	**215**	8.07E-07	
LWSD	sf1012820814	10	12820814	**235**	8.32E-07	
LWSD	sf1020434982	10	20434982	**239**	1.17E-06	
LWSD	sf1100412783	11	412783	**242**	2.48E-07	
LWSD	sf1112834088	11	12834088	**252**	1.92E-07	
LWSD	sf1126108571	11	26108571	**261**	3.17E-08	
LWSD	sf1200141009	12	141009	**266**	4.08E-07	***NAL3***
LWSD	sf1204927416	12	4927416	**272**	1.86E-07	
LWSD	sf0116862977	1	16862977	**288**	1.11E-06	

MLA	sf0402013312	4	2013312	**86**	2.59E-09	
MLA	sf0402535451	4	2535451	**88**	2.49E-07	
MLA	sf0402885417	4	2885417	**89**	1.23E-07	
MLA	sf0404373124	4	4373124	**90**	2.64E-07	
MLA	sf0431688816	4	31688816	**111**	3.74E-07	
MLA	sf0431731166	4	31731166	**111**	7.55E-07	
MLA	sf0620743996	6	20743996	**158**	7.17E-07	
MLA	sf0710949944	7	10949944	**174**	1.07E-06	
MLA	sf0720571685	7	20571685	**182**	9.39E-07	
MLA	sf0724561303	7	24561303	**184**	1.08E-07	
MLA	sf0908518957	9	8518957	**213**	1.08E-06	
MLA	sf0912562314	9	12562314	**216**	4.35E-08	
MLA	sf1001305024	10	1305024	**228**	8.92E-08	
MLA	sf1100512380	11	512380	**242**	5.44E-07	
MLA	sf1117763526	11	17763526	**255**	5.84E-07	
MLA	sf1208135880	12	8135880	**274**	9.59E-07	
MLA	sf1217534035	12	17534035	**280**	6.20E-07	
MLC	sf0108340715	1	8340715	**2**	6.26E-07	
MLC	sf0219191723	2	19191723	**44**	1.14E-06	
MLC	sf0224656597	2	24656597	**47**	6.49E-08	
MLC	sf0509638422	5	9638422	**124**	7.77E-07	
MLC	sf0614927674	6	14927674	**152**	1.96E-08	
MLC	sf0623142861	6	23142861	**160**	9.32E-07	
MLC	sf0700595552	7	595552	**167**	1.05E-06	
MLL	sf0137065930	1	37065930	**20**	1.28E-07	
MLL	sf0431177805	4	31177805	**109**	3.84E-07	***Nal1***
MLP	sf0319496982	3	19496982	**72**	1.19E-06	
MLP	sf0912562314	9	12562314	**216**	8.03E-07	
MLP	sf0919274354	9	19274354	**223**	1.04E-06	
MLP	sf1117763526	11	17763526	**255**	9.07E-08	
MLRA	sf0805320783	8	5320783	**191**	1.98E-07	
MLRA	sf0912562314	9	12562314	**216**	9.05E-08	
MLRA	sf1125096468	11	25096468	**260**	6.61E-07	
MLW	sf0307235728	3	7235728	**65**	5.41E-07	
MLW	sf0327165381	3	27165381	**77**	7.52E-07	
MLW	sf0805320783	8	5320783	**191**	9.19E-07	
MLWR	sf0130150859	1	30150859	**15**	3.47E-07	
MLWR	sf0138432053	1	38432053	**23**	2.35E-08	
MLWR	sf0219185409	2	19185409	**44**	1.74E-08	
MLWR	sf0508177876	5	8177876	**121**	4.59E-07	
MLWR	sf0508616303	5	8616303	**122**	2.74E-07	
MLWR	sf0509154430	5	9154430	**123**	4.05E-07	
MPAR	sf0141826241	1	41826241	**29**	8.32E-07	
MPAR	sf0306154166	3	6154166	**63**	2.95E-07	
MPAR	sf0431039801	4	31039801	**109**	1.11E-06	***Nal1***
MPAR	sf0715963842	7	15963842	**176**	6.70E-07	
MPAR	sf0824096072	8	24096072	**203**	2.82E-07	
MPAR	sf1227298521	12	27298521	**287**	9.69E-08	
TLA	sf0225036945	2	25036945	**48**	4.22E-07	
TLA	sf0528506264	5	28506264	**138**	7.30E-08	

Supplementary table S4 GWAS results with the significant *P* value threshold

Stage	Trait	Lead SNP ID	Chr.	Position (MSU6.1)	Locus ID	*P* value	Reference allele	Major allele	Minor allele	R^2 (%)[a]	Effect size (s.d.)[b]	Known genes
Late tillering stage	GLA_2	sf0114653019	1	14653019	**5**	9.61E-09	T	T	C	6.80	1.01	
	GLA_2	sf0137212688	1	37212688	**20**	1.06E-08	T	G	T	7.26	-1.41	
	GLA_2	sf0207484769	2	7484769	**35**	8.12E-09	G	G	A	7.12	1.33	
	GLA_2	sf0331940902	3	31940902	**81**	7.56E-09	T	C	T	6.84	-1.81	
	GLA_2	sf0313954229	3	13954229	**69**	5.55E-08	G	A	G	6.21	-1.07	
	GLA_2	sf0305695371	3	5695371	**62**	4.50E-08	G	T	G	5.96	-0.87	
	GLA_2	sf0406027943	4	6027943	**93**	2.94E-10	C	C	T	8.16	1.04	
	GLA_2	sf0516433687	5	16433687	**130**	1.76E-09	T	T	A	7.42	1.28	
	GLA_2	sf0525748215	5	25748215	**136**	8.10E-09	T	T	G	6.86	2.36	
	GLA_2	sf0502421097	5	2421097	**117**	2.89E-09	G	G	A	7.83	0.93	
	GLA_2	sf0513728565	5	13728565	**126**	5.48E-12	C	C	T	10.06	1.59	
	GLA_2	sf0630583038	6	30583038	**164**	4.66E-08	C	C	T	6.00	1.07	
	GLA_2	sf0700219702	7	219702	**166**	4.36E-09	G	G	T	5.88	1.07	
	GLA_2	sf0711200104	7	11200104	**174**	1.43E-09	A	C	A	7.70	-0.76	
	GLA_2	sf0826476003	8	26476003	**206**	1.47E-10	G	G	A	8.56	0.99	
	GLA_2	sf0804368745	8	4368745	**190**	3.05E-09	A	T	A	7.10	-1.27	
	GLA_2	sf0905443985	9	5443985	**210**	1.44E-08	C	C	T	6.74	0.92	
	GLA_2	sf1022775806	10	22775806	**241**	2.23E-09	C	C	T	7.07	1.11	
	GLA_2	sf1126753044	11	26753044	**262**	1.73E-08	C	C	T	8.18	1.12	
	GLA_2	sf1215668235	12	15668235	**279**	1.21E-08	C	C	T	7.02	0.96	
	GLA_2	sf1224620960	12	24620960	**284**	4.50E-09	C	C	A	7.46	1.06	
	GLA_2	sf1202270243	12	2270243	**269**	7.19E-09	C	T	C	7.15	-0.89	
	GLA_3	sf0514281006	5	14281006	**127**	5.20E-08	A	A	G	6.83	1.06	
	GLAR	sf0129629431	1	29629431	**14**	2.14E-10	G	G	A	8.23	2.22	
	GLAR	sf0323995809	3	23995809	**74**	1.31E-09	G	G	T	7.58	1.92	
	GLAR	sf0616715458	6	16715458	**154**	4.60E-08	T	T	C	2.71	1.22	
	GLAR	sf1106112328	11	6112328	**246**	2.48E-08	G	G	A	6.46	2.02	
	$GLAR_3$	sf0529205395	5	29205395	**140**	2.65E-08	C	C	T	6.58	1.15	
	$GLAR_3$	sf0708241028	7	8241028	**173**	1.62E-08	T	T	C	6.85	1.05	
	$GLAR_3$	sf0719639267	7	19639267	**180**	1.94E-13	T	C	T	11.23	-1.35	
	$GLAR_3$	sf0719817763	7	19817763	**180**	6.19E-11	G	G	T	9.00	-1.25	
	$GLAR_3$	sf0719728350	7	19728350	**180**	3.62E-08	G	G	A	7.32	-0.66	

	GLAR$_3$	sf0719933345	7	19933345	**181**	5.54E-08	T	T	C	6.29	-0.69	
	GLAR$_3$	sf1117150282	11	17150282	**254**	2.07E-10	C	C	T	8.50	1.33	
	GLAR$_4$	sf0719859898	7	19859898	**181**	8.10E-11	T	C	T	8.93	1.01	
	GLAR$_4$	sf0719732764	7	19732764	**180**	1.02E-08	C	C	T	7.00	0.84	
	GLCC	sf0719639464	7	19639464	**180**	4.43E-09	T	C	T	7.31	1.08	
	LLSD	sf1011328985	10	11328985	**233**	3.25E-08	T	C	T	6.48	1.09	
	LWSD	sf0203791234	2	3791234	**31**	6.91E-09	A	A	G	3.15	1.38	
	LWSD	sf0514011442	5	14011442	**127**	1.27E-08	C	C	A	3.44	1.69	
	LWSD	sf0528903428	5	28903428	**139**	4.99E-09	G	G	A	4.12	1.88	
	LWSD	sf0606820810	6	6820810	**144**	8.94E-10	C	C	T	5.16	3.09	
	LWSD	sf1127580568	11	27580568	**264**	4.61E-09	A	A	T	6.68	1.17	
	MLW	sf1105896366	11	5896366	**246**	4.41E-08	C	C	A	6.34	0.72	
	ALL	sf1225286279	12	25286279	**285**	5.54E-08	G	G	A	6.20	-0.69	***OsIAA3***
	ALL	sf1225260606	12	25260606	**285**	4.31E-09	A	T	A	7.25	-0.74	***OsIAA3***
	ALP	sf1225286279	12	25286279	**285**	4.68E-08	G	G	A	6.23	-0.69	***OsIAA3***
	ALP	sf1225260606	12	25260606	**285**	6.09E-09	A	T	A	7.05	-0.74	***OsIAA3***
	ALWR	sf0402803386	4	2803386	**89**	4.71E-09	A	A	G	7.03	0.71	
	ALWR	sf0727101845	7	27101845	**187**	2.72E-08	C	C	A	6.33	-1.15	
**Late booting stage**	GLA	sf0431688161	4	31688161	**111**	1.52E-08	C	C	T	6.94	0.98	
	GLA$_2$	sf0620927842	6	20927842	**158**	1.52E-08	G	G	A	6.17	1.05	
	GLAR	sf0216062250	2	16062250	**43**	3.46E-08	T	C	T	6.22	-1.46	
	GLAR$_4$	sf0620856382	6	20856382	**158**	6.95E-09	A	G	A	6.72	-1.02	
	LN	sf0431688161	4	31688161	**111**	6.07E-09	C	C	T	7.20	0.99	
	MPAR	sf0619381533	6	19381533	**156**	1.59E-08	C	C	T	5.70	0.92	
	TLA	sf0431688161	4	31688161	**111**	1.27E-08	C	C	T	7.00	0.98	
	ALRA	sf0821777242	8	21777242	**201**	4.74E-08	T	C	T	7.41	1.14	
	ALW	sf0821739186	8	21739186	**201**	5.17E-08	T	C	T	7.26	1.19	
	ALWR	sf0114674675	1	14674675	**5**	9.39E-11	A	G	A	10.37	1.43	
	ALWR	sf0214044855	2	14044855	**42**	3.88E-08	G	G	A	7.74	-2.16	
	ALWR	sf0307967747	3	7967747	**66**	1.03E-08	C	C	T	8.27	-2.15	
	ALWR	sf0705652347	7	5652347	**172**	4.41E-08	A	A	T	7.47	-2.29	
	ALWR	sf1227298521	12	27298521	**287**	4.56E-09	A	A	G	8.56	1.21	
	APAR	sf0108185933	1	8185933	**2**	3.84E-08	G	G	T	7.61	-2.28	
	APAR	sf0141826241	1	41826241	**29**	2.03E-08	A	C	A	7.49	-0.91	
	APAR	sf0114790584	1	14790584	**5**	4.27E-12	T	C	T	11.74	1.61	
	APAR	sf0201313239	2	1313239	**30**	5.76E-09	G	G	T	8.40	-2.52	

**Milk Grain stage**	APAR	sf0214044855	2	14044855	**42**	1.77E-09	G	G	A	9.05	-2.44	
	APAR	sf0307967747	3	7967747	**66**	4.93E-10	C	C	T	9.79	-2.46	
	APAR	sf0305756733	3	5756733	**62**	2.71E-10	T	C	T	10.14	2.06	
	APAR	sf0413658525	4	13658525	**99**	8.11E-10	T	T	C	9.37	-2.88	
	APAR	sf0431039801	4	31039801	**109**	5.54E-09	A	A	G	8.19	1.33	***Nal1***
	APAR	sf0401199801	4	1199801	**85**	5.17E-08	A	A	T	7.16	0.99	
	APAR	sf0705652347	7	5652347	**172**	2.77E-08	A	A	T	7.69	-2.42	
	APAR	sf1102574411	11	2574411	**243**	1.62E-08	T	C	T	7.81	-1.41	
	APAR	sf1227298521	12	27298521	**287**	1.09E-10	A	A	G	10.47	1.41	
	GLA$_4$	sf0224824076	2	24824076	**47**	3.49E-08	C	C	T	7.56	1.39	
	GLA$_4$	sf0401986876	4	1986876	**86**	4.31E-08	C	A	C	7.44	-1.07	
	GLA$_4$	sf0622511858	6	22511858	**159**	1.25E-08	T	C	T	8.88	-1.16	
	GLA$_4$	sf0701026438	7	1026438	**168**	5.89E-09	T	C	T	8.32	-1.33	
	GLA4	sf1002331172	10	2331172	**229**	9.35E-09	G	G	C	8.21	1.24	
	GLAR	sf0817823673	8	17823673	**198**	2.22E-08	G	C	G	7.61	-0.91	
	GLAR$_3$	sf0603243009	6	3243009	**142**	5.40E-08	C	C	A	7.10	-0.80	
	GLAR$_4$	sf0137777974	1	37777974	**21**	4.04E-09	T	T	C	8.46	1.34	
	GLAR$_4$	sf0433609350	4	33609350	**114**	2.59E-08	T	C	T	7.61	-1.21	
	GLAR$_4$	sf0401930451	4	1930451	**86**	3.15E-08	A	T	A	7.40	-1.11	
	GLAR$_4$	sf0405015647	4	5015647	**91**	3.34E-08	A	G	A	7.58	-0.95	
	GLAR$_4$	sf0529691765	5	29691765	**141**	3.33E-08	A	T	A	7.45	-1.11	
	GLAR$_4$	sf0608270810	6	8270810	**146**	7.97E-10	A	G	A	9.24	-1.27	
	GLAR$_4$	sf0622511851	6	22511851	**159**	2.09E-11	T	C	T	10.91	-1.18	
	GLAR$_4$	sf0620800068	6	20800068	**158**	3.52E-08	T	C	T	7.49	-1.07	
	GLAR$_4$	sf0704244483	7	4244483	**170**	4.92E-10	C	C	T	9.46	1.40	
	GLAR4	sf1003001590	10	3001590	**230**	3.05E-08	A	G	A	6.94	-1.15	
	GLAR$_4$	sf1212008851	12	12008851	**277**	2.49E-08	G	C	G	7.08	-1.06	
	LWSD	sf0427563863	4	27563863	**107**	5.43E-10	A	A	G	9.18	-1.19	
	LWSD	sf0719554750	7	19554750	**180**	1.98E-08	G	G	A	7.82	1.48	
	LWSD	sf0702345742	7	2345742	**169**	1.40E-08	T	T	C	6.75	1.78	
	LWSD	sf1126108571	11	26108571	**261**	3.17E-08	T	T	C	6.01	1.04	
	MLA	sf0402013312	4	2013312	**86**	2.59E-09	C	C	T	8.75	1.43	
	MLA	sf0912562314	9	12562314	**216**	4.35E-08	C	C	T	7.40	1.23	
	MLC	sf0614927674	6	14927674	**152**	1.96E-08	C	C	T	6.92	2.00	
	MLWR	sf0138432053	1	38432053	**23**	2.35E-08	C	C	T	7.67	-0.88	
	MLWR	sf0219185409	2	19185409	**44**	1.74E-08	G	G	A	7.84	0.69	

[a]R^2 (%), proportion of variation explained by a SNP, calculated as SS of a lead SNP (after fitting all other model terms) / SS of total, where SS stands for sum of squares.

[b]Effect size is expressed as the ratio of absolute effect size / corresponding phenotypic standard deviation

Supplementary table S5 All the potential candidate genes within 100-kb upstream and downstream of the lead SNP sf0719639267.

Start	End	Annotation	LOC
19540488	19544813	DNA repair protein rhp16__putative__expressed;	LOC_Os07g32730
19560051	19562113	transposon protein__putative__CACTA__En/Spm sub-class;	LOC_Os07g32740
19566706	19574595	transposon protein__putative__CACTA__En/Spm sub-class;	LOC_Os07g32750
19592349	19594226	hypothetical protein;	LOC_Os07g32761
19597514	19608052	chorismate mutase/prephenate dehydratase__putative__expressed;	LOC_Os07g32774
19613248	19616481	ankyrin repeat domain containing protein__expressed;	LOC_Os07g32790
19624532	19627300	autophagy-related protein__putative__expressed;	LOC_Os07g32800
19630213	19630689	conserved hypothetical protein;	LOC_Os07g32810
19633395	19633709	hypothetical protein;	LOC_Os07g32820
19639898	19640377	hypothetical protein;	LOC_Os07g32830
19645851	19646315	hypothetical protein;	LOC_Os07g32839
19649497	19649985	hypothetical protein;	LOC_Os07g32850
19653850	19654365	hypothetical protein;	LOC_Os07g32870
19654998	19656610	ATP synthase gamma chain__putative__expressed;	LOC_Os07g32880
19657126	19667113	3'-5' exonuclease, putative, expressed	LOC_Os07g32890
19667385	19672215	PPR repeat containing protein__expressed;	LOC_Os07g32900
19673075	19679440	transposon protein__putative__unclassified__expressed;	LOC_Os07g32910
19681831	19683646	expressed protein;	LOC_Os07g32920
19689863	19690267	hypothetical protein;	LOC_Os07g32940
19691311	19694414	dnaJ C terminal region family protein__expressed;	LOC_Os07g32950
19695406	19695894	hypothetical protein;	LOC_Os07g32960
19697839	19698359	hypothetical protein;	LOC_Os07g32970
19702564	19703028	hypothetical protein;	LOC_Os07g32979
19708491	19708970	hypothetical protein;	LOC_Os07g32990
19712202	19715342	retrotransposon protein__putative__unclassified;	LOC_Os07g33000
19716466	19722621	retrotransposon protein__putative__unclassified;	LOC_Os07g33010
19726313	19727150	hypothetical protein;	LOC_Os07g33020
19730852	19731535	hypothetical protein;	LOC_Os07g33030
19735461	19735961	hypothetical protein;	LOC_Os07g33040

Supplementary Table S6 Association analysis of *Nal1* with ALWR at the late booting stage.

SNP	Chr	Position	*P* value	R^2 (%)*
sf0431027691	4	31027691	3.77E-04	2.77
sf0431020323	4	31020323	3.86E-02	0.93
sf0431029369	4	31029369	7.75E-02	0.68
sf0431018731	4	31018731	1.82E-01	0.40
sf0431027517	4	31027517	2.66E-01	0.27
sf0431020320	4	31020320	2.66E-01	0.27
sf0431019560	4	31019560	2.67E-01	0.27
sf0431027456	4	31027456	2.67E-01	0.27
sf0431029618	4	31029618	2.91E-01	0.24
sf0431028935	4	31028935	2.91E-01	0.24
sf0431028909	4	31028909	2.91E-01	0.24
sf0431027536	4	31027536	3.50E-01	0.19
sf0431028704	4	31028704	3.50E-01	0.19
sf0431019377	4	31019377	3.68E-01	0.18
sf0431019357	4	31019357	3.95E-01	0.16
sf0431029574	4	31029574	4.37E-01	0.13
sf0431026911	4	31026911	4.91E-01	0.10
sf0431029413	4	31029413	5.43E-01	0.08
sf0431027379	4	31027379	5.44E-01	0.08
sf0431019954	4	31019954	5.44E-01	0.08
sf0431018718	4	31018718	5.73E-01	0.07
sf0431027055	4	31027055	6.08E-01	0.06
sf0431020128	4	31020128	8.78E-01	0.01

*R^2 (%), proportion of variation explained by a SNP, calculated as SS of a lead SNP (after fitting all other model terms) / SS of total, where SS stands for sum of squares.

Supplementary Table S7 Variance explained by suggestive lead SNPs.

Trait	Group	Stage	Number of suggestive lead SNPs	Phenotypic variance explained (%)	Genetic variance explained (%)
$GLAR_4$	Color-related	milk grain stage	24	34.5	63.89
ALWR	shape-related	milk grain stage	28	53.9	58.59
ALA	size-related	milk grain stage	13	37.6	41.32
GLA_2	Color-related	ate tillering stag	55	80.4	87.39

Supplementary Table S8 The heritability (*h^2*) of 29 leaf traits at three growth stages.

Stage	Trait	Heritability
Late tillering stage	LN	0.80
	GLA	0.71
	GLA_2	0.92
	GLA_3	0.81
	GLA_4	0.75
	TLA	0.69
	GLAR	0.90
	$GLAR_2$	0.89
	$GLAR_3$	0.84
	$GLAR_4$	0.87
	GLCC	0.81
	LLSD	0.74
	LWSD	0.90
	ALL	0.91
	ALW	0.95
	ALRA	0.90
	ALWR	0.96
	ALP	0.90
	ALA	0.89
	APAR	0.96
	ALC	0.83
	MLL	0.88
	MLW	0.86
	MLRA	0.84
	MLWR	0.92
	MLP	0.81
	MLA	0.81
	MPAR	0.96
	MLC	0.66
Late booting stage	LN	0.86
	GLA	0.79
	GLA_2	0.84
	GLA_3	0.83
	GLA_4	0.80
	TLA	0.79
	GLAR	0.73
	$GLAR_2$	0.88
	$GLAR_3$	0.82
	$GLAR_4$	0.78
	GLCC	0.82
	LLSD	0.86
	LWSD	0.50
	ALL	0.94
	ALW	0.95
	ALRA	0.93
	ALWR	0.98
	ALP	0.94
	ALA	0.93

	APAR	0.97
	ALC	0.89
	MLL	0.89
	MLW	0.82
	MLRA	0.85
	MLWR	0.91
	MLP	0.86
	MLA	0.90
	MPAR	0.92
	MLC	0.75
**Milk grain stage**	LN	0.70
	GLA	0.68
	GLA_2	0.80
	GLA_3	0.81
	GLA_4	0.51
	TLA	0.69
	GLAR	0.88
	$GLAR_2$	0.82
	$GLAR_3$	0.82
	$GLAR_4$	0.54
	GLCC	0.72
	LLSD	0.80
	LWSD	0.85
	ALL	0.93
	ALW	0.94
	ALRA	0.92
	ALWR	0.92
	ALP	0.93
	ALA	0.91
	APAR	0.91
	ALC	0.83
	MLL	0.90
	MLW	0.84
	MLRA	0.86
	MLWR	0.90
	MLP	0.87
	MLA	0.88
	MPAR	0.89
	MLC	0.81

Supplementary Table S9 Epistasis analysis of significant lead SNPs.

SNP1	SNP2	P	Trait	stage
sf0700219702	sf0502421097	8.088e-16	GLA_2	late tillering stage
sf0700219702	sf0804368745	1.396e-12	GLA_2	late tillering stage
sf0700219702	sf0905443985	3.916e-10	GLA_2	late tillering stage
sf0700219702	sf0305695371	7.677e-24	GLA_2	late tillering stage
sf0700219702	sf0207484769	6.835e-11	GLA_2	late tillering stage
sf0700219702	sf0711200104	6.066e-39	GLA_2	late tillering stage
sf0700219702	sf0513728565	1.336e-24	GLA_2	late tillering stage
sf0700219702	sf0313954229	3.569e-40	GLA_2	late tillering stage
sf0700219702	sf0516433687	1.753e-13	GLA_2	late tillering stage
sf0700219702	sf0525748215	2.943e-17	GLA_2	late tillering stage
sf0700219702	sf0826476003	3.467e-05	GLA_2	late tillering stage
sf0700219702	sf0331940902	5.059e-23	GLA_2	late tillering stage
sf0700219702	sf0137212688	1.272e-43	GLA_2	late tillering stage
sf0700219702	sf1022775806	1.155e-14	GLA_2	late tillering stage
sf0700219702	sf1202270243	1.04e-40	GLA_2	late tillering stage
sf0700219702	sf1215668235	5.128e-26	GLA_2	late tillering stage
sf0502421097	sf0804368745	5.972e-14	GLA_2	late tillering stage
sf0502421097	sf0905443985	2.455e-09	GLA_2	late tillering stage
sf0502421097	sf0305695371	8.431e-07	GLA_2	late tillering stage
sf0502421097	sf0711200104	1.716e-15	GLA_2	late tillering stage
sf0502421097	sf0513728565	1.489e-07	GLA_2	late tillering stage
sf0502421097	sf0313954229	4.535e-07	GLA_2	late tillering stage
sf0502421097	sf0516433687	6.678e-05	GLA_2	late tillering stage
sf0502421097	sf0826476003	2.374e-14	GLA_2	late tillering stage
sf0502421097	sf0137212688	4.999e-15	GLA_2	late tillering stage
sf0502421097	sf1202270243	3.195e-08	GLA_2	late tillering stage
sf0502421097	sf1215668235	4.528e-09	GLA_2	late tillering stage
sf0502421097	sf1224620960	4.959e-11	GLA_2	late tillering stage
sf0804368745	sf0905443985	1.638e-10	GLA_2	late tillering stage
sf0804368745	sf0305695371	8.15e-05	GLA_2	late tillering stage
sf0804368745	sf0406027943	1.655e-17	GLA_2	late tillering stage
sf0804368745	sf0207484769	3.296e-18	GLA_2	late tillering stage
sf0804368745	sf0513728565	2.731e-12	GLA_2	late tillering stage
sf0804368745	sf0516433687	1.561e-24	GLA_2	late tillering stage
sf0804368745	sf0525748215	3.544e-14	GLA_2	late tillering stage
sf0804368745	sf0630583038	1.698e-22	GLA_2	late tillering stage
sf0804368745	sf1022775806	1.831e-20	GLA_2	late tillering stage
sf0804368745	sf1126753044	6.29e-09	GLA_2	late tillering stage
sf0804368745	sf1215668235	2.136e-18	GLA_2	late tillering stage
sf0804368745	sf1224620960	8.5e-18	GLA_2	late tillering stage
sf0905443985	sf0305695371	5.328e-17	GLA_2	late tillering stage
sf0905443985	sf0406027943	8.232e-06	GLA_2	late tillering stage
sf0905443985	sf0711200104	7.869e-33	GLA_2	late tillering stage
sf0905443985	sf0513728565	6.799e-06	GLA_2	late tillering stage
sf0905443985	sf0313954229	1.221e-13	GLA_2	late tillering stage

sf0905443985	sf0516433687	6.304e-06	GLA_2	late tillering stage
sf0905443985	sf0525748215	5.558e-05	GLA_2	late tillering stage
sf0905443985	sf0826476003	8.364e-18	GLA_2	late tillering stage
sf0905443985	sf0630583038	7.75e-05	GLA_2	late tillering stage
sf0905443985	sf0331940902	2.817e-23	GLA_2	late tillering stage
sf0905443985	sf0137212688	1.44e-41	GLA_2	late tillering stage
sf0905443985	sf1022775806	6.73e-12	GLA_2	late tillering stage
sf0905443985	sf1202270243	9.506e-37	GLA_2	late tillering stage
sf0905443985	sf1215668235	3.428e-10	GLA_2	late tillering stage
sf0905443985	sf1224620960	3.099e-07	GLA_2	late tillering stage
sf0305695371	sf0406027943	5.25e-24	GLA_2	late tillering stage
sf0305695371	sf0516433687	5.035e-08	GLA_2	late tillering stage
sf0305695371	sf0630583038	2.839e-36	GLA_2	late tillering stage
sf0305695371	sf1022775806	2.95e-19	GLA_2	late tillering stage
sf0305695371	sf1126753044	8.503e-24	GLA_2	late tillering stage
sf0305695371	sf1215668235	5.931e-06	GLA_2	late tillering stage
sf0305695371	sf1224620960	1.24e-16	GLA_2	late tillering stage
sf0406027943	sf0207484769	3.635e-16	GLA_2	late tillering stage
sf0406027943	sf0711200104	3.319e-29	GLA_2	late tillering stage
sf0406027943	sf0513728565	2.544e-17	GLA_2	late tillering stage
sf0406027943	sf0313954229	4.92e-38	GLA_2	late tillering stage
sf0406027943	sf0516433687	3.444e-13	GLA_2	late tillering stage
sf0406027943	sf0525748215	2.713e-16	GLA_2	late tillering stage
sf0406027943	sf0826476003	4.34e-09	GLA_2	late tillering stage
sf0406027943	sf0331940902	1.625e-22	GLA_2	late tillering stage
sf0406027943	sf0137212688	5.675e-41	GLA_2	late tillering stage
sf0406027943	sf1202270243	8.976e-32	GLA_2	late tillering stage
sf0406027943	sf1215668235	2.958e-23	GLA_2	late tillering stage
sf0207484769	sf0711200104	5.007e-07	GLA_2	late tillering stage
sf0207484769	sf0313954229	1.29e-05	GLA_2	late tillering stage
sf0207484769	sf0630583038	2.744e-16	GLA_2	late tillering stage
sf0207484769	sf0331940902	2.012e-08	GLA_2	late tillering stage
sf0207484769	sf0137212688	4.855e-08	GLA_2	late tillering stage
sf0207484769	sf1022775806	4.585e-13	GLA_2	late tillering stage
sf0207484769	sf1126753044	4.343e-16	GLA_2	late tillering stage
sf0207484769	sf1215668235	1.05e-16	GLA_2	late tillering stage
sf0207484769	sf1224620960	2.462e-16	GLA_2	late tillering stage
sf0711200104	sf0513728565	1.328e-26	GLA_2	late tillering stage
sf0711200104	sf0516433687	5.346e-20	GLA_2	late tillering stage
sf0711200104	sf0525748215	9.45e-12	GLA_2	late tillering stage
sf0711200104	sf0826476003	8.062e-13	GLA_2	late tillering stage
sf0711200104	sf0630583038	1.003e-33	GLA_2	late tillering stage
sf0711200104	sf1022775806	2.112e-37	GLA_2	late tillering stage
sf0711200104	sf1126753044	3.782e-22	GLA_2	late tillering stage
sf0711200104	sf1215668235	4.195e-27	GLA_2	late tillering stage
sf0711200104	sf1224620960	7.759e-39	GLA_2	late tillering stage
sf0513728565	sf0313954229	5.827e-06	GLA_2	late tillering stage
sf0513728565	sf0516433687	9.329e-08	GLA_2	late tillering stage

sf0513728565	sf0826476003	2.762e-12	GLA_2	late tillering stage
sf0513728565	sf0630583038	1.176e-22	GLA_2	late tillering stage
sf0513728565	sf0331940902	1.95e-26	GLA_2	late tillering stage
sf0513728565	sf0137212688	1.192e-45	GLA_2	late tillering stage
sf0513728565	sf1022775806	3.782e-22	GLA_2	late tillering stage
sf0513728565	sf1126753044	6.546e-25	GLA_2	late tillering stage
sf0513728565	sf1202270243	7.396e-20	GLA_2	late tillering stage
sf0513728565	sf1215668235	8.233e-27	GLA_2	late tillering stage
sf0513728565	sf1224620960	3.128e-16	GLA_2	late tillering stage
sf0313954229	sf0525748215	4.929e-06	GLA_2	late tillering stage
sf0313954229	sf0630583038	1.428e-37	GLA_2	late tillering stage
sf0313954229	sf1022775806	4.579e-22	GLA_2	late tillering stage
sf0313954229	sf1126753044	8.938e-25	GLA_2	late tillering stage
sf0313954229	sf1215668235	9.5e-21	GLA_2	late tillering stage
sf0313954229	sf1224620960	3.653e-26	GLA_2	late tillering stage
sf0516433687	sf0525748215	4.815e-18	GLA_2	late tillering stage
sf0516433687	sf0826476003	7.36e-12	GLA_2	late tillering stage
sf0516433687	sf0630583038	6.022e-13	GLA_2	late tillering stage
sf0516433687	sf0331940902	2.666e-08	GLA_2	late tillering stage
sf0516433687	sf0137212688	6.886e-05	GLA_2	late tillering stage
sf0516433687	sf1022775806	3.904e-14	GLA_2	late tillering stage
sf0516433687	sf1126753044	1.619e-15	GLA_2	late tillering stage
sf0516433687	sf1202270243	1.301e-10	GLA_2	late tillering stage
sf0516433687	sf1215668235	1.723e-08	GLA_2	late tillering stage
sf0516433687	sf1224620960	1.669e-13	GLA_2	late tillering stage
sf0525748215	sf0826476003	6.431e-08	GLA_2	late tillering stage
sf0525748215	sf0630583038	1.91e-16	GLA_2	late tillering stage
sf0525748215	sf0331940902	6.115e-27	GLA_2	late tillering stage
sf0525748215	sf0137212688	1.898e-26	GLA_2	late tillering stage
sf0525748215	sf1022775806	1.466e-16	GLA_2	late tillering stage
sf0525748215	sf1126753044	2.913e-16	GLA_2	late tillering stage
sf0525748215	sf1202270243	1.417e-06	GLA_2	late tillering stage
sf0525748215	sf1215668235	6.802e-17	GLA_2	late tillering stage
sf0525748215	sf1224620960	1.659e-16	GLA_2	late tillering stage
sf0826476003	sf0630583038	3.014e-07	GLA_2	late tillering stage
sf0826476003	sf1022775806	8.513e-22	GLA_2	late tillering stage
sf0826476003	sf1126753044	9.784e-08	GLA_2	late tillering stage
sf0826476003	sf1215668235	2.842e-26	GLA_2	late tillering stage
sf0826476003	sf1224620960	1.412e-13	GLA_2	late tillering stage
sf0630583038	sf0331940902	2.397e-22	GLA_2	late tillering stage
sf0630583038	sf0137212688	7.783e-41	GLA_2	late tillering stage
sf0630583038	sf1202270243	1.01e-37	GLA_2	late tillering stage
sf0630583038	sf1215668235	2.633e-09	GLA_2	late tillering stage
sf0331940902	sf1022775806	1.268e-15	GLA_2	late tillering stage
sf0331940902	sf1126753044	1.726e-12	GLA_2	late tillering stage
sf0331940902	sf1215668235	4.245e-09	GLA_2	late tillering stage
sf0331940902	sf1224620960	6.369e-23	GLA_2	late tillering stage
sf0137212688	sf1022775806	7.928e-41	GLA_2	late tillering stage

sf0137212688	sf1126753044	2.745e-27	GLA_2	late tillering stage
sf0137212688	sf1215668235	1.44e-22	GLA_2	late tillering stage
sf0137212688	sf1224620960	1.1e-41	GLA_2	late tillering stage
sf1022775806	sf1202270243	1.151e-25	GLA_2	late tillering stage
sf1022775806	sf1215668235	8.45e-09	GLA_2	late tillering stage
sf1022775806	sf1224620960	8.905e-09	GLA_2	late tillering stage
sf1126753044	sf1202270243	1.03e-17	GLA_2	late tillering stage
sf1126753044	sf1215668235	4.314e-13	GLA_2	late tillering stage
sf1202270243	sf1215668235	3.885e-10	GLA_2	late tillering stage
sf1202270243	sf1224620960	5.666e-28	GLA_2	late tillering stage
sf1215668235	sf1224620960	1.791e-24	GLA_2	late tillering stage
sf0606820810	sf1127580568	1.193e-10	LWSD	late tillering stage
sf0514011442	sf1127580568	1.606e-10	LWSD	late tillering stage
sf0528903428	sf1127580568	2.94e-10	LWSD	late tillering stage
sf0405015647	sf0622511851	9.805e-05	$GLAR_4$	milk grain stage
sf0620800068	sf1212008851	4.921e-05	$GLAR_4$	milk grain stage
sf0702345742	sf0719554750	7.036e-18	LWSD	milk grain stage
sf0719554750	sf1126108571	4.765e-10	LWSD	milk grain stage
sf0401199801	sf1102574411	8.068e-07	APAR	milk grain stage
sf0305756733	sf1102574411	4.786e-07	APAR	milk grain stage
sf0114790584	sf0141826241	7.948e-07	APAR	milk grain stage
sf0114790584	sf1102574411	7.465e-18	APAR	milk grain stage
